# BAF60a-dependent chromatin remodeling preserves **β** cell function and contributes to the therapeutic benefits of GLP-1R agonists

**DOI:** 10.1172/JCI177980

**Published:** 2025-10-02

**Authors:** Xinyuan Qiu, Ruo-Ran Wang, Qing-Qian Wu, Hongxing Fu, Shuaishuai Zhu, Wei Chen, Wen Wang, Haide Chen, Xiuyu Ji, Wenjing Zhang, Dandan Yan, Jing Yan, Li Jin, Rong Zhang, Mengjie Shi, Ping Luo, Yingqing Yang, Qintao Wang, Ziyin Zhang, Wei Ding, Xiaowen Pan, Chengbin Li, Bin Liang, Guoji Guo, Hai-long Piao, Min Zheng, Sheng Yan, Lingyun Zhu, Cheng Hu, Zhuo-Xian Meng

**Affiliations:** 1 Department of Pathology and Pathophysiology and Department of Hepatobiliary and Pancreatic Surgery of the Second Affiliated Hospital, Zhejiang University School of Medicine, Hangzhou, Zhejiang, China.; 2 Department of Geriatrics, Hangzhou First People’s Hospital, Hangzhou, Zhejiang, China.; 3 College of Science and; 4 College of Computer Science and Technology, National University of Defense Technology, Changsha, Hunan, China.; 5 School of Pharmaceutical Science and Technology, Hangzhou Institute for Advanced Study, University of Chinese Academy of Sciences, Hangzhou, Zhejiang, China.; 6 Department of Hepatobiliary and Pancreatic Surgery of the Second Affiliated Hospital, Zhejiang University School of Medicine, Hangzhou, Zhejiang, China.; 7 Gan & Lee Pharmaceuticals, Beijing, China.; 8 CAS Key Laboratory of Separation Science for Analytical Chemistry, Dalian Institute of Chemical Physics, Chinese Academy of Sciences, Dalian, China.; 9 Center for Stem Cell and Regenerative Medicine and; 10 Department of Endocrinology and Metabolism, Sir Run Run Shaw Hospital, Zhejiang University School of Medicine, Hangzhou, Zhejiang, China.; 11 Shanghai Diabetes Institute, Shanghai Key Laboratory of Diabetes Mellitus, Shanghai Clinical Center for Diabetes, Department of Endocrinology and Metabolism, Shanghai Sixth People’s Hospital Affiliated to Shanghai Jiao Tong University School of Medicine, Shanghai, China.; 12 Department of Pathology, The First Affiliated Hospital, Zhejiang University School of Medicine, Hangzhou, China.; 13 Center for Life Sciences, School of Life Sciences, Yunnan University, Kunming, Yunnan, China.; 14 State Key Laboratory for Diagnosis and Treatment of Infectious Diseases, National Clinical Research Center for Infectious Diseases, Collaborative Innovation Center for Diagnosis and Treatment of Infectious Diseases, The First Affiliated Hospital, Zhejiang University School of Medicine, Hangzhou, Zhejiang, China.; 15 Institute for Metabolic Disease, Fengxian Central Hospital Affiliated to Southern Medical University, Shanghai, China.; 16 Affiliated Huzhou Hospital, Zhejiang University School of Medicine, Hangzhou, Zhejiang, China.

**Keywords:** Cell biology, Endocrinology, Beta cells, Diabetes, Insulin

## Abstract

Impaired glucose-stimulated insulin secretion (GSIS) is a hallmark of β cell dysfunction in diabetes. Epigenetic mechanisms govern cellular glucose sensing and GSIS by β cells, but they remain incompletely defined. Here, we found that BAF60a functions as a chromatin regulator that sustains biphasic GSIS and preserves β cell function under metabolic stress conditions. BAF60a was downregulated in β cells from obese and diabetic mice, monkeys, and humans. β cell–specific inactivation of BAF60a in adult mice impaired GSIS, leading to hyperglycemia and glucose intolerance. Conversely, restoring BAF60a expression improved β cell function and systemic glucose homeostasis. Mechanistically, BAF60a physically interacted with Nkx6.1 to selectively modulate chromatin accessibility and transcriptional activity of target genes critical for GSIS coupling in islet β cells. A BAF60a V278M mutation associated with decreased β cell GSIS function was identified in human donors. Mice carrying this mutation, which disrupted the interaction between BAF60a and Nkx6.1, displayed β cell dysfunction and impaired glucose homeostasis. In addition, GLP-1R and GIPR expression was significantly reduced in BAF60a-deficient islets, attenuating the insulinotropic effect of GLP-1R agonists. Together, these findings support a role for BAF60a as a component of the epigenetic machinery that shapes the chromatin landscape in β cells critical for glucose sensing and insulin secretion.

## Abstract 2

BAF60a regulates glucose-stimulated insulin-secretion function of pancreatic β-cells and contributes to the therapeutic function of GLP-1R agonists in a Nkx6.1-dependent manner.

## Graphical abstract

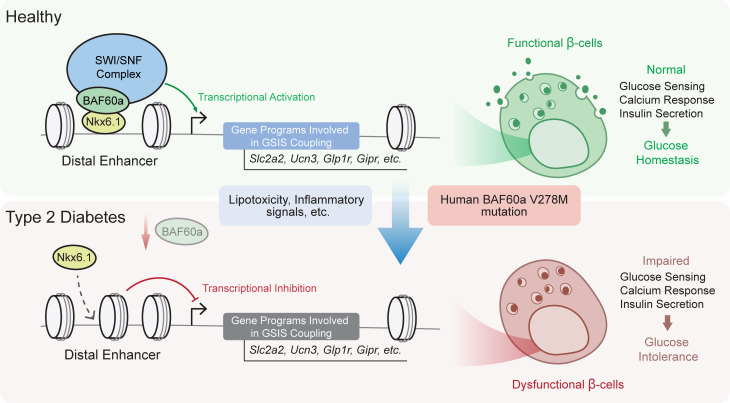

## Introduction

Type 2 diabetes (T2D) is a multifactorial metabolic disorder with increasing global prevalence that severely compromises human health (1, 2). Impaired insulin secretion and insulin resistance both contribute to T2D pathogenesis. Despite decades of research, the underlying mechanisms remain incompletely defined, and current therapies are suboptimal, with only 30%–50% of patients achieving glycemic control even with treatment (3).

Genetic and environmental factors influence T2D development. GWAS have identified more than 700 T2D risk loci (4–6), but these explain only a fraction of disease heritability and increasing prevalence. An increasing amount of evidence suggests epigenetic mechanisms, such as DNA methylation, histone modifications, RNA-mediated regulation, and chromatin remodeling, play essential roles in linking environmental cues to transcriptional programs involved in metabolic regulation (7–10). These mechanisms, characterized by their heritability and reversibility, are attractive for identifying biomarkers and therapeutic targets for precision treatment of T2D (10).

Notably, many T2D-associated genetic variants map to regulatory regions that control genes important for pancreatic islet β cell function (11), highlighting a central role for β cell dysfunction in T2D pathogenesis (11–14). While genetic variation partially contributes, epigenetic dysregulation of chromatin accessibility and transcription has emerged as a key mechanism underlying β cell failure in T2D. Recent high-throughput chromatin profiling approaches, such as assay for transposase-accessible chromatin using sequencing (ATAC-Seq), ChIP-Seq, and cleavage under targets and tagmentation (CUT&Tag**)**, have revealed widespread epigenomic alterations in β cells under diabetic conditions (14–18).

Dynamic chromatin accessibility is governed by ATP-dependent chromatin remodeling complexes, including SWI/SNF, ISWI, CHD, and INO80 (19). Among these, SWI/SNF complexes are critical regulators of nutrient sensing, energy metabolism, and cell fate (9, 19), altering nucleosome structure to enable transcription factor access to DNA. Remodeling at promoter and enhancer regions is highly context dependent, requiring the cooperation of transcription factors and chromatin remodelers (20). However, the identity of upstream chromatin-remodeling factors that integrate metabolic stress signals to reprogram transcriptional networks crucial for β cell function in T2D remains unclear.

In this study, we combine multi-omics profiling and functional screening to identify BAF60a, an SWI/SNF complex subunit, as a regulator of chromatin accessibility and transcriptional programs essential for β cell glucose sensing and insulin secretion. BAF60a expression is markedly reduced in islet β cells from obese and T2D mice, nonhuman primates, and humans. Pancreatic β cell–specific deletion of BAF60a (i.e., β cell–specific BAF60a KO [BaβKO]) leads to chromatin and transcriptional changes resembling those observed in diabetic islets, accompanied by impaired biphasic insulin secretion and glucose intolerance. Conversely, adeno-associated virus–mediated (AAV-mediated) restoration of BAF60a in diabetic mice improves β cell function and glucose homeostasis. Transplantation of BAF60a-overexpressing islets also enhances glycemic control in streptozotocin-treated (STZ-treated) mice.

Mechanistically, BAF60a physically interacts with the transcription factor Nkx6.1, guiding chromatin remodeling at gene loci involved in glucose metabolism and insulin secretion. We further identified a human BAF60a V278M mutation associated with impaired first-phase insulin secretion in an East Asian cohort. Mice harboring this mutation recapitulate key metabolic phenotypes, and the mutation disrupts BAF60a*-*Nkx6.1 interaction, impairing transcriptional activation of β cell genes. Additionally, we show that BAF60a deficiency reduces *Glp1r* and *Gipr* expression in islets and dampens the insulinotropic effect of semaglutide, suggesting that BAF60a may also contribute to the therapeutic effects of GLP-1R agonists.

Together, our findings define BAF60a as a key epigenetic checkpoint factor that integrates metabolic signals to remodel the chromatin landscape and coordinate transcriptional programs controlling β cell function in T2D.

## Results

### Identification of BAF60a as a key chromatin remodeling factor for β cell dysfunction in T2D.

**Figure 1:**
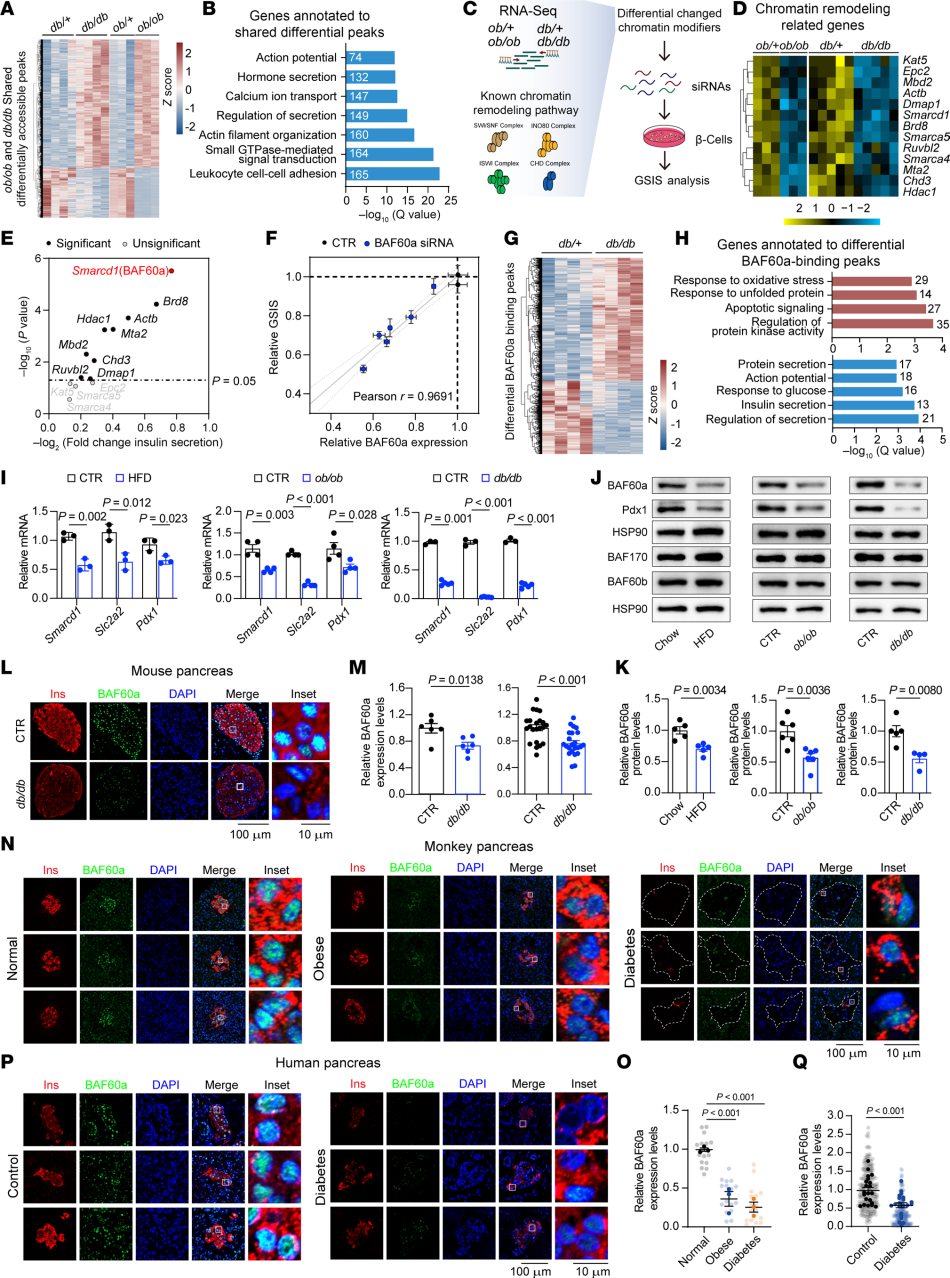
Multi-omics profiling identifies BAF60a inactivation as a key mediator of β cell dysfunction in T2D. (**A**) Heatmap of shared DAPs in islets from *ob/ob* and *db/db* mice. Scale bar: *Z* score. (**B**) GO analysis of genes annotated to DAPs in (**A**). (**C**) Schematic of siRNA screening pipeline. (**D**) Heatmap of chromatin remodeling genes downregulated in *ob/ob* (*n* = 3) and *db/db* (*n* = 5) islets. Scale bar: *Z* score. (**E**) Scatter plot of GSIS changes in Min6 cells transfected with siRNAs (*n* = 6 per gene). Significant hits are indicated in black, BAF60a in red. Control siRNA used for normalization. (**F**) Correlation between relative *BAF60a* expression and GSIS. Linear regression is shown with 95% CI (dashed). Mean ± SEM; *n* = 6 biological replicates. (**G**) Heatmap of altered BAF60a-binding peaks in *db/db* islets (|log2FC| > 0.5; FDR < 0.05). (**H**) GO analysis of genes linked to more-accessible (red) or less-accessible (blue) BAF60a-binding peaks. **(I**) Relative *BAF60a*, *Slc2a2*, and *Pdx1* mRNA in HFD (*n* = 3), *ob/ob* (*n* = 4), and *db/db* (*n* = 3–5) islets. Mean ± SEM; 2-tailed unpaired Student’s *t* test. (**J** and **K**) Immunoblot and quantification of islet lysates (pooled from 3 mice/group). Mean ± SEM; 2-tailed unpaired Student’s *t* test. (**L** and **M**) Representative immunofluorescence images and quantification of mouse pancreas sections (*n* = 6). Scale bars: 100 μm and 10 μm. Quantification is shown per mouse (left) and per islet (right). Mean ± SEM; 2-tailed unpaired Student’s *t* test. (**N**–**Q**) Representative immunofluorescence images and quantification of monkey (*n* = 3/group in **N** and **O**) and human (*n* = 21/group in **P** and **Q**) pancreas sections. Scale bars: 100 μm and 10 μm. Gray dots indicate islets; colored dots indicate monkey or human individuals. Mean ± SEM; *P* values were determined by 1-way ANOVA (monkey individuals) or 2-tailed unpaired Student’s *t* test (human individuals). CTR, control; INS, insulin.

Global chromatin accessibility changes in pancreatic β cells have been widely reported in both humans with diabetes and murine models of the disease (14, 16–18, 21). Consistent with these findings, our ATAC-Seq analysis of islets from *ob/ob* and *db/db* mice revealed widespread alterations in chromatin accessibility relative to littermate controls (Figure 1A). We identified 43,867 and 22,963 differentially accessible peaks (DAPs) (|log_2_ fold change [log_2_FC]| > 0.5; FDR < 0.05) in *ob/ob* and *db/db* islets, respectively, with 8,163 peaks showing shared directional changes. Among these, 5,876 regions exhibited increased accessibility and 2,287 were less accessible, mapping to 4,809 genes. Gene Ontology (GO) analysis showed enrichment in pathways essential for β cell function, including insulin secretion, glucose homeostasis, and calcium ion regulation (Figure 1B). Notably, most of these regions were located within distal enhancer-like signature (dELS), suggesting that altered enhancer accessibility contributes to β cell dysfunction in T2D (Supplemental Figure 1A; supplemental material available online with this article; https://doi.org/10.1172/JCI177980DS1).

To identify upstream regulators of these chromatin changes, we performed transcriptomic profiling of islets from *ob/ob* and *db/db* mice. A combined analysis of RNA-Seq (*ob/ob*) and published microarray data (*db/db*; Gene Expression Omnibus GSE31953) revealed 668 coregulated genes, including 329 downregulated genes enriched for terms related to insulin secretion, glucose metabolism, and chromatin remodeling (Supplemental Figure 1B). These findings raise the possibility that downregulation of chromatin modifiers may contribute to β cell dysfunction under diabetic conditions.

Focusing on chromatin remodeling genes, we identified 13 of 74 known murine regulators (22) that were consistently downregulated in both models (Figure 1D). To evaluate their functional relevance, we performed a small-scale siRNA screen in Min6 cells targeting these candidates (Figure 1C). Knockdown of several factors, including *BAF60a*, *Brd8*, *Hdac1*, *Dmap1*, and others, led to a statistically significant reduction in glucose-stimulated insulin secretion (GSIS) (*P* < 0.05) (Figure 1E, Supplemental Figure 1, C and D, and Supplemental Table 1). Among these, *BAF60a* knockdown led to a 46.1% reduction in GSIS, and further experiments with independent siRNAs confirmed a strong correlation between *BAF60a* gene expression and GSIS capacity (*r* = 0.9691; *P* < 0.0001) (Figure 1F and Supplemental Figure 1, E and F), indicating a potential dose-dependent role of BAF60a in supporting β cell function.

We next examined whether BAF60a binds to chromatin regions altered in diabetic islets. CUT&Tag profiling in Min6 cells identified 16,885 BAF60a-binding peaks with >6-fold signal over BAF60a KO controls. These peaks were significantly enriched within the differentially accessible regions found in *ob/ob* and *db/db* islets (fold enrichment = 1.17; *P* = 1 × 10^–14.5^). In *db/db* islets, we observed increased accessibility at 996 BAF60a-bound loci and decreased accessibility at 575 loci (Figure 1G). Regions with enhanced accessibility were enriched for stress-related pathways (e.g., oxidative stress, unfolded protein response). In contrast, those with reduced accessibility were associated with insulin secretion, glucose sensing, and membrane excitability (Figure 1H).

Consistent with these chromatin changes, *BAF60a* mRNA was significantly reduced in islets from high-fat diet–fed (HFD-fed), *ob/ob*, and *db/db* mice, along with decreased expression of β cell identity and function genes such as *Slc2a2* and *Pdx1* (Figure 1, I–K). Western blotting and immunofluorescence confirmed a marked reduction of BAF60a protein in islets from diabetic mice (Figure 1, J–M), obese/diabetic monkeys (Figure 1, N and O), and patients with T2D (Figure 1, P and Q, and Supplemental Figure 1G).

Together, these data support a model in which downregulation of BAF60a under diabetic conditions contributes to β cell dysfunction by altering chromatin accessibility at genes critical for insulin secretion and glucose responsiveness.

### β cell–specific BAF60a inactivation impairs insulin secretion and systemic glucose homeostasis.

**Figure 2:**
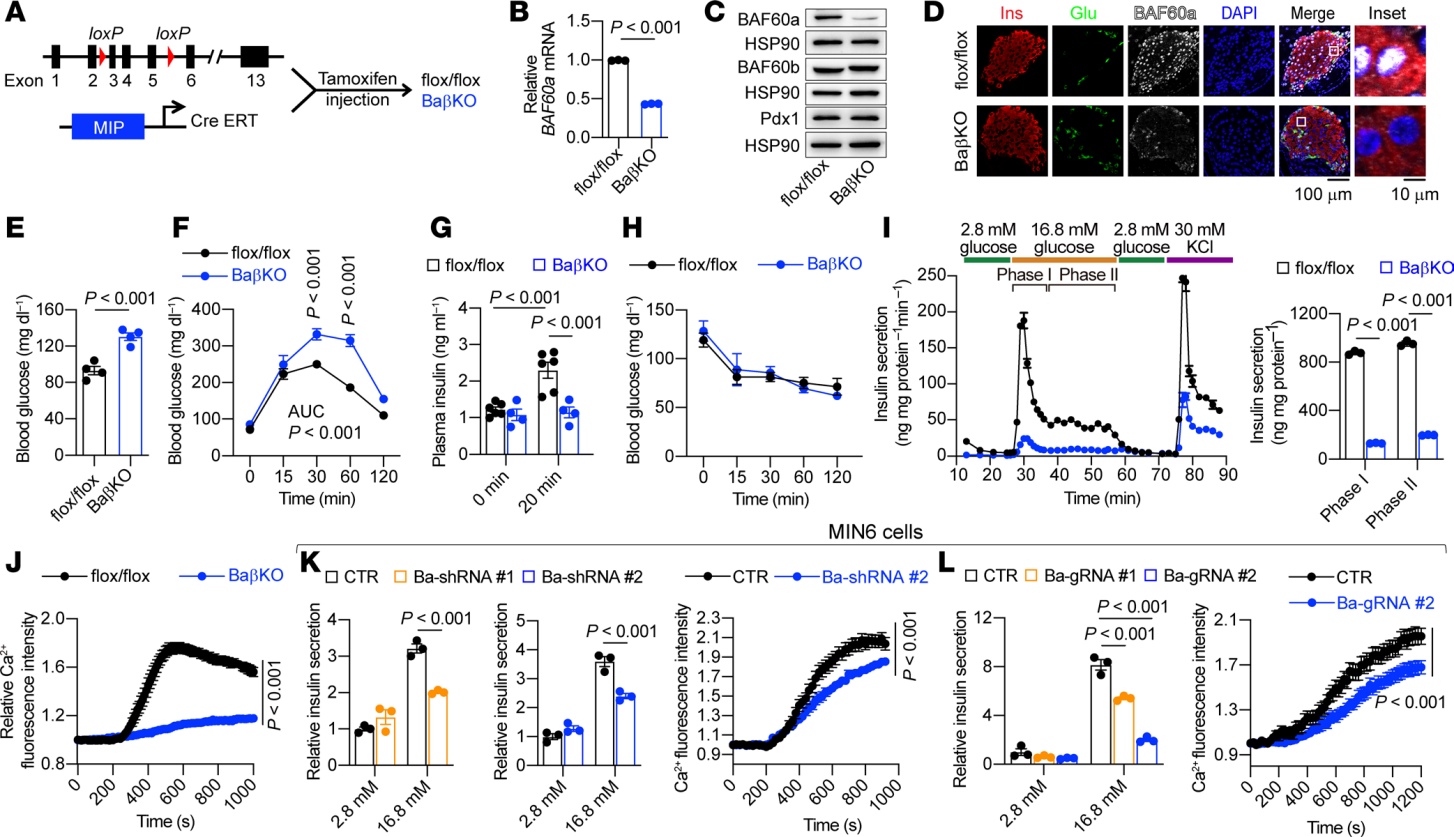
Ablation of BAF60a in β cells impairs insulin (Ins) secretion and glucose (Glu) homeostasis. (**A**) Workflow for generating BaβKO mice. (**B**) Relative *BAF60a* mRNA in flox/flox and BaβKO islets. Mean ± SEM; *n* = 3–4/group; 2-tailed unpaired Student’s *t* test. (**C**) Immunoblot of islet lysates (pooled from 3 mice/group). (**D**) Pancreas sections stained with indicated antibodies. Scale bar: 100 μm and 10 μm. (**E**) Short-fast blood glucose levels. Mean ± SEM; *n* = 4/group; 2-tailed unpaired Student’s *t* test. (**F**) OGTT results. Mean ± SEM; *n* = 4–5/group; 2-way ANOVA. (**G**) In vivo GSIS results. Mean ± SEM; *n* = 4–6/group; 2-way ANOVA. (**H**) ITT results. Mean ± SEM; *n* = 4–5 per group. (**I**) Dynamic GSIS and biphasic insulin release from islets pooled from 3 mice/group. Mean ± SD; *n* = 3 technical replicates; 2-tailed unpaired Student’s *t* test. (**J**) Intracellular Ca²^+^ influx in response to 16.8 mM glucose. Islets pooled from 3 mice. Mean ± SEM; *n* = 42–49 islets/group; 2-way ANOVA. (**K** and **L**) GSIS (left) and Ca²^+^ influx (right) in Min6 cells with BAF60a knockdown (**K**) or KO (**L**). Mean ± SEM; GSIS: *n* = 3 biological replicates; Ca²^+^ influx: *n* = 94–96 (**K**) or 46–51 (**L**) cells/group; 2-way ANOVA. CTR, control.

Given the marked downregulation of BAF60a in diabetic β cells, we generated BaβKO mice by crossing BAF60a^flox/flox^ with MIP-CreERT mice. Tamoxifen induction at 12 weeks effectively reduced BAF60a expression in islets, whereas expression in other tissues remained unaffected (Figure 2, A and B, and Supplemental Figure 2A). Western blot and immunofluorescence confirmed BAF60a protein loss specifically in insulin-producing β cells (Figure 2, C and D).

Intriguingly, BaβKO mice fed a chow diet had elevated fasting glucose levels and impaired glucose tolerance and in vivo GSIS (Figure 2, E–G), whereas insulin sensitivity remained unchanged (Figure 2H). Pancreatic histology and islet size were unaffected (Supplemental Figure 2B), and electron microscopy showed a minor reduction in the immature/total insulin granule ratio without altering overall granule number (Supplemental Figure 2C). Under HFD feeding, glucose intolerance of BaβKO mice worsened, again without changes in insulin sensitivity (Supplemental Figure 2D). These findings suggest BAF60a inactivation impairs glucose homeostasis primarily through defective β cell secretory function.

Dynamic perifusion studies revealed that BaβKO islets had markedly reduced first- and second-phase insulin secretion under both chow and HFD conditions (Figure 2I and Supplemental Figure 2E). Correspondingly, glucose-stimulated calcium influx was substantially reduced (Figure 2J). These phenotypes were also observed in an independent β cell–specific KO line (BaβKORIP) (Supplemental Figure 2, F–K). To examine cell-autonomous effects, we generated Min6 cell lines with BAF60a KO with CRISPR-Cas9 or knockdown via shRNA (Supplemental Figure 2, L and M). Both models showed impaired GSIS (1.5- to 4.1-fold reduction; *P* < 0.001) and diminished calcium influx (Figure 2, K and L), consistent with in vivo findings.

**Figure 3:**
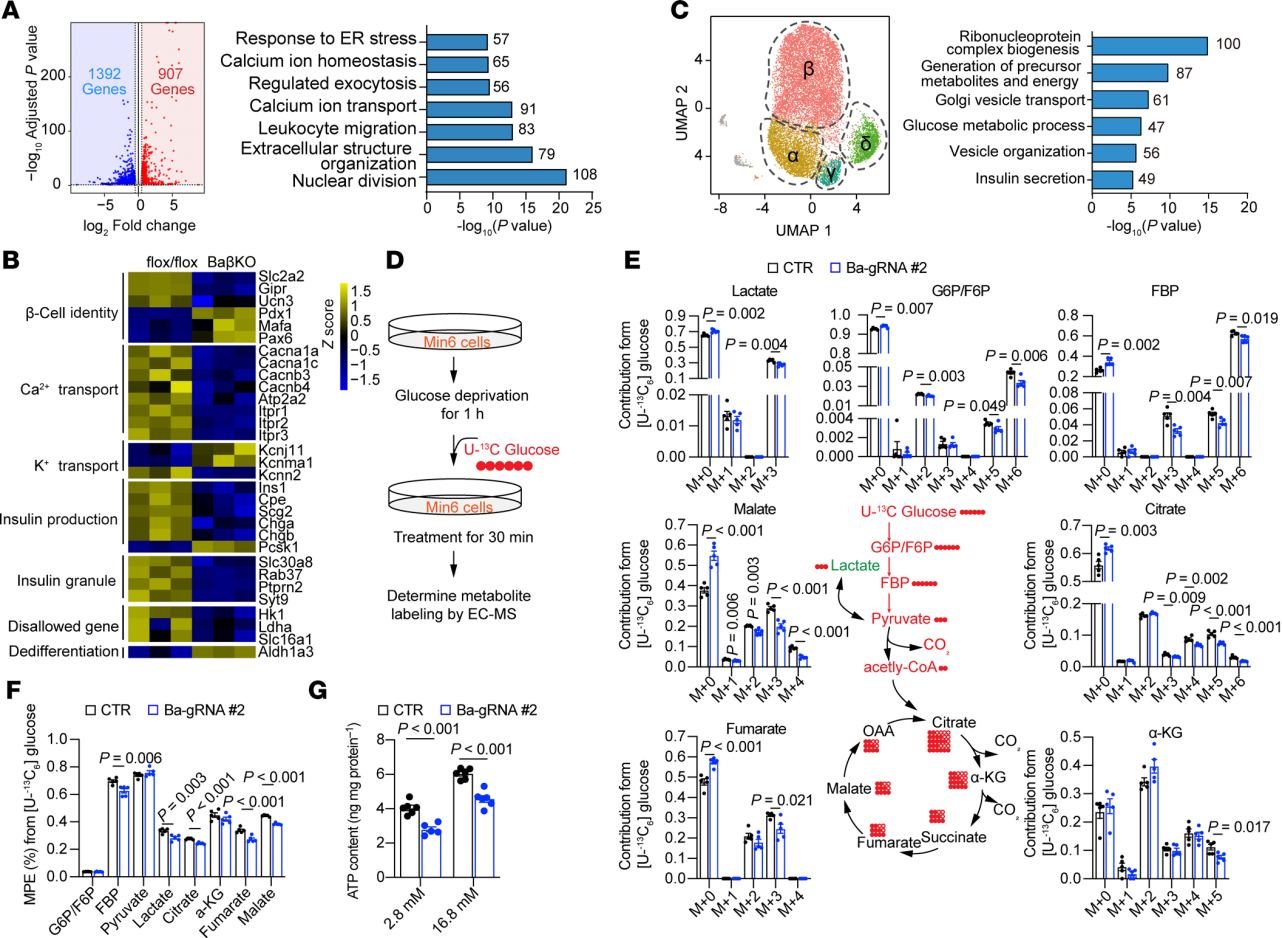
BAF60a regulates β cell function by modulating glucose metabolism–related genes. (**A**) Left: Differential expression analysis of RNA-Seq from flox/flox and BaβKO islets (RNA pooled from 3 mice; *n* = 3 biological replicates; |log2FC| > 0.5; FDR < 0.05). Right: GO analysis of significantly differentially expressed genes (DEGs) showing top nonredundant biological processes with gene counts and *P* values. (**B**) Heatmap of selected pathway-associated genes from (**A**). (**C**) Left: Uniform Manifold Approximation and Projection (UMAP) visualization of transcriptome-based clustering in flox/flox and BaβKO islets. Islets pooled from 3 mice; 4,077 and 4,078 cells analyzed, respectively. Right: GO analysis of DEGs in β cell cluster with top biological processes. (**D**) Workflow of ^13^C-glucose metabolic flux analysis in Min6 cells. (**E**) ^13^C-glucose tracing in control (CTR) and BAF60a-KO Min6 cells. M, unlabeled metabolite; M+n, metabolite mass with *n* labeled carbons. Mean ± SEM; *n* = 5 biological replicates; 2-tailed unpaired Student’s *t* test. (**F**) Molar percent enrichment (MPE) of ^13^C in glycolysis and TCA metabolites in CTR and BAF60a-KO Min6 cells. Mean ± SEM; *n* = 5 biological replicates; 2-tailed unpaired Student’s *t* test. (**G**) Intracellular ATP content in CTR and BAF60a-KO Min6 cells stimulated with 16.8 mM glucose. Mean ± SEM; *n* = 5–6 biological replicates; 2-way ANOVA.

We next performed bulk RNA-Seq on islets from BaβKO and control mice 4 months after tamoxifen injection. A total of 2,299 genes were differentially expressed (|log_2_FC| > 0.5; FDR < 0.05), including 1,392 upregulated and 907 downregulated genes (Figure 3A). GO enrichment analysis showed dysregulation of pathways essential for β cell function, including insulin secretion, calcium transport, glucose homeostasis, and ER stress. Downregulated genes were enriched for insulin biosynthesis, granule formation, and calcium signaling; upregulated genes included potassium transporters and the dedifferentiation marker *Aldh1a3* (Figure 3B). These transcriptomic changes likely contribute to the diabetic phenotype in BaβKO mice. Comparative analysis with the *ob/ob* model revealed a shared downregulation of genes involved in insulin secretion and glucose regulation (Supplemental Figure 3B), suggesting BAF60a deficiency alone can recapitulate key molecular features of T2D.

To further investigate cellular heterogeneity, we performed single-cell RNA-Seq (scRNA-Seq) on BaβKO and control islets. Endocrine cell types were identified by expression of marker genes (*Ins1*, *Gcg*, *Sst*, and *Ppy*) (Figure 3C and Supplemental Figure 3C). BaβKO islets showed a modest reduction in β and pancreatic polypeptide cell proportions and an increase in α and δ cells, suggesting potential dedifferentiation or trans-differentiation events (Supplemental Figure 3C). GO analysis of differentially expressed genes within β cell clusters confirmed the downregulation of insulin secretion–related genes in BaβKO β cells (Figure 3C).

Because GSIS is tightly coupled to glucose metabolism, we next examined whether BAF60a deficiency alters glycolytic flux. Using uniformly labeled [U-^13^C] glucose tracing in BaKO Min6 cells, we observed reduced enrichment of mass units plus 6 (M+6) isotopologues for G6P/F6P and citrate, M+5 for α-ketoglutarate, and M+3 for fumarate, malate, and lactate, indicating impaired glycolytic and TCA cycle activity (Figure 3, D–F). Consistently, ATP levels were markedly decreased under both basal (2.8 mM) and stimulatory (16.8 mM) glucose conditions (Figure 3G). Because ATP generation is necessary for membrane depolarization and calcium influx, these findings suggest BAF60a supports β cell function in part by maintaining glucose metabolic activity.

### AAV-mediated overexpression of BAF60a preserves β cell function and glucose homeostasis in diabetic mice.

**Figure 4:**
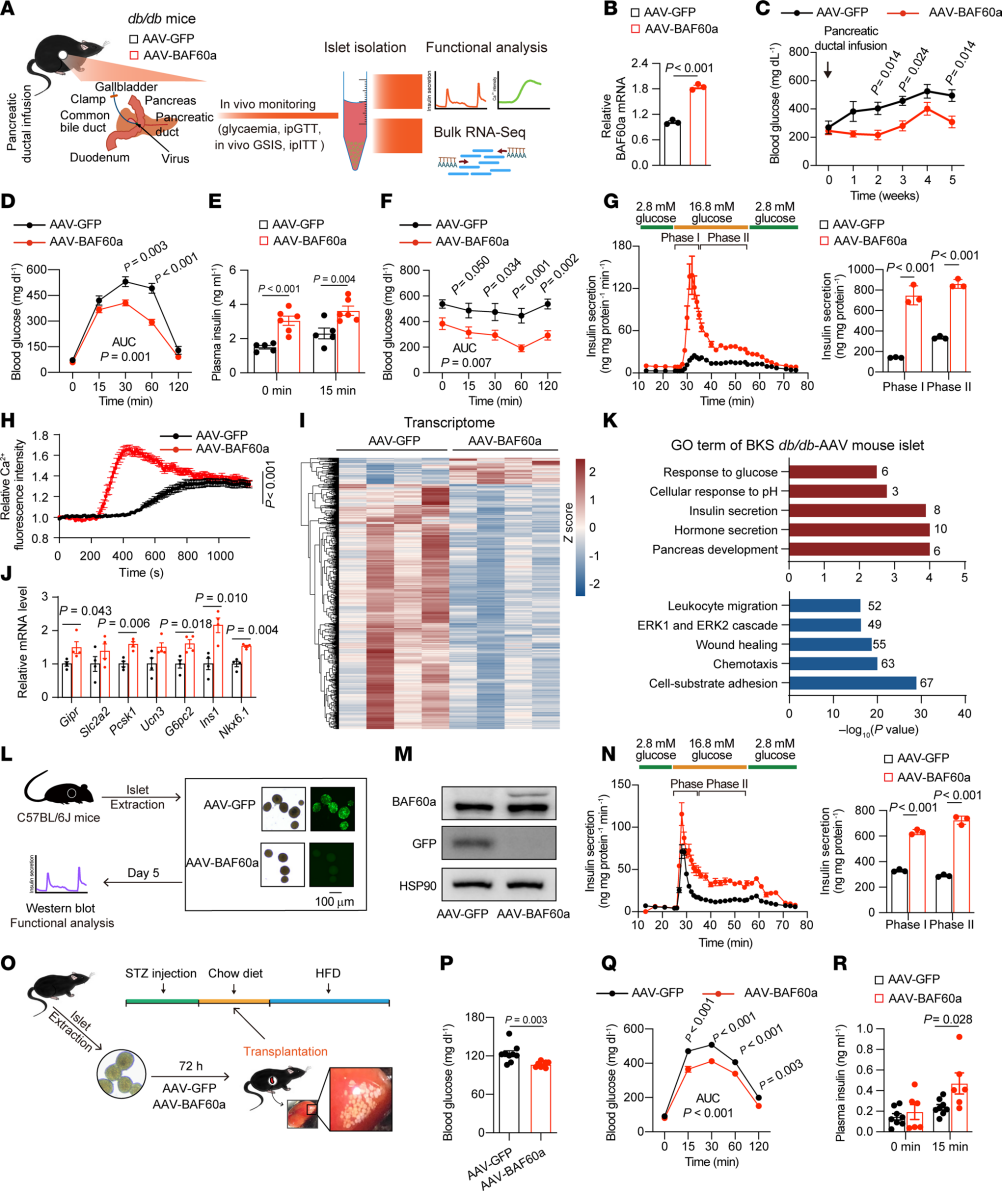
AAV-mediated overexpression of BAF60a preserves β cell function and glucose homeostasis in diabetic mice. (**A**) Pancreatic ductal AAV infusion and experimental design for (**B**–**K**). (**B**) Relative *BAF60a* mRNA in islets pooled from 3 mice/group. Mean ± SD; *n* = 3 technical replicates; 2-tailed unpaired Student’s *t* test. (**C**–**F**) Random glucose (**C**), ipGTT (**D**), in vivo GSIS (**E**), and ITT (**F**) in BKS-*db/db* mice. Mean ± SEM; *n* = 5–6 (**C**, **E**, and **F**) or *n* = 6 (**D**); 2-way ANOVA. (**G**) Biphasic insulin secretion in islets pooled from 3 mice/group. Mean ± SD; *n* = 3 technical replicates; 2-tailed unpaired Student’s *t* test. (**H**) Intracellular Ca²^+^ influx in islets pooled from 3 mice/group. Mean ± SEM; *n* = 32–33 islets; 2-way ANOVA. (**I**–**K**) Transcriptomics of AAV-GFP– versus AAV-BAF60a–treated islets, heatmap (**I**), relative mRNA expression (**J**), and GO analysis (**K**) of differentially expressed genes. (**L**–**N**) Design (**L**), immunoblot (**M**), and biphasic insulin secretion (**N**) in islets pooled from 3 mice/group. Mean ± SD; *n* = 3 replicates; 2-tailed unpaired Student’s *t* test. (**O**–**R**) Design (**O**), fasting blood glucose (**P**), OGTT (**Q**), and in vivo GSIS (**R**) in STZ-treated and islet-transplanted mice. Mean ± SEM; *n* = 6–9 (**P** and **Q**) or 6–8 (**R**); 2-tailed unpaired Student’s *t* test (**P**) or 2-way ANOVA (**Q** and **R**).

To examine the impact of BAF60a gain of function, we infused *BAF60a*-expressing AAV into the pancreatic ducts of BKS-*db/db* mice (Figure 4A). Western blotting, qPCR, and immunohistochemistry confirmed robust BAF60a overexpression in islets (Figure 4B and Supplemental Figure 4, A and B). Mice receiving AAV-CAG-BAF60a exhibited attenuated hyperglycemia progression (Figure 4C) and had improved glucose tolerance and in vivo GSIS (Figure 4, D and E). Interestingly, insulin sensitivity was also improved (Figure 4F), likely due to broader transgene expression from the CAG promoter (Supplemental Figure 4, C–E).

To evaluate whether these effects were β cell specific, we used AAV-RIP-BAF60a, which restricts expression to β cells via the rat insulin promoter (23). In this model, glucose homeostasis and GSIS were improved (Supplemental Figure 5, B and E), while body weight and insulin sensitivity remained unaffected (Supplemental Figure 5, C and D), suggesting systemic metabolic effects seen with CAG-driven BAF60a may involve extra–β cell tissues (Supplemental Figure 4F and Supplemental Figure 5A).

Perifusion assays confirmed that BAF60a overexpression, via either CAG or RIP constructs, enhanced first- and second-phase insulin secretion (~5.2-fold and ~2.6-fold; *P* < 0.001) (Figure 4G and Supplemental Figure 5E). Islets from treated mice also had elevated glucose-stimulated Ca²^+^ influx (Figure 4H). Transcriptomic profiling revealed upregulation of β cell identity and function genes (e.g., *Slc2a2*, *Ucn3*, *Nkx6.1*, *Gipr*, *Pcsk1*, *G6pc2*) (Figure 4, I and J), with GO enrichment highlighting enhanced pathways in insulin secretion, calcium signaling, and glucose sensing, and repression of inflammatory and adhesion-related programs (Figure 4K). These results suggest BAF60a overexpression restores β cell function in diabetic islets by reprogramming transcriptional networks and improving calcium signaling.

Islet transplantation has been recognized as an effective strategy for treating diabetes (24). However, human islet transplantation has been hampered by donor cell death associated with the islet preparation procedure before transplantation (25) One potential solution is AAV-mediated gene therapy of primary islets to enhance β cell function and its tolerance against the ex vivo culturing condition before transplantation. Hence, we tested whether AAV-mediated BAF60a overexpression could enhance function in cultured islets (Figure 4L). As shown in Figure 4M, Western blot analysis confirmed that AAV-BAF60a treatment led to a successful overexpression of BAF60a in islets isolated from C57BL/6J mice and cultured ex vivo for 5 days. Moreover, BAF60a overexpression increased phase I and II insulin secretion (~1.92-fold and ~2.50-fold; *P* < 0.001 and *P* < 0.0001, respectively) compared with AAV-GFP treated islets (Figure 4N). To assess whether BAF60a-enhanced islets improve transplantation outcomes, we transplanted AAV-treated islets into STZ-induced diabetic mice (Figure 4O). Mice receiving AAV-BAF60a islets had improved fasting glucose levels, enhanced glucose tolerance, and restored in vivo GSIS compared with controls (Figure 4, P–R), demonstrating the potential utility of *BAF60a* gene therapy in islet replacement strategies.

### BAF60a modulates β cell function via Nkx6.1-dependent chromatin remodeling.

**Figure 5:**
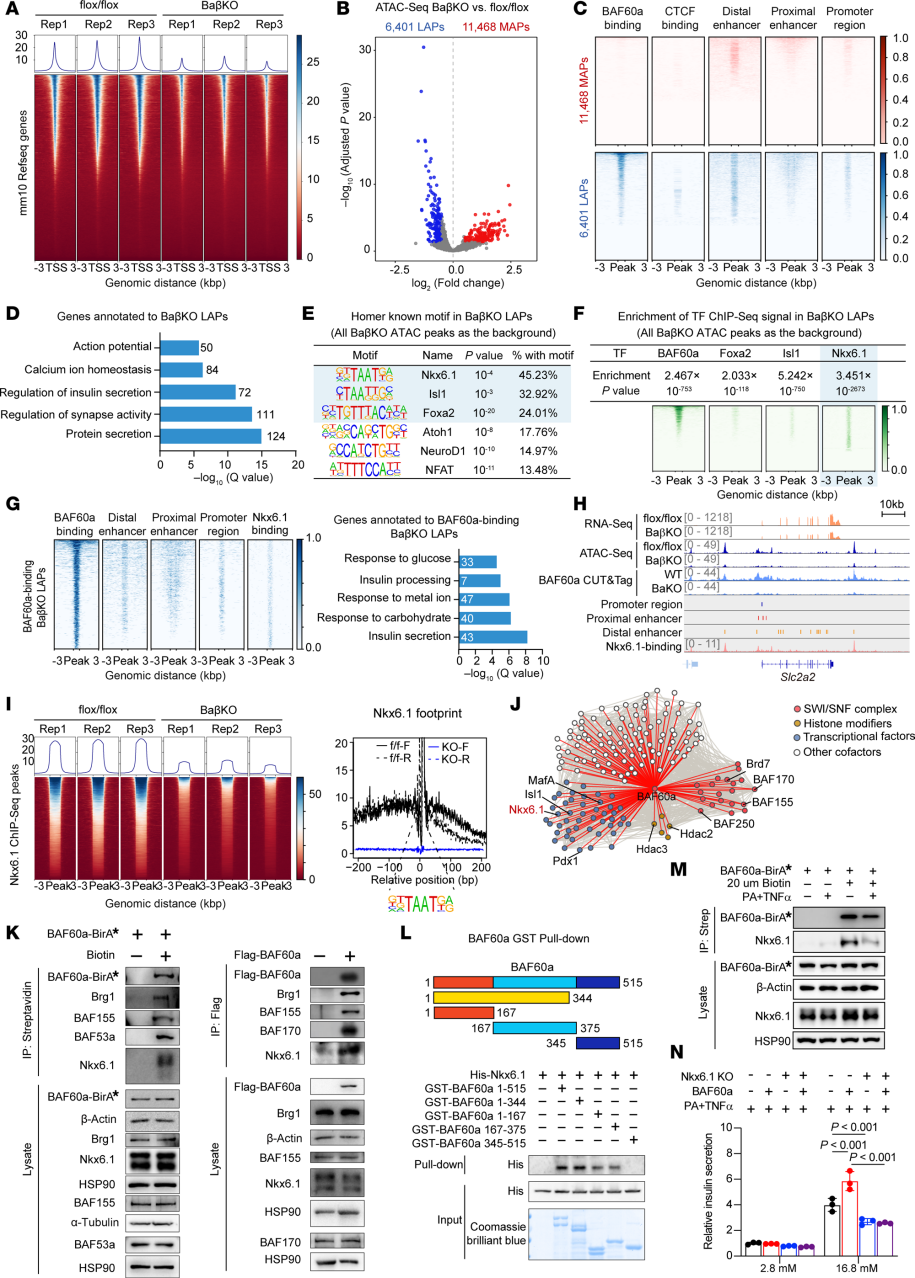
BAF60a modulates β cell function via Nkx6.1-dependent chromatin remodeling. (**A**) Metagene heatmap of ATAC-Seq signals across –3 kb to +3 kb of TSS (*n* = 3 biological replicates). (**B**) Differential accessibility analysis of ATAC peaks. MAPs (red) and LAPs (blue) defined by |log2FC| > 0.5, FDR < 0.05 (*n* = 3). (**C**) Metagene heatmap of BAF60a CUT&Tag-Seq signals and the Encyclopedia of DNA Elements (ENCODE) cis-regulatory elements around MAPs (red) and LAPs (blue). (**D**) GO enrichment of genes linked to LAPs. (**E**) Motif enrichment within LAPs with consensus sequence, transcription factor (TF) name, *P* value, and percentage of peaks containing motif. (**F**) ChIP-Seq enrichment of TFs at LAPs with fold enrichment and hypergeometric *P* values. (**G**) CUT&Tag-Seq heatmap and GO analysis of BAF60a-binding LAPs. (**H**) Browser tracks of Slc2a2 locus from RNA-Seq, ATAC-Seq, and CUT&Tag-Seq. (**I**) ATAC-Seq signal distribution at Nkx6.1-binding peaks (left) and TF footprinting of Nkx6.1 (right). (**J**) BAF60a interactome in Min6 cells. Red lines from BioID–mass spectrometry analysis, and gray lines from STRING datasets. (**K** and **L**) Immunoblots of BAF60a interactomes detected by BioID/Flag IP (**K**), and GST pull-down with GST-tagged BAF60a truncated proteins and His-Nkx6.1 (**L**). (**M**) BioID-immunoblots of BAF60a-Nkx6.1 interaction with or without TNF-α (50 ng/mL) and PA (0.5 mM) treatment. (**N**) GSIS in Min6 cells overexpressing BAF60a with or without Nkx6.1 KO under PA/TNF-α treatment. Mean ± SEM; *n* = 3 biological replicates; 2-way ANOVA. Strep, streptavidin.

BAF60a facilitates the recruitment of SWI/SNF complexes to specific genomic loci to regulate chromatin accessibility and gene expression (9, 26). To examine chromatin accessibility changes following BAF60a deficiency, we performed ATAC-Seq on islets from BaβKO and control mice. Genome-wide analysis revealed a broad reduction in chromatin accessibility surrounding transcription start sites (±3 kb from transcriptional start sites [TSSs]) in BaβKO islets (Figure 5A). Among the 85,691 accessible chromatin regions identified, 6,401 had reduced accessibility, termed *less-accessible peaks* (LAPs), and 11,468 had increased accessibility (more-accessible peaks [MAPs]) (Figure 5B). Notably, LAPs were substantially enriched for BAF60a binding, whereas MAPs lacked such enrichment (Figure 5C), indicating that BAF60a inactivation predominantly results in reduced accessibility at its target loci. Further annotation of these altered regions showed enrichment of distal enhancers and promoters within LAPs, especially those bound by BAF60a (Figure 5C), supporting the role of BAF60a as a positive regulator of enhancer and promoter accessibility. In contrast, MAPs were associated with distinct regulatory signatures.

GO analysis of LAP-associated genes revealed enrichment in processes essential for β cell function, including insulin secretion, calcium ion homeostasis, and regulation of membrane potential (Figure 5D), aligning with the GSIS impairment observed in BaβKO islets (Figure 2, I and J). By contrast, MAP-associated genes were largely linked to developmental and stress-related pathways (Supplemental Figure 6A). These findings suggest BAF60a loss disrupts β cell function primarily by reducing accessibility at regulatory elements controlling glucose metabolism and insulin exocytosis.

Given that BAF60a lacks a DNA-binding domain, it likely operates through interactions with β cell transcription factors (9). Motif enrichment analysis of LAPs identified overrepresentation of motifs for transcription factors Nkx6.1, Isl1, Foxa2, and NeuroD1 (Figure 5E). When we cross-referenced with published ChIP-Seq datasets (27–29), we found that LAPs were enriched for binding by Foxa2, Isl1, and especially Nkx6.1 (2.03-, 5.24-, and 3.45-fold enrichment, respectively), with Nkx6.1 having the strongest association (Figure 5F).

Importantly, BAF60a-bound LAPs, likely the primary epigenomic targets of BAF60a, were found to co-occupy regions with Nkx6.1 binding, particularly within distal enhancers and promoter elements (Figure 5G). These regions mapped to genes involved in glucose responsiveness, metal ion transport, and insulin production (Figure 5, G and H). Restoration of BAF60a in BaβKO islets using AAV largely recovered accessibility at these regions (Supplemental Figure 6B), highlighting the role of BAF60a in maintaining transcriptional competence of β cell regulatory elements.

Consistent with these findings, ATAC-Seq signal intensity at Nkx6.1 binding sites was markedly reduced in BaβKO islets (Figure 5I), and transcription factor footprinting showed decreased accessibility near Nkx6.1 motifs (Figure 5I). This suggests potential spatial and functional cooperation between BAF60a and Nkx6.1.

To identify BAF60a interactome, we performed BioID-based proteomics (30) in Min6 cells expressing BAF60a-BirA* fusion protein. Mass spectrometry identified multiple SWI/SNF subunits (e.g., BAF170, BAF155, BAF250) as well as transcription factors (e.g., Nkx6.1), histone modifiers (e.g., Hdac2, Hdac3), and additional cofactors (Figure 5J). Co-IP and glutathione *S*-transferase (GST) pull-down assays confirmed that BAF60a interacts with Nkx6.1, likely through its 1–167 and 167–344 domains (Figure 5, K and L). These findings support a model in which BAF60a cooperates with Nkx6.1 to control accessibility and expression of genes critical for β cell function.

Consistent with the central role of Nkx6.1 in β cell physiology (29), CRISPR-mediated depletion of Nkx6.1 led to impaired GSIS in Min6 cells (Supplemental Figure 6C). CUT&Tag profiling in Nkx6.1-KO Min6 cells revealed substantially reduced BAF60a occupancy (Supplemental Figure 6D), implying that Nkx6.1 is required for recruiting or stabilizing BAF60a at target loci. Genome browser analysis showed loss of accessibility and transcriptional downregulation at *Slc2a2*, *Cpe*, *Scg2*, *Chga*, and *Chgb* gene loci, all of which are co-occupied by BAF60a and Nkx6.1 (Figure 5H and Supplemental Figure 6E).

To identify upstream stress signals driving BAF60a suppression in T2D β cells, we treated Min6 cells with glucose, fatty acids, hormones, or cytokines. We found that palmitate acid (PA), but not oleate acid, reduced *BAF60a* gene expression by 39.3% (*P* = 0.001), and this effect was amplified with TNF-α cotreatment (Supplemental Figure 6, F and G). This PA plus TNF-α combination, which mimics lipotoxic inflammation, also impaired BAF60a-Nkx6.1 interaction (Figure 5M and Supplemental Figure 6H). BAF60a overexpression rescued GSIS under PA plus TNF-α conditions, but this rescue was abolished by Nkx6.1 KO (Figure 5N).

Together, these data demonstrate BAF60a regulates β cell transcriptional programs via physical and functional interactions with Nkx6.1, orchestrating chromatin remodeling at key gene loci involved in glucose metabolism and insulin secretion.

### Human BAF60a^V278M^ mutation impairs β cell insulin secretion.

**Figure 6:**
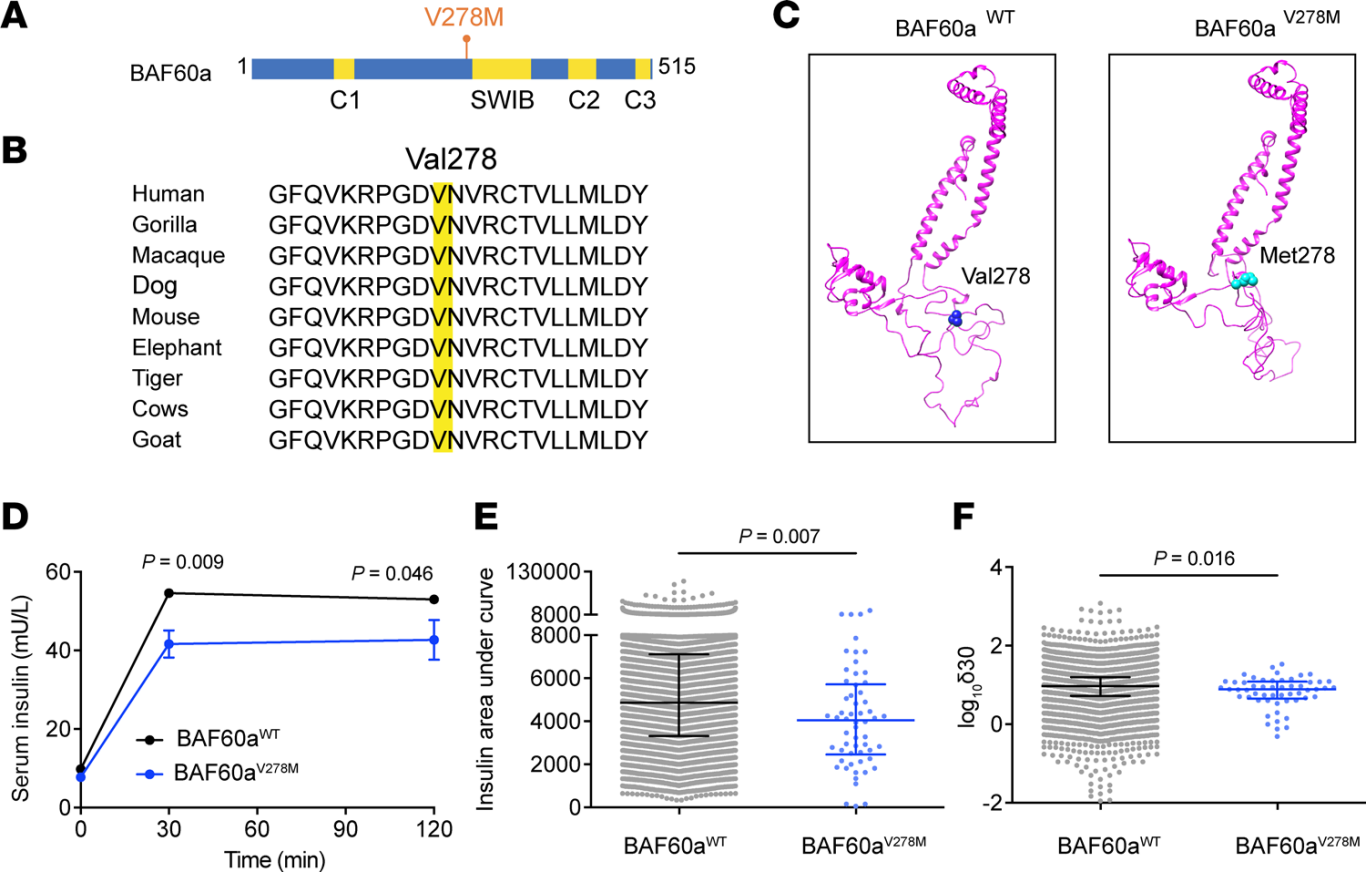
Human BAF60a^V278M^ mutation impairs β cell insulin secretion. (**A**) Domain structure of human BAF60a showing SWIB and predicted coiled-coil domains (C1–C3, orange) and variant position. (**B**) Evolutionary conservation of V278 across species. (**C**) Predicted 3D models of BAF60a^WT^ and BAF60a^V278M^. (**D**) Serum insulin levels of BAF60a^WT^ and BAF60a^V278M^ groups after OGTT assays. Mean ± SEM (*n* ≥ 13,777 people in BAF60a^WT^ group and ≥57 people in BAF60a^V278M^ group); *P* value was determined by a nonparametric test (Mann-Whitney test) for a skewed distribution. (**E**) AUC of serum insulin levels in (**D**). Values are presented as median with interquartile range (*n* = 13,766 people in BAF60a^WT^ group and 58 people in BAF60a^V278M^ group); 2-tailed unpaired Student’s *t* test (**F**) The difference of δ30 [(30-minute serum insulin – fasting serum insulin)/(30-minute plasma glucose – fasting plasma glucose)] in (**D**). Values are presented as median with interquartile range (*n* = 13,376 people in the BAF60a^WT^ group and 54 people in the BAF60a^V278M^ group); 2-tailed unpaired Student’s *t* test.

To evaluate the clinical relevance of BAF60a dysfunction in humans, we examined potential diabetes-associated variants of BAF60a. A heterozygous p.V278M substitution (c.832G>A [rs200921207]) in exon 7 (hereafter referred to as BAF60a^V278M^) was identified and found to be associated with β cell GSIS function (Figure 6A). This valine-to-methionine change occurs within a highly conserved region between the C1 and SWIB domains of BAF60a (Figure 6, A and B), and structural modeling using SWISS-MODEL suggested that the V278M mutation may alter the 3D structure of BAF60a (Figure 6C).

Of 13,903 individuals in a community-based cohort, we identified 61 heterozygous BAF60a^V278M^ carriers (minor allele frequency 0.22%) (Supplemental Table 3). Compared with noncarriers, BAF60a^V278M^ carriers had significantly lower serum insulin levels at 30 minutes and 120 minutes after a oral glucose tolerance test (OGTT), as well as a reduced insulin AUC (Figure 6, D and E), despite comparable plasma glucose levels (Supplemental Table 3). The insulin secretion index (δ30) was also significantly lower in mutation carriers (*P* < 0.05) (Figure 6F).

Linear regression analysis further confirmed the BAF60a^V278M^ variant was significantly associated with lower 30-minute serum insulin levels after OGTT (β = –0.10 [95% CI –0.17 to –0.02]; *P* = 0.012) and with reduced insulin AUC (β = –0.09 [95% CI –0.15 to –0.02]; *P* = 0.010). Notably, analysis of public genomic datasets showed that this variant appears to be East Asian specific, with minor allele frequencies of 0.23% in the 1000 Genomes Project (*n* = 2,504) and 0.20% in the Exome Aggregation Consortium (*n* = 60,684).

### Mice carrying BAF60a^V278M^ mutation have impaired insulin secretion and systemic glucose homeostasis.

**Figure 7:**
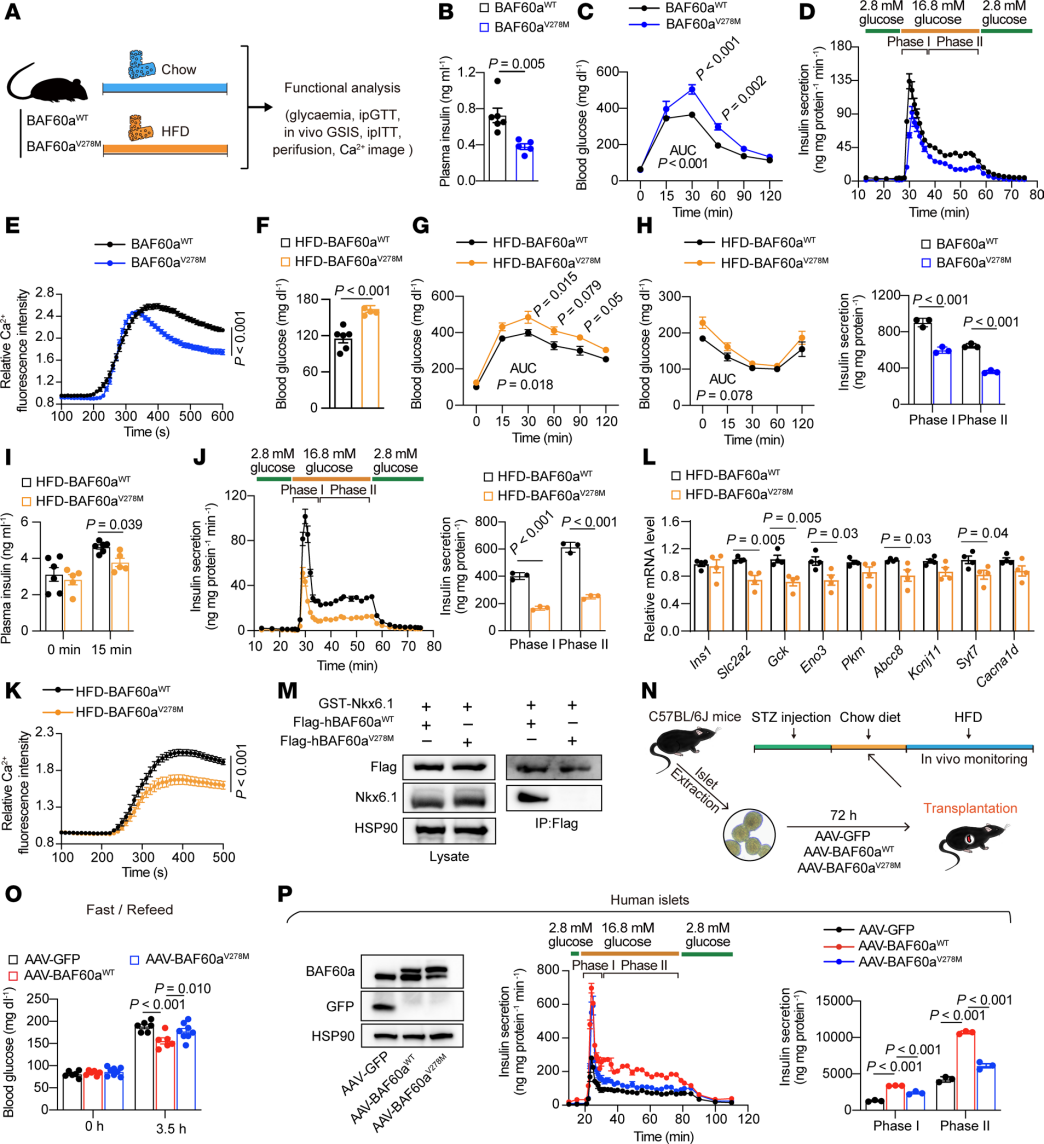
Mice carrying the BAF60a^V278M^ mutation exhibit impaired insulin secretion and glucose homeostasis. (**A**) Experimental design for (**B**–**K**). (**B**) Plasma insulin in chow-fed BAF60a^WT^ and BAF60a^V278M^ mice. Mean ± SEM; *n* = 5–6; 2-tailed unpaired Student’s *t* test. (**C**) ipGTT in chow-fed mice. Mean ± SEM; *n* = 6; 2-way ANOVA. (**D**) Biphasic insulin secretion in islets pooled from 3 mice. Mean ± SD; *n* = 3 technical replicates; 2-tailed unpaired Student’s *t* test. (**E**) Ca²^+^ influx in chow-fed islets pooled from 3 mice. Mean ± SEM; *n* = 60–64; 2-way ANOVA. (**F**) Overnight fasting blood glucose level in HFD-fed mice. Mean ± SEM; *n* = 5–6; 2-tailed unpaired Student’s *t* test. (**G**) ipGTT in HFD-fed mice. Mean ± SEM; *n* = 8–9; 2-way ANOVA. (**H**) ipITT in HFD-fed mice. Mean ± SEM; *n* = 5–6; 2-way ANOVA. (**I**) In vivo GSIS in HFD-fed mice. Mean ± SEM; *n* = 5–6; 2-way ANOVA. (**J**) Biphasic insulin secretion in HFD-fed islets pooled from 3 mice. Mean ± SD; *n* = 3 technical replicates; 2-tailed unpaired Student’s *t* test. (**K**) Ca²^+^ influx in islets pooled from 3 mice. Mean ± SEM; *n* = 45–53; 2-way ANOVA. (**L**) Relative mRNA expression in islets isolated from HFD-fed mice. Mean ± SEM; *n* = 4 biological replicates; 2-tailed unpaired Student’s *t* test. (**M**) Flag IP and immunoblot analysis of BAF60a^WT^/BAF60a^V278M^ and Nkx6.1 interactions in HEK293T cells. (**N** and **O**) Experimental design (**N**), and fasting/refeeding glucose levels (**O**) in STZ-treated mice transplanted with islets transduced with indicated AAVs. Mean ± SEM; *n* = 6–8; 1-way ANOVA. (**P**) Immunoblots (left), perifusion (middle), and quantification of phase I/II insulin secretion (right) in human islets transduced with indicated AAVs. Mean ± SD; *n* = 3 technical replicates; 1-way ANOVA.

To evaluate the physiological relevance of the BAF60a^V278M^ mutation in vivo, we generated C57BL/6J mice harboring this variant using CRISPR/Cas9 and subjected them to a chow diet or HFD feeding for metabolic analysis (Figure 7A). Under chow diet feeding, mutant mice had reduced fasting insulin levels and impaired glucose tolerance (Figure 7, B and C). Perifusion of isolated islets revealed blunted GSIS (Figure 7D), accompanied by reduced glucose-stimulated Ca^2+^ influx (Figure 7E).

Under HFD, BAF60a^V278M^ mice exhibited approximately a 1.4-fold higher fasting glucose level and pronounced defects in glucose tolerance and insulin secretion compared with WT controls (Figure 7, F–I), whereas insulin sensitivity remained comparable (Figure 7H). Perifusion analysis showed reduced phase I (~59.3%; *P* < 0.001) and phase II (~58.2%; *P* < 0.001) insulin release (Figure 7J) with diminished Ca^2+^ response (Figure 7K). Consistently, transcriptome analysis revealed reduced expression of BAF60a- and Nkx6.1-regulated genes important for β cell function (Figure 7L).

Because V278 lies in the predicted Nkx6.1-interaction domain, we tested whether this mutation disrupts binding. Co-IP in HEK-293T cells expressing Flag-BAF60a variants and GST-Nkx6.1 showed that BAF60a^V278M^ abolished interaction with Nkx6.1 (Figure 7M).

To assess the β cell–intrinsic effects of this mutation, we infected mouse islets with AAVs expressing GFP, BAF60a^WT^, or BAF60a^V278M^. Whereas BAF60a^WT^ enhanced GSIS, the V278M variant markedly blunted this effect by 45.2% (phase I) and 41.2% (phase II) (*P* < 0.001) (Supplemental Figure 7). In islet transplantation experiments, BAF60a^WT^-treated mouse islets improved glycemic control in STZ-induced diabetic mice, but this benefit was lost in BAF60a^V278M^-expressing islets (Figure 7, N and O). A similar trend was observed in human islets: while BAF60a^WT^ boosted insulin secretion, co-expression of BAF60a^V278M^ suppressed phase I and II GSIS by 29.4% and 42.8%, respectively, compared with BAF60a^WT^-treated islets (*P* < 0.001) (Figure 7P).

Together, these findings support that the BAF60a^V278M^ variant impairs β cell GSIS and glucose regulation, likely by disrupting BAF60a*-*Nkx6.1 interaction and downstream transcriptional programs.

### BAF60a contributes to the therapeutic benefits of GLP-1R agonist possibly by modulating GLP-1R expression.

Recent studies have shown that GLP-1R and GIPR agonists induce robust insulin secretion and weight loss in T2D (31, 32). Given that Nkx6.1 deficiency impairs *Glp1r* expression (29) and BAF60a function depends on Nkx6.1, we examined whether BAF60a modulates the expression of *Glp1r* and *Gipr* in β cells.

**Figure 8:**
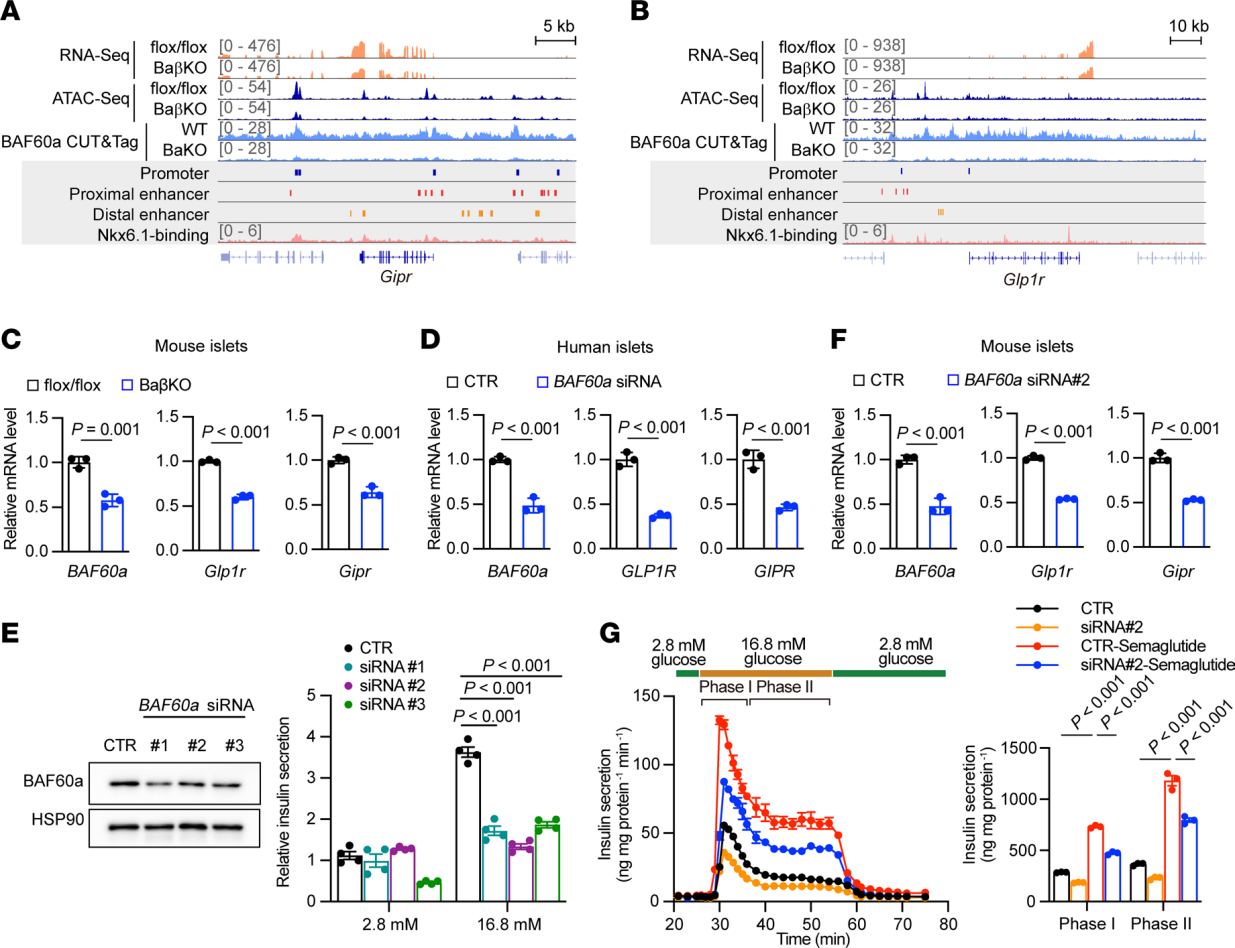
BAF60a contributes to the therapeutic benefits of GLP-1R agonists by modulating GLP-1R expression. (**A** and **B**) Representative RNA-Seq, ATAC-Seq, and CUT&Tag-Seq tracks of *Gipr* (**A**) and *Glp1r* (**B**) loci. RNA-Seq and ATAC-Seq tracks for flox/flox and BaβKO islets. (**C**) Relative mRNA in isolated islets from indicated groups. Mean ± SEM; *n* = 3 replicates; 2-tailed unpaired Student’s *t* test. (**D**) Relative mRNA in human islets transduced with control or *BAF60a* siRNA. Mean ± SEM; *n* = 3 replicates; 2-tailed unpaired Student’s *t* test. (**E**) Immunoblots (left) and GSIS (right) of WT islets transduced with control or *BAF60a* siRNA (1–3). Mean ± SEM; *n* = 4 replicates; 2-way ANOVA. (**F**) Relative mRNA in isolated islets from indicated groups. Mean ± SEM; *n* = 3 replicates; 2-tailed unpaired Student’s *t* test. (**G**) Dynamic (left) and quantification of phase I/II insulin secretion (right) in islets pooled from 3 mice. Mean ± SD; *n* = 3 technical replicates; 2-tailed unpaired Student’s *t* test. Interaction effect: *P* < 0.0001 (phase I) and *P* = 0.0016 (phase II), 2-way ANOVA.

In BaβKO islets, genome browser tracks showed reduced *Glp1r* and *Gipr* expression, accompanied by decreased chromatin accessibility at BAF60a/Nkx6.1-bound enhancers and promoters (Figure 8, A and B). qPCR confirmed reduced *Glp1r* and *Gipr* transcription levels in islets from BaβKO mice (Figure 8C) and in primary mouse and human islets transfected with BAF60a-targeting siRNAs (Figure 8, D–F).

To assess functional consequences, we measured insulin secretion in mouse islets treated with siRNA against BAF60a and stimulated with semaglutide, a GLP-1R agonist. While semaglutide increased phase I and II insulin secretion by approximately 2.6-fold and approximately 3.1-fold in control islets, BAF60a knockdown markedly blunted this response by approximately 35% in both phases (Figure 8G). Two-way ANOVA revealed significant interaction effects between BAF60a knockdown and semaglutide treatment (phase I: *P* < 0.0001; phase II: *P* = 0.0016), indicating a specific defect in GLP-1/GIP responsiveness beyond baseline β cell dysfunction.

Together, these data suggest BAF60a supports the insulinotropic response to incretin therapy, at least in part by maintaining *Glp1r* and *Gipr* expression in β cells.

## Discussion

Islet β cells integrate metabolic and inflammatory cues to modulate insulin secretion and β cell mass in response to systemic insulin demand. Prior studies have reported extensive chromatin remodeling in diabetic human islets (16–18) and rodent models (14), implicating chromatin regulators such as Kat5, Dmap1, Brd8, Hdac1, and the polycomb complex in β cell survival, identity, and insulin secretion (33–37). The SWI/SNF chromatin remodeling complex also supports β cell development and function (38–40), but how upstream metabolic stress signals are selectively integrated to modulate β cell chromatin architecture in T2D remains poorly defined.

Our study extends this understanding by identifying BAF60a, a SWI/SNF subunit, as a key regulator of GSIS through chromatin remodeling in β cells. Mechanistically, BAF60a cooperates with the transcription factor Nkx6.1 to modulate chromatin accessibility at enhancers and promoters of genes critical for β cell function. These findings provide mechanistic insight into how chromatin remodeling selectively governs gene programs impaired in T2D, and distinguish BAF60a as a molecular bridge between SWI/SNF and β cell–specific transcriptional control.

Previous studies have shown tissue-specific roles of BAF60a in liver, adipose, and skeletal muscle, where it partners with PPARα, PGC-1α, and other regulators to control lipid metabolism, thermogenesis, and glycolysis (41–43). Our findings in β cells add to this paradigm, suggesting that BAF60a interacts with distinct transcription factors, here Nkx6.1, to regulate gene expression in a context-dependent manner.

Beyond insulin secretion, β cell identity and survival are also crucial determinants of T2D progression (44–46). Accumulating evidence points to β cell dedifferentiation and trans-differentiation as mechanisms of dysfunction (47–49). Interestingly, our scRNA-Seq analysis also revealed changes in the percentages of hormone-secreting cells in BaβKO islets, suggesting a potential role of BAF60a in controlling β cell identity. However, due to the relatively low sequencing depth of the microwell-based scRNA-Seq approach we used in this study, we did not feel confident in tracking the cell identity changes driven by β cell–specific inactivation of BAF60a by transcriptomic analysis. Also, the current sequencing depth in our study was insufficient to call detailed changes in β cell heterogeneity. Notably, recent work identified CD63hi and CD63low β cell subpopulations with distinct insulin secretion and *Glp1r* expression profiles (50), raising the possibility that BAF60a may influence β cell heterogeneity. Higher-resolution single-cell profiling and lineage-tracing approaches will be essential to address this possibility.

Our ATAC-Seq and BioID analyses also revealed a broad BAF60a interactome in β cells. In addition to Nkx6.1, candidate partners include Foxa2, Isl1, Pdx1, and Mafa, key transcription factors in β cell identity and function (51–53), indicating other potentially important roles of BAF60a in participating in the regulatory processes driven by these transcription factors. Moreover, interactions with SWI/SNF subunits (i.e., Brg1, BAF155, and BAF170) and histone deacetylases (i.e., Hdac2 and Hdac3) suggest BAF60a may coordinate multiple epigenetic regulators. Studies will be needed to dissect these interactions under physiological and stress conditions.

Importantly, we identified a human BAF60a^V278M^ variant significantly associated with impaired GSIS. This variant, which alters a conserved region between the C1 and SWIB domains, was found only in East Asian populations. Mice engineered to carry the BAF60a^V278M^ mutation exhibited glucose intolerance, reduced insulin secretion, and diminished expression of Nkx6.1-target genes, phenocopying BaβKO mice. Mechanistically, this mutation disrupted the physical interaction between BAF60a and Nkx6.1, abrogating the GSIS-promoting effect of BAF60a overexpression in both mouse and human islets. These findings offer a rare example of a naturally occurring human mutation that impairs β cell chromatin regulation and function via disruption of a transcription factor–chromatin remodeler interaction.

Additionally, our research has shown that BAF60a may impact the effectiveness of GLP-1R/GIPR agonists by affecting the expression of *Glp1r* and *Gipr*. We have also demonstrated that BAF60a knockdown in primary mouse islets could effectively reduce the insulinotropic function of semaglutide, a GLP-1R agonist. However, whether a GLP-1R/GIPR dual agonist such as tirzepatide was also affected by BAF60a loss of function remains elusive. In addition, exploring the effectiveness of GLP-1R/GIP agonists in individuals with BAF60a mutations could be another interesting research topic.

In summary, this work defines BAF60a as the key checkpoint factor that links the metabolic stresses to the selective orchestration of chromatin landscape and transcriptional reprogramming of gene networks directing the development of β cell dysfunction in T2D. BAF60a physically interacts with Nkx6.1 to selectively regulate the transcriptional networks important for β cell glucose metabolism and insulin secretion. From the therapeutic perspective, BAF60a is likely to affect the insulinotropic effect of GLP-1R/GIP agonists by modulating the expression of *Glp1r* and *Gipr*. Our results also provide evidence supporting the combination of AAV-mediated gene therapy and islet transplantation for treating diabetes.

## Methods

### Sex as a biological variable.

Sex was not considered a biological variable in this study. Human samples included both male and female donors. Mouse experiments used only male mice to avoid tamoxifen-induced endocrine confounding in females.

### Animal study.

For nonhuman primate studies, pancreas tissue sections from diabetic, obese, and normal female rhesus monkeys were used for immunofluorescence staining, as described in detail in the Supplemental Methods. For mouse studies, we used a conditional BAF60a KO model generated on a C57BL/6J background, crossed with β cell–specific Cre driver strains. Mice were maintained under standard housing conditions and subjected to tamoxifen, an HFD, STZ, or AAV-mediated gene transfer as indicated. In vivo assays, such as glucose tolerance tests (GTTs), insulin tolerance tests (ITTs), and in vivo GSIS, were performed to characterize systemic glucose homeostasis and islet function. Ex vivo assays, such as islet perifusion, AAV induction, ATAC-Seq, and RNA-Seq, were performed on primary islets to characterize islet GSIS function and molecular phenotypes. Full experimental details, including the generation of genetically modified strains, treatment regimens, islet isolation, and details on related ex vivo and in vivo assays, are provided in the Supplemental Methods.

### Cell culture.

HEK-293T/AAV293 and Min6 cells were cultured under standard conditions as described in the Supplemental Methods. CRISPR/Cas9 and shRNA approaches were used to generate BAF60a and Nkx6.1 loss-of-function Min6 cell lines. Cell-based assays, including BioID proximity labeling, co-immunoprecipitation, and CUT&Tag-Seq, were performed to characterize BAF60a function on Min6 GSIS function. Detailed culture conditions, gRNA and shRNA sequences, vector construction, and assay protocols are provided in the Supplemental Methods.

### Statistics.

Statistical analyses were performed using GraphPad Prism 10. Comparisons between 2 groups used a 2-tailed unpaired Student’s *t* test. For more than 2 groups, ANOVA with appropriate post hoc tests was applied. Two-way ANOVA was used for ITT, GTT, GSIS, Ca²^+^ imaging, ATP, and perifusion assays, with FDR-adjusted multiple comparisons. AUC values were compared using a 2-tailed unpaired *t* test. *P* < 0.05 was considered statistically significant. Full statistical parameters and *P* values are provided in figure legends.

### Study approval.

Studies of human samples were approved by the Research Ethics Committee of the First Affiliated Hospital of Zhejiang University (approvals 2017-410-1 and 2018-753-1). Paraffin-embedded human pancreas tissue sections from patients who underwent pancreatectomy due to benign tumors were obtained with the informed consent of the patients. The isolation of human islets was performed with the informed consent of deceased organ donors’ families. All procedures followed ethical guidelines of Zhejiang University School of Medicine and the First Affiliated Hospital. Human population–based studies were approved by the Ethics Committee of Shanghai Sixth People’s Hospital (approvals 2015-27 and 2018-KY-066(K)). Nonhuman primate studies were approved by the Ethics Committee of the Kunming Institute of Zoology and Kunming Primate Research Center, Chinese Academy of Sciences (accredited by the Association for Assessment and Accreditation of Laboratory Animal Care; approval SYDW-20101215-15). All mouse experiments followed the protocols approved by the Animal Ethics Committee of the Second Affiliated Hospital, Zhejiang University School of Medicine (approval 2020-157).

### Data availability.

All RNA-Seq, scRNA-Seq, ATAC-Seq, and CUT&Tag datasets are available in the National Center for Biotechnology Information’s Sequence Read Archive under accession PRJNA1282289 (https://www.ncbi.nlm.nih.gov/sra/PRJNA1282289). Additional data and raw image files are provided in the Supporting Data Values file. All code and packages used are open source and publicly available.

## Author contributions

ZXM conceived the project, designed the research, and supervised the study. ZXM and CH acquired the funding support. RRW, QQW, XQ, HF, SZ, WC, WW, HC, XJ, WZ, MS, PL, QW, ZZ, XP, GG, LZ, DY, YY and HP performed the experiments and the bioinformatic analysis. JY, LJ, RZ, and CH performed the experiments and analyzed the data for human population–related studies; WD and MZ provided human pancreas tissue sections for IHC staining; CL and BL provided monkey pancreas tissue sections for IHC staining; HF, SY, and MZ provided support for human pancreatic islet-related studies; ZXM, XQ, and RRW analyzed and interpreted the data, and wrote the manuscript. Author order among co–first authors was determined by mutual agreement.

## Funding support

National Natural Science Foundation of China grants 82425012, 92457301, 32471170, 91857110, 81722012, 82325010, 82300893, and 82500965.
National Key R&D Program of China grants 2021YFC2701903 and 2018YFA0800403.
Zhejiang Provincial Natural Science Foundation grants LHDMD24H030001, LZ21H070001, and LHDMD22H02001.
Construction Fund of Medical Key Disciplines of Hangzhou grant 2025HZZD06.
Innovative Institute of Basic Medical Sciences of Zhejiang University.
Fundamental Research Funds for the Central Universities.
Shuguang Project grant 21SG11.
Innovative Research Team of High-level Local Universities in Shanghai grant SHSMU-ZDCX20212700.
Major Natural Science Project of the Scientific Research and Innovation Plan of Shanghai Municipal Commission of Education grant 2023ZKZD17.
Shanghai Research Center for Endocrine and Metabolic Diseases grant 2022ZZ01002.
Noncommunicable Chronic Diseases-National Science and Technology Major Project grant 2023ZD0507000.
Zhejiang Provincial Postdoctoral Science Foundation grant ZJ2022117.
China Postdoctoral Science Foundation grant 2022M723284.
Zhejiang University School of Basic Medical Sciences-Affiliated Huzhou Central Hospital Pre-Research Fund grant YYJJ202203.

## Supplementary Material

Supplemental data

Unedited blot and gel images

Supporting data values

## Version 1 10/06/2025

In-Press Preview

## Version 2 12/01/2025

Electronic publication

## References

[1] Franks PW, McCarthy MI (2016). Exposing the exposures responsible for type 2 diabetes and obesity. Science.

[2] Roglic G (2016). WHO Global report on diabetes: A summary. Int J Noncummun Dis.

[3] Association AD (2020). 2. Classification and diagnosis of diabetes: *Standards of Medical Care in Diabetes-2021*. Diabetes Care.

[4] Gaulton KJ (2015). Genetic fine mapping and genomic annotation defines causal mechanisms at type 2 diabetes susceptibility loci. Nat Genet.

[5] Mahajan A (2018). Fine-mapping type 2 diabetes loci to single-variant resolution using high-density imputation and islet-specific epigenome maps. Nat Genet.

[6] DeForest N, Majithia AR (2022). Genetics of type 2 diabetes: implications from large-scale studies. Curr Diab Rep.

[7] Feil R, Fraga MF (2012). Epigenetics and the environment: emerging patterns and implications. Nat Rev Genet.

[8] Keating ST, El-Osta A (2015). Epigenetics and metabolism. Circ Res.

[9] Wang RR (2018). The SWI/SNF chromatin-remodeling factors BAF60a, b, and c in nutrient signaling and metabolic control. Protein Cell.

[10] Ling C (2022). Epigenetics of type 2 diabetes mellitus and weight change - a tool for precision medicine?. Nat Rev Endocrinol.

[11] Krentz NAJ, Gloyn AL (2020). Insights into pancreatic islet cell dysfunction from type 2 diabetes mellitus genetics. Nat Rev Endocrinol.

[12] Al-Mrabeh A (2020). 2-year remission of type 2 diabetes and pancreas morphology: a post-hoc analysis of the DiRECT open-label, cluster-randomised trial. Lancet Diabetes Endocrinol.

[13] Taylor R (2018). Remission of human type 2 diabetes requires decrease in liver and pancreas fat content but is dependent upon capacity for beta cell recovery. Cell Metab.

[14] Wang RR (2022). Dietary intervention preserves β cell function in mice through CTCF-mediated transcriptional reprogramming. J Exp Med.

[15] Kim H, Kulkarni RN (2020). Epigenetics in β-cell adaptation and type 2 diabetes. Curr Opin Pharmacol.

[16] Rai V (2020). Single-cell ATAC-Seq in human pancreatic islets and deep learning upscaling of rare cells reveals cell-specific type 2 diabetes regulatory signatures. Mol Metab.

[17] Chiou J (2021). Single-cell chromatin accessibility identifies pancreatic islet cell type- and state-specific regulatory programs of diabetes risk. Nat Genet.

[18] Khetan S (2018). Type 2 diabetes-associated genetic variants regulate chromatin accessibility in human islets. Diabetes.

[19] Clapier CR (2017). Mechanisms of action and regulation of ATP-dependent chromatin-remodelling complexes. Nat Rev Mol Cell Biol.

[20] Preissl S (2022). Characterizing cis-regulatory elements using single-cell epigenomics. Nat Rev Genet.

[21] Bysani M (2019). ATAC-seq reveals alterations in open chromatin in pancreatic islets from subjects with type 2 diabetes. Sci Rep.

[22] Ho L, Crabtree GR (2010). Chromatin remodelling during development. Nature.

[23] Xiao X (2014). M2 macrophages promote beta-cell proliferation by up-regulation of SMAD7. Proc Natl Acad Sci U S A.

[24] Shapiro AJ (2017). Clinical pancreatic islet transplantation. Nat Rev Endocrinol.

[25] Bruni A (2018). Ferroptosis-inducing agents compromise in vitro human islet viability and function. Cell Death Dis.

[26] He S (2020). Structure of nucleosome-bound human BAF complex. Science.

[27] Ediger BN (2017). LIM domain-binding 1 maintains the terminally differentiated state of pancreatic β cells. J Clin Invest.

[28] Tennant BR (2013). Identification and analysis of murine pancreatic islet enhancers. Diabetologia.

[29] Taylor Brandon L (2013). Nkx6.1 is essential for maintaining the functional state of pancreatic beta cells. Cell Rep.

[30] Roux KJ (2012). A promiscuous biotin ligase fusion protein identifies proximal and interacting proteins in mammalian cells. J Cell Biol.

[31] Campbell JE (2023). GIPR/GLP-1R dual agonist therapies for diabetes and weight loss-chemistry, physiology, and clinical applications. Cell Metab.

[32] El K (2023). The incretin co-agonist tirzepatide requires GIPR for hormone secretion from human islets. Nat Metab.

[33] Wei Z (2018). Vitamin D switches BAF complexes to protect β cells. Cell.

[34] Christensen DP (2011). Histone deacetylase (HDAC) inhibition as a novel treatment for diabetes mellitus. Mol Med.

[35] Fang Z (2019). Single-cell heterogeneity analysis and CRISPR screen identify key β-cell-specific disease genes. Cell Rep.

[36] Lundh M (2012). Histone deacetylases 1 and 3 but not 2 mediate cytokine-induced beta cell apoptosis in INS-1 cells and dispersed primary islets from rats and are differentially regulated in the islets of type 1 diabetic children. Diabetologia.

[37] Lu TT-H (2018). The polycomb-dependent epigenome controls β cell dysfunction, dedifferentiation, and diabetes. Cell Metab.

[38] McKenna B (2015). Dynamic recruitment of functionally distinct Swi/Snf chromatin remodeling complexes modulates Pdx1 activity in islet β cells. Cell Rep.

[39] Spaeth JM (2019). The Pdx1-bound Swi/Snf chromatin remodeling complex regulates pancreatic progenitor cell proliferation and mature islet β-cell function. Diabetes.

[40] Celen C (2022). Arid1a loss potentiates pancreatic β-cell regeneration through activation of EGF signaling. Cell Rep.

[41] Meng ZX (2015). A diet-sensitive BAF60a-mediated pathway links hepatic bile acid metabolism to cholesterol absorption and atherosclerosis. Cell Rep.

[42] Liu T (2020). BAF60a deficiency uncouples chromatin accessibility and cold sensitivity from white fat browning. Nat Commun.

[43] Meng Z-X (2017). Uncoupling exercise bioenergetics from systemic metabolic homeostasis by conditional inactivation of Baf60 in skeletal muscle. Diabetes.

[44] Dhawan S (2011). Pancreatic β cell identity is maintained by DNA methylation-mediated repression of Arx. Dev Cell.

[45] Dor Y (2004). Adult pancreatic beta-cells are formed by self-duplication rather than stem-cell differentiation. Nature.

[46] Hunter CS, Stein RW (2017). Evidence for loss in identity, de-differentiation, and *trans*-differentiation of Islet β-cells in type 2 diabetes. Front Genet.

[47] Cinti F (2016). Evidence of β-cell dedifferentiation in human type 2 diabetes. J Clin Endocrinol Metab.

[48] Spijker HS (2015). Loss of β-cell identity occurs in type 2 diabetes and is associated with islet amyloid deposits. Diabetes.

[49] Kim-Muller JY (2016). Aldehyde dehydrogenase 1a3 defines a subset of failing pancreatic β cells in diabetic mice. Nat Commun.

[50] Rubio-Navarro A (2023). A beta cell subset with enhanced insulin secretion and glucose metabolism is reduced in type 2 diabetes. Nat Cell Biol.

[51] Gao T (2014). Pdx1 maintains β cell identity and function by repressing an α cell program. Cell Metab.

[52] Gao N (2010). Foxa1 and Foxa2 maintain the metabolic and secretory features of the mature beta-cell. Mol Endocrinol.

[53] Ediger BN (2014). Islet-1 Is essential for pancreatic β-cell function. Diabetes.

