# Supplementary material for: BAF60a-dependent chromatin remodeling preserves β cell function and contributes to the therapeutic benefits of GLP-1R agonists
Source: J Clin Invest. 2025 Oct 2;135(23):e177980. doi: 10.1172/JCI177980 (PMC12646663; doi:10.1172/JCI177980)
Supplement: Supplemental data [file jci-135-177980-s312.pdf]

Supplemental Methods

Human studies

To assess the expression of BAF60a in human pancreatic islet β-cells, paraffin-embedded human pancreas tissue sections from patients underwent pancreatectomy due to benign tumors were obtained from the First Affiliated Hospital of Zhejiang University. Detailed information on the patients is provided in Supplemental Figure S1G. Informed consent for the collection, islet isolation, and research of human pancreas tissues from deceased organ donors was obtained from the donor family after the cardiac death of the donors. All the research protocols used in this study strictly complied with all relevant legal and ethical regulations of Zhejiang University School of Medicine and the First Affiliated Hospital of Zhejiang University.

The human population studies were approved by Shanghai Sixth People's Hospital Affiliated to Shanghai Jiao Tong University School of Medicine. Subjects were recruited from a community-based cohort consisting of 13,903 subjects. The characteristics of the subjects are shown in the Supplemental Table S3. We genotyped the p. V278M (c.832G>A [rs200921207]) variant of BAF60a (gene symbol: *SMARCD1*) by four platforms, including 2120 individuals with Infinium Exome-24 v1.0 BeadChip, 4565 individuals with Infinium Multi-Ethnic Global BeadChip, and 6384 individuals with Infinium Asian Screening Array Optimized for East Asian populations (Illumina, Inc., San Diego, CA, USA). TaqMan assay via a 7900HT Fast Real-Time PCR System (Applied Biosystems, Foster City, CA) was used as a supplementary method to genotype the missing alleles mentioned above (a total of 834 individuals). The genotyping of the

variant passed quality control (Call rate>99%, concordant rate＞99%, and consistent with the Hardy-Weinberg equilibrium, *P* value =1). The statistical analysis was performed by SAS (version 8.0; SAS Institute, Cary, NC). Descriptive statistics were calculated for both BAF60a^{WT} and BAF60a^{V278M} (BAF60a mut) groups. Quantitative traits with a skewed distribution were logarithmically transformed and analyzed by linear regression adjusted for age and gender using PLINK (http://pngu.mgh.harvard.edu/ ~ purcell/plink/). A two-tailed *P* value of <0.05 was considered statistically significant.

Monkey studies

To determine the expression of BAF60a in monkey pancreatic islet β-cells, paraffin-embedded pancreas tissue sections from three spontaneous diabetic female rhesus monkeys (*Macaca mulatta*), three matched spontaneous obese, and three normal female rhesus monkeys with similar age (12-18 yr) were used for immunofluorescent (IF) staining. These monkeys demonstrated similar obese and diabetic phenotypes as human patients. Detailed information about the nine monkeys has been described previously (1). Monkeys were maintained under a 12/12 h light/dark cycle and were allowed ad libitum access to water and food. All animal procedures were performed in strict accordance with the guidelines by the National Care and Use of Animals approved by the National Animal Research Authority (People’s Republic of China) and the Institutional Animal Care and Use Committee of the Kunming Institute of Zoology of the Chinese Academy of Sciences.

Mouse studies

BAF60a flox/flox mice on the C57BL/6J background were generated as previously described (2). To achieve deletion of BAF60a specifically in mature β-cells, the MIP-CreERT mice (JAX stock #024709) expressing a tamoxifen-inducible Cre recombinase driven by the mouse insulin I (Ins1) promoter were used to cross with BAF60a flox/flox mice and LSL-BAF60a-Rosa26KI mice, respectively. This mouse strain has been shown to express inducible Cre recombinase specifically in the pancreatic β-cells but not in other tissues or cells such as the brain, pancreatic α-cell, liver, fat, and skeletal muscle (3), and has been widely used in previous studies (4, 5). In some experiments, RIP-Cre mice (JAX stock #003573) and Pdx1-Cre mice (JAX stock #014647) were also used to further confirm the effects of BAF60a inactivation or gain-of-function on β-cell biology and function. Mice were maintained in 12/12 h light/dark cycles, and fed with standard rodent chow or high-fat diet (D12492, Research Diets).

Streptozotocin (STZ) treatment was performed as previously described (6). Briefly, STZ (Cayman Chemical, 13014, USA) was freshly prepared in sodium citric buffer (pH 4.2) and administered to mice through intraperitoneal (IP) injection at 50 mg/kg body weight for five consecutive days.

For tamoxifen treatment, age-matched adult mice (8-12 weeks of age) carrying BAF60a flox/flox: MIP-CreERT and BAF60a flox/flox alone (control) and LSL-BAF60a-Rosa26KI: MIP-CreERT, and LSL-BAF60a-Rosa26KI or MIP1-CreERT alone (control) were subjected to tamoxifen injection. Tamoxifen (Cayman Chemical, 10540-29-1, USA)

was dissolved in corn oil (Sigma, C8267) at 20 mg/ml and administered to mice through IP injection at 100 mg/kg body weight for five consecutive days.

Pancreatic intraductal infusion of the AAV virus was performed as described previously (7). In brief, animals were anesthetized using isoflurane. Purified AAV2/9 viruses expressing GFP control or BAF60a were diluted to $4x10^{12}$ viral genomes per ml (VG/ml) with saline. A total volume of 150 μL viral solution was delivered into the mouse pancreas through an intraductal infusion catheter (World Precision Instruments, CMF31G) at 10 μL/min. Animals were kept warm using a heating blanket for recovery after the procedure.

GTT and ITT

GTT and ITT were performed as previously described (8). For GTT, mice were fasted overnight (~16 h) and intraperitoneally (IP) injected with glucose saline solution (1.2 g/kg body weight, glucose concentrations were adjusted accordingly to obtain equal injection volume for each mouse). Blood glucose levels were measured by tail-snip blood sampling pre-injection and 15-, 30-, 60-, and 120-min post-injection. For ITT, mice were fasted for 4 h and IP injected with insulin saline solution (1 U/kg body weight, concentrations were adjusted accordingly to equalize injection volume for each mouse). Blood glucose levels were measured by tail-snip blood sampling pre-injection and 15-, 30-, 60-, and 120-min post-injection via medical glucometers (Contour, Bayer or Safe AQ Smart, Sinocare).

In vivo GSIS

Chow diet or HFD-fed mice were fasted overnight (~16 h) and IP injected with a glucose solution (30% prepared in saline) at 2 g/kg or 0.8 g/kg body weight, respectively. Blood glucose was monitored at the indicated time points. Whole blood was collected at different time points from tails into tubes containing 1 μL of 0.5 M EDTA, followed by centrifugation at 8,000 rpm, 4°C for 8 min to obtain plasma samples. Plasma insulin levels were measured using a mouse insulin ELISA kit (Crystal Chem, Cat# 90080).

Murine primary islet isolation

Mouse islets were isolated from the pancreas of 2% pentobarbital sodium (Sigma, P3761) sedated mice by intraductal collagenase (Collagenase type V, 1 mg/mL, Sigma, C9263) digestion and purified by Histopaque 1077 (Sigma, 10771) density gradient centrifugation following a previously described protocol (9). Purified islets were then hand-picked under a stereomicroscope. Isolated islets were incubated overnight in RPMI-1640 medium (Gibco, 11875-093) containing 50 μg/mL streptomycin, 50 U/mL penicillin, and 10% FBS in a humidified incubator at 37°C with 5% CO_2.

Islet perifusion assay

Primary murine islets were cultured overnight post-isolation before perifusion assays. For each assay, perifusion chambers were loaded with 150 IEQ islets. Islets were first perifused with Kreb's buffer (135 mM NaCl, 3.6 mM KCl, 0.5 mM NaH_2PO_4, 1.5 mM $CaCl_2$, 2 mM $NaHCO_3$,10 mM HEPES, and 0.1 % BSA, pH 7.4) containing 2.8 mM glucose for 30 min, followed by perifusion with Kreb's buffers containing 2.8 mM

glucose, 16.8 mM glucose and 2.8 mM glucose for 25, 30 and 20 minutes, respectively, under 1 mL/min flow rate. Fractions were collected every minute. Islets were collected from the perifusion chamber after assay to obtain total protein content via BCA protein assay. Insulin secretion at each time point was quantified using an HTRF insulin assay kit (Cisbio, 62INSPEC) according to the manufacturer's instructions and normalized to total protein content. To quantify the biphasic insulin secretion, phase I and phase II of insulin secretion were manually separated based on the plateau formation of the II phase and quantified by calculating the Area under curve (AUC). Insulin levels were normalized to the average baseline insulin secretion in a buffer containing low glucose (2.8 mM) and presented as fold insulin secretion.

siRNA-mediated BAF60a knockdown in primary islets

Primary islets (300 IEQ) isolated from wild-type C57BL6/J mice were transiently transfected with 100 nM of Control siRNA or *BAF60a*-targeting siRNA (#1: 5'-GAAACGUCCCAUCAAGCAA-3'; #2: 5'-CAGCGAGAGUUCAUGUUGA-3'; #3: 5'-GAGUUUGUUCUCUGUGACA-3') using Lipofectamine RNAiMAX Reagent (Thermo Fisher Scientific) according to the manufacturer's instruction. Islets were harvested at 48 h post- transfection for either Western blotting analysis of BAF60a protein levels or dynamic GSIS function study. BAF60a knockdown in human islets were conducted following a similar procedure with the siRNAs (5'-GAUCCAUGAGACAAUAGAA-3').

Transmission electron microscopy and quantification

Mouse islets were fixed in 2.5% glutaraldehyde in PBS overnight at 4℃. After being washed 3 times (15 min each) with 0.1 M phosphate buffer, islets were fixed in 2% aqueous osmium tetroxide for 1 h at room temperature, followed by washing 3 times (15 min each) with ddH_2O. Samples were dyed with 2% uranyl acetate for 30 min, and dehydrated through a graded series of ethanol. Samples were then infiltrated and embedded in Embed-812 resin and cured for 24 h at 37℃, 45℃, and 60℃, respectively. Ultrathin (70 nm) sections were stained with 2% uranyl acetate and 0.3% lead citrate. Transmission electron microscopy (TEM) images were acquired using a FEI Tecnai G2 Spirit Transmission Electron Microscope at the Center of Cryo-Electron Microscopy (CCEM), Zhejiang University, and analyzed with ImageJ 1.52q software (NIH).

Hematoxylin-eosin staining of murine pancreas tissue and quantification

Freshly dissected murine pancreas tissues were fixed in 4% paraformaldehyde (Sigma, P6148) for 16 h at 4°C, embedded with paraffin, and stained with hematoxylin and eosin (H&E). H&E slides were scanned using the Aperio ScanScope XT scanner (Leica). Islet number and area were quantified by ImageScope software (Leica v12.3.3.5048). At least 4 biological replicates were used in each group. For each biological replicate, four randomly selected sections.

Immunofluorescence of murine pancreas tissue and quantification

Freshly dissected murine pancreas tissues were fixed in 4% paraformaldehyde for 16 h at 4°C, embedded in paraffin, and sliced into sections with 4 μm thickness. Tissue sections were deparaffinized in xylene and rehydrated through graded concentrations of ethanol

(100%, 95%, 70%), followed by a ddH_2O rinse. Sections were then soaked in sodium citrate (Sangon Biotech, E673001) and high-pressure cooked for 10 min to retrieve antigen activity. After cooling to room temperature, sections were washed with PBS and blocked with 10% BSA (Yeasen, 36106ES25) in PBS for 1 h at room temperature before incubating with primary antibodies in PBS containing 5% BSA overnight at 4°C. The next day, slides were washed with PBS three times and then incubated with Alexa Fluor-conjugated secondary antibodies (Invitrogen; dilution 1:200) at room temperature for 1.5 h. Nuclei were counterstained with DAPI (Invitrogen, P3693l; dilution 1:2,000). Sections were then mounted with Fluoromount-G™ mounting medium (Yeasen, 36307ES08) and sealed with nail polish. Images were acquired using ZEISS LSM 800. The cell number and area were quantified using ImageJ 1.52q software (NIH). At least four biological replicates were used in each group. Each biological replicate included six or more islets. Data visualization follows the superplot approach and statistical tests described in (10).The following first antibodies were used: Insulin (homemade; dilution 1:10,000), Glucagon (Sigma, G2654; dilution 1:2,000) and BAF60a (Sigma, HPA004101; dilution 1:50), Slc2a2 (Proteintech, 20436-1-AP; dilution 1:50), APC-CD45 (Biolegend, 103112; dilution 1:100), and APC-F4/80 (Biolegend, 123116; dilution 1:100).

BAF60a mutagenesis

Adeno-associated virus (AAV) vectors encoding Flag/HA-tagged mouse and human BAF60a (pAAV-CAG-FH-mBAF60a and pAAV-CAG-FH-hBAF60a) were used as the templates for mutagenesis. Mouse and human BAF60a^{V278M} mutations were generated with the standard overlap-extension PCR using the primers listed below. For pAAV-

CAG-FH-hBAF60a^{V278M}, $F_{hBAF60a}$: 5'-cggctagatatccaagaggccttg-3', $R_{hBAF60a}$: 5'-gagactcaaagatctgctgcaggtac-3', $F_{hBAF60a\_mut}$: 5'-gcggccgggagacAtgaatgtacgg-3', $R_{hBAF60a\_mut}$: 5'-ccgtacattcaTgtctcccggccg-3'. For pAAV-CAG-FH-mBAF60a^{V278M}, $F_{mBAF60a}$: 5'-cggctagatatccaggaggccttg-3', $R_{mBAF60a}$: 5'-gagattcaaagatctgctggaggtac-3', $F_{mBAF60a\_mut}$: 5'-gcggccaggagatAtgaatgtacgg-3', $R_{mBAF60a\_mut}$: 5'-cgtacattcaTatctcctggccg-3'. Mutation sites were capitalized. Original vectors and overlap-extension PCR products containing the V278M mutation were digested with Bgl II and EcoR V (NEB) and ligated with T4 DNA ligase (Takara). All the vectors were further confirmed by sequencing.

BioID and mass spectrometry

The proximity-labeling BioID approach, as previously described (11), was used to identify proteins that interact with BAF60a. A coding sequence expressing BAF60a and BirA* biotin ligase fusion protein (BAF60a-BirA*) was cloned into the retroviral MSCV-puro vector. Min6 cells were transduced with retroviruses expressing BAF60a-BirA* and subjected to puromycin selection. Cells were incubated for 24 h in complete media supplemented with or without 20 μM biotin. After three washes with PBS, cells were lysed in lysis buffer containing 50 mM Tris-Cl, 137 mM NaCl, 1 mM EDTA, 1% Triton X-100, 10% Glycerol, 10 mM NaF, 10 mM $Na_4P_2O_7$, 10 mM Na_3VO_4 and protease inhibitor cocktail (Roche, 11836145001). Lysed cells were centrifuged under 14,000 *g* for 10 min at 4°C. Supernatants were then incubated with 20 mL streptavidin agarose beads for 24 h at 4°C. Beads were pelleted by centrifugation at 2,000 rpm for 2 min and eluted with Elute Buffer (1.5 × SDS protein sample buffer containing 1 mM biotin). To identify BAF60a-interacting proteins, protein samples from the above elutions

were subjected to electrospray ionization tandem MS on a Thermo LTQ Orbitrap instrument. To further confirm the results from MS, protein samples were separated by SDS-PAGE gels and transferred to a PVDF membrane (Millipore), followed by immunoblotting with the following primary antibodies: BAF60a (BD Biosciences, 611728; 1:1,000), Nkx6.1 (DSHB, F55A12; 1:500), Brg1 (Santa Cruz, sc-10768; 1:500), BAF155 (Santa Cruz, sc-10756; 1:500), BAF53a (Proteintech group Inc, 10341-1-AP; 1:500), BAF170 (Santa Cruz, sc-10757x; 1:500), HSP90 (CST, C45G5; 1:1000), β-actin (Sigma, A4700; 1:1000), and α-tubulin (Abclonal, A6830, 1:1000).

Immunoprecipitation and western blot

Human and mouse BAF60a^{V278M} mutants were generated by site-directed mutagenesis as previously described (12). Mouse BAF60a, mouse BAF60a^{V278M} mutant, human BAF60a^{V278M} mutant, full length and truncations of human BAF60a were cloned into pcDNA3.0 vector. Nkx6.1 was cloned using the Gateway cloning system (Invitrogen) to generate an N-terminal Myc tag fusion protein. These plasmids were co-transfected into HEK-293T cells. Cells were washed with cold PBS and lysed by lysis buffer (50 mM Tris-HCl (pH 7.5), 137 mM NaCl, 1% Triton X-100, 10 mM NaF, 10 mM $Na4P_2O_7$, 1 mM Na_3VO_4, 1 mM EDTA, 10% Glycerol and protease inhibitor cocktail (Roche, 11836145001)) 48 h post-transfection. Lysed cells were centrifuged at 14,000 g for 10 min. The supernatants were incubated with anti-Flag M2 agarose beads (Sigma, A2220) at 4°C for 4 h. Beads were then washed three times with wash buffer (20 mM Tris-HCl (pH 8.0), 0.2 mM EDTA, 100 mM KCl, 2 mM $MgCl_2$, 0.1% Tween 20, and 10% Glycerol), resuspended in 30-50 μl of 1 × SDS protein sample buffer, and finally

denatured at 98°C for 10 min. Protein extracts were separated by SDS-PAGE gels, and transferred to a polyvinylidene difluoride (PVDF) membrane (Millipore), followed by immunoblotting with the following primary antibodies: Flag (Sigma, A8592; 1:500-1:1,000), Myc (Sigma, C3956; 1:1,000), Nkx6.1 (DSHB, F55A12; 1:500), Brg1 (Santa Cruz, sc-10768; 1:500), BAF155 (Santa Cruz, sc-10756; 1:500), BAF170 (Santa Cruz, sc-10757X; 1:500), BAF53a (Proteintech group Inc, 10341-1-AP; 1:500), BAF60a (BD Biosciences, 611728; 1:1,000), BAF60b (Abcam, YCA-R7782-56-2M-1; 1:1,000), Pdx1 (CST, 5679S; 1:1,000), GFP (CST, 2956; 1:1,000), HSP90 (CST, C45G5; 1:1,000), and β-actin (Sigma, A4700; 1:1,000).

GST pull-down assay

Plasmids encoding GST-tagged BAF60a and BAF60a truncations were generated by subcloning BAF60a or truncated BAF60a into pGEX-5X1 vector using standard cloning techniques as described previously (13). Nkx6.1 was cloned into a Gateway destination vector to generate N-terminal His-tagged protein. BL21 (DE3) *Escherichia coli* competent cells were used to transform these constructs. Cells were grown in TB liquid medium to OD 600 of approximately 0.8 and then induced with 0.5 mM isopropyl-β-D-thiogalactoside (IPTG) overnight at 180 rpm at 16°C. For GST-BAF60a protein, cells were lysed by sonication in lysis buffer (500 mM NaCl,1 mM EDTA, 0.5% Triton-X100, 20 mM Tris-HCl, pH 7.5, protease inhibitor cocktail from Roche) and centrifuged at 12,000 rpm for 15 min. GST-Sepharose resin (GE Healthcare) pre-washed with GST wash buffer (150 mM NaCl, 1 mM EDTA, 0.5% NP40, 20 mM Tris-HCl, pH 7.5, protease inhibitor cocktail from Roche) was added to the supernatant and rotated at 4°C

for 2 h. His-Nkx6.1 proteins were purified by Ni-NTA agarose affinity chromatography, according to the manual of QIA expressionist (QIAGEN). Resin bound GST fusion proteins were washed in in vitro binding buffer (150 mM NaCl, 20 mM Tris, 1 mM EDTA, 0.5% NP40, pH 7.5) and incubated with purified His-Nkx6.1 protein for 4 h by gentle rotation at 4℃. After washing with binding buffer for three times, bound proteins were eluted from resin using protein loading buffer, resolved by SDS-PAGE gels, and transferred to PVDF membrane. Nkx6.1 was detected by western blotting with anti-Nkx6.1 (DSHB, F55A12; 1:500) and anti-His (Sigma, H1029; 1:1,000-3,000) antibodies.

Cell culture

HEK-293T or AAV293 cells were cultured in DMEM (Gibco,11995065, Thermo Fisher Scientific, USA) supplemented with 10 % (vol./vol.) fetal bovine serum (FBS, SE100-011, VisTech, New Zealand), and 50 μg/ml streptomycin, 50 U/ml penicillin (Gibco, 15140122, Thermo Fisher Scientific, USA). Min6 cells were grown in DMEM (Gibco, high glucose) containing 15% ES-FBS (SE200-ES, VisTech, New Zealand), 50 μM 2-β mercaptoethanol (Sigma-Aldrich, M3148, USA), 50 μg/ml streptomycin, 50 U/ml penicillin. All cells were cultured in a humidified 5 % CO_2 incubator at 37℃.

Min6 BAF60a or Nkx6.1 knockout cell lines were generated using the lentiCRISPR v2 system from Dr. Feng Zhang's lab (MIT). The gRNAs targeting BAF60a and Nkx6.1 were designed using the Benchling software (https://www.benchling.com/crispr/). Two gRNAs targeting BAF60a (#1: 5’-gccgggcccattcgcacggg-3’ and #2: 5’-tggtctggcccagtcaggga-3’) were selected. Three gRNAs were chosen for Nkx6.1 knockout

(#1: 5’-gttatgtgagcccaaaggtg-3’, #2: 5’-gagccggccctctatgccgg-3’ and #3: 5’-tttcgaacaaacgaagtact-3’). These gRNAs were cloned into lentiCRISPR v2 vector for lentivirus production and transduction.

Min6 BAF60a knock-down stable cell lines were generated as previously described(6). Briefly, pSuper-Retro-Puro vector was digested with restriction enzymes Bgl II and Hind III (NEB), and purified with a PCR purification kit (Invitrogen). DNA oligos carrying BAF60a targeting shRNAs were synthesized, annealed, and ligated into the purified pSuper-Retro-Puro vector with T4 DNA ligase (Takara). Two independent BAF60a targeting shRNA sequences (#1: 5’-gtagccgaatgacacctca-3’; #2: 5’-gcgagagttcatgttgagctt-3’) were selected.

Lentivirus and retrovirus were prepared as previously described (8). For lentiviral production, 1 μg of lentiCRISPR v2 shuttle vector together with two packaging vectors (1 μg of pMD2G and 2 ug of psPAX2) were transiently transfected into HEK-293T cells grown in a 35 mm dish at 80-90% confluency using PEI. For retroviral production, retroviral shuttle vector (MSCV-Puro-FH-BAF60a, pSuper-Retro-Puro-BAF60a shRNA#1, or shRNA#2; 1.2 μg per 35 mm dish) together with two packaging vectors (pVSV-G and pGag-Pol, 1 μg each per 35 mm dish) were transiently transfected into HEK-293T cells using PEI.

AAV preparation

AAV production and purification were performed by ChuangRui Bio (Lianyungang,

China). In brief, AAV293 cells (Agilent, 240073) were cultured at 37°C in a humidified incubator with 5% CO_2. Cells were cultured in Dulbecco's Modified Eagle Medium (Gibco, 11995065) with 10% (v/v) fetal bovine serum (FBS) (Vistech, SE100-011). One day before transfection, AAV293 cells were seeded on twenty 15 cm culture dishes and grown to approximately 90% confluency before transfection. For each 15 cm dish, 7 μg of AAV shuttle vector carrying target genes, 20 μg of Delta F6 helper vector (Addgene, 112867), 7 μg of RC2/9 vector (Addgene, 112865), and 250 μL of PEI (Polysciences, 23966-2) were added to 1 mL DMEM, incubated at room temperature for 15 min, and supplemented into cell cultures. Culture medium was replaced with DMEM containing 0.5% (v/v) FBS at 24 h post-transfection. Cells were collected at 72 h post-transfection and resuspended in 5 mL cell lysis buffer (150 mM NaCl, 20 mM Tris pH 8.0) after washing with 20 mL PBS solution. AAVs were purified via discontinuous iodixanol gradient (Sigma, D1556-250ML). In brief, cells in lysis buffer were freeze-thawed three times, followed by adding 1 M $MgCl_2$ and 25 KU/mL Benzonase (Sigma, E8263-25k) solutions to the final concentrations of 1 mM and 250 U/mL, respectively. Cell lysates were incubated at 37°C for 15 min and centrifuged at 4,000 rpm for 30 min under 4°C. Iodixanol solutions were added into the ultracentrifuge tube (Beckman, 361625) in the order of 60%, 40%, 25%, and 17%. Cell lysates were transferred onto the top layer gently, and the remaining volume of the ultracentrifuge tube was filled up with cell lysis buffer. The virus was then isolated from the 40% layer after centrifugation at 53,000 rpm (Beckman OptimaX100 Ultracentrifuge with Type 70Ti rotor) at 14°C for 2 h 40 min. Viral titer was determined by qPCR assay with the standard curve generated by serial dilutions of the AAV shuttle vector.

GSIS assay of Min6 cells

Min6 cells were preincubated for 30 min in glucose-free Kreb's buffer, followed by incubation in Kreb's buffer containing 2.8 mM and 16.8 mM glucose for 1 h. The supernatant fractions were obtained after static incubation for insulin measurement. Cells were lysed using cold lysis buffer to obtain total protein content via BCA protein assay. Insulin secretion at each condition was quantified using an HTRF insulin assay kit (Cisbio, 62INSPEC) according to the manufacturer's instructions and normalized to total protein content.

Intracellular calcium imaging of primary islets and Min6 cells

Primary murine islets cultured overnight post-isolation were washed twice with Kreb's buffer containing 2 mM glucose, loaded with Kreb's buffer containing 5.6 µM Fluo-4 AM (Thermo Fisher, F14201), 0.4‰ Pluronic F127 (Sigma, P2443), and 2 mM glucose for 45 min. Islets were then washed twice with Kreb's buffer containing 2 mM glucose, seeded on 35 mm glass-bottom dish (MatTek), and cultured for another 15 min. Islets were imaged in a humidified 37°C cell culture chamber with 5% CO_2 mounted on a Zeiss LSM 800 inverted confocal microscope. Continuous image acquisition with 10-second intervals started 2 minutes after glucose supplementation to reach a 16.8 mM final concentration until fluorescent plateaus were reached. Fluorescent intensity dynamics were calculated via ImageJ 1.52q software (NIH).

Min6 cells were seeded and allowed to attach to cover slides in 35 mm Glass-Bottom dishes. Cells were washed twice with KRBH buffer plus 2 mM glucose and loaded with the calcium indicator Fluo-4 AM in KRBH buffer containing 2.8 µM Fluo-4 AM, 0.4‰ Pluronic F127, and 2 mM glucose for 20 min. Cells were then washed twice with KRBH buffer plus 2 mM glucose and incubated in KRBH buffer plus 2 mM glucose for 20 min. Cells were placed into the humidified 37°C culture chamber with 5% CO_2 and mounted on a Zeiss LSM 800 confocal microscope as described above. Images were acquired every 2 seconds before and after adding glucose to the final concentration of 16.8 mM and analyzed with ImageJ 1.52q software (NIH).

ATP measurement of Min6 cells

Min6 cells were preincubated for 1 h in glucose-free KRBH buffer, followed by incubation in KRBH buffer containing 2.8 mM or 16.8 mM glucose for 7 min and 30 min. ATP content in cell lysate was measured using a luciferin-luciferase bioluminescent assay kit (Sigma-Aldrich, FLASC-1KT) as previously described (6).

Metabolic flux of Min6 cells

Min6 cells were preincubated for 1 h in glucose-free KRBH buffer, followed by incubation in KRBH buffer containing 16.8 mM U-^{13}C6-glucose (Cambridge Isotope Laboratories, Inc. CLM-1396-1) for 30 min. The supernatant fractions were obtained after the static incubation. The insulin levels were measured using a mouse insulin ELISA kit as quality control. Cells were quickly washed with 5% D-mannitol (Sigma-Aldrich, M4125) in ddH_2O at room temperature, and immediately quenched in liquid

nitrogen before metabolite extraction. To extract hydrophilic metabolites, cells were scraped into Eppendorf tubes (Eppendorf, Germany) with 500 mL methanol (Merck, USA), followed by vortexing for 20 seconds. Then, 500 μL of chloroform (Duksan Pure Chemicals, South Korea) and 200 μL of ddH_2O were added into each tube sequentially, followed by vortexing for 20 seconds after adding each reagent. After centrifugation at 14,000 g, 4℃ for 15 min, the supernatant was ultra-filtrated and lyophilized. Finally, the samples were re-dissolved in 30 μL ddH_2O for metabolomics analysis using capillary electrophoresis time-of-flight mass spectrometry (CE-TOF/MS). CE-TOF/MS analysis was performed on a CE system (G7100A, Agilent, USA) coupled to a TOF/MS system (G6224A, Agilent, USA) as described previously (14). The fused silica capillary (50 μm i.d. × 80 cm) from Human Metabolome Technologies (HMT, Japan) was used for sample separation. The distribution of mass isotopomers and were calculated according to the protocol described by Zeng et al. (15). Metabolite quantification and peak extraction were performed using the standard metabolite library provided by Human Metabolome Technologies (HMT, Japan), ensuring accurate identification and quantification of detected metabolites. Peak extraction and identification were carried out with Quantitative Analysis Software (Agilent, USA). To facilitate peak identification, about 500 authentic metabolite standards were pre-analyzed by CE-TOF/MS. To correct natural isotopes and calculate mass isotopologue distribution (MID), the data were processed by stable isotope-assisted metabolomics analyzer (SIAMA) software as previously reported (15).

Quantitative polymerase chain reaction (qPCR)

TRIzol reagent (Life Technologies) was used to extract total RNA from islets and cultured cells. For each sample, a total of 1 μg of RNA was reverse-transcribed using the HiScript II Q RT SuperMix for qPCR (Vazyme, R222-01). Gene expression analyses were performed using SYBR Green reagent (Roche, S1816). Relative gene expression levels were calculated and normalized to mouse or human ribosomal protein *36B4* as previously described (6). Primers for qPCR analysis are shown in Supplemental Table S2.

RNA-Seq of primary murine islets

Primary murine islets were subjected to RNA isolation with Trizol Reagent (Invitrogen). 200 ng of RNA was sent to library preparation and sequencing in the BGI group (Wuhan, China). In brief, mRNAs were enriched from total RNA and fragmentized, followed by reverse transcription and second-strand cDNA synthesis. The cDNAs were then ligated with sequencing adaptors and amplified for paired-end sequencing. Data were processed following the standard BGI mRNA analysis pipeline. Statistical analysis was performed with *Deseq2* (v.1.20.0) package (16). Statistical parameters to call differentially expressed genes in each analysis were described in the figure legend. Unsupervised clustering and heatmap visualization were performed with *pheatmap* (V1.0.12, https://cran.r-project.org/web/packages/pheatmap/index.html). GO and pathway grouping and enrichment analysis were performed by *clusterProfiler* (V3.12.0) (17). If not mentioned in the figure legends, significantly changed genes were identified by $|\log_2FC| > 0.5$, $FDR < 0.05$ (P-value by Wald test).

ATAC-Seq of primary murine islets

The protocol of ATAC-Seq was adapted from a previous report (18). In brief, 50,000 cells were extracted from isolated mouse islets by TrypLE (Thermo Fisher, 12604013). Cells were lysed with lysis buffer (10 mM Tris-HCl pH 7.4, 10 mM NaCl, 3 mM $MgCl_2$ and 0.1% (v/v) Igepal CA-630) at 4°C for 10 min. Tagmentation and amplification were done following the manufacturer's instruction with TruePrep DNA library Prep Kit V2 (Vazymes, TD501). Paired-end sequencing was performed by Annoroad Gene Technology (Beijing, China), and clean reads with trimmed adapters were aligned to mm10 reference genome with *Bowtie2* (2.3.4.1) package (19).

The downstream analysis pipeline was adapted from the previous study (20). Briefly, Broad peaks were called by *Macs2* (2.1.1.20160309) package (21) using the parameter of (--nomodel --shift -100 --extsize 200 -B --broad), and differentially accessible peaks were called using *Deseq2* (v.1.20.0) package (16). Differentially accessible peaks were thereby called by $|log_2FC| > 0.5$. Motif enrichment was performed by *HOMER* (V4.10) (22) using peaks filtered by corresponding criteria mentioned in the figure legends. Browser tracks were visualized by *IGV browser* (V2.4.14) (23) after normalizing the reads from each individual sample to its own library size. To compare the ATAC-seq signal with BAF60a CUT&Tag data or previously published ChIP-Seq data , metagene analysis was performed with *Deeptools2* (V3.1.2) (24) using ATAC peaks against the BigWig list of CUT&Tag peaks. For optimal visualization of ChIP-Seq peaks around the ATAC peaks, the ATAC peaks plotted are scaled into 800 bp length, the ChIP-Seq peak distribution from the -3 kb to +3 kb region relative to the peak start sites and peak end sites were

analyzed. For transcriptional footprint analysis, Nkx6.1 binding motifs were scanned by *fimo* (V5.0.5) (25) throughout the whole mm10 genome; footprints were then called with *CENTIPEDE* (V1.2) (26) as described previously (20). Ataqv (V1.0.0, https://github.com/ParkerLab/ataqv) package developed by Parker's lab from the University of Michigan was used to perform the ATAC-Seq data quality control analysis. Overrepresentation analysis was performed using a hypergeometric test with the *phyper* package in R. Islet H3K4me1, H3K4me3, and H3K27ac binding signals were obtained from GSE68618. Binding signals of *Pdx1, Foxa2, Isl1, and Nkx6.1* were retrieved from SRA008281, GSE84759 and SRR573711. Peaks were annotated to their nearest gene by the annotatePeaks.pl by *HOMER* (V4.10).

CUT&Tag and analysis

The library preparation for CUT&Tag was performed as previously reported (27) with a CUT&Tag library preparation kit (Vazyme, TD904). In brief, approximately 5×10^4 single cells were washed twice in 1 mL PBS. Cells were incubated with 10 µL of activated concanavalin A-coated magnetic beads in 500 µL Wash Buffer (20 mM HEPES pH 7.5, 150 mM NaCl, 0.5 mM Spermidine, and protease inhibitor cocktail (Roche) for 10 min at room temperature. Cell-bound beads were collected and resuspended with 50 µL Dig-Wash Buffer (20 mM HEPES pH 7.5, 150 mM NaCl, 0.5 mM Spermidine, protease inhibitor cocktail, 0.05% Digitonin) containing 2 mM EDTA, 0.1% BSA and a 1:50 dilution of the primary antibody (Rabbit anti-BAF60a, Sigma, HPA004101), and incubated at 4°C overnight. Beads were then washed with Dig-Wash buffer and incubated with a secondary antibody (Goat anti-Rabbit IgG, Sigma, SAB3700883) diluted at 1:50 in

100 μL of Dig-Wash buffer for 60 min at room temperature. Hyperactive pG-Tn5 was assembled per the manufacturer's instructions and used for tagmentation at 37°C. Spike-in DNA was added immediately afterward. Libraries were PCR-amplified (12 cycles), size-selected with VAHTS DNA Clean Beads (Vazyme N411), and sequenced on an Illumina platform (Annoroad). Reads were trimmed, aligned to mm10 and spike-in references, spike-in-normalized, and peaks called with a similar pipeline used for ATAC-seq. Regions with ≥ 6-fold higher signal in WT versus BAF60a-KO Min6 cells were considered bona-fide BAF60a sites.

Single-cell RNA-Seq of primary murine islets

Single-cell library preparation was conducted following the previously reported protocol (28). In brief, 150-200 mouse islets were isolated and dissociated into single cells (~2.5 × 10^5 cells) by TrypLE (Thermo Fisher, 12604013). Approximately 1 × 10^5/sample of resuspended single cells were loaded onto microwell-containing agarose plates. Barcoded beads were loaded after cell doublets removal. Cells were lysed using cold lysis buffer (0.1 M Tris-HCl pH 7.5, 0.5 M LiCl, 1% SDS, 10 mM EDTA, and 5 mM dithiothreitol). The beads were then collected and washed once with 1 mL of 6 × SSC, once with 500 μL of 6 × SSC, and once with 200 μL of 50 mM Tris-HCl pH 8.0. Samples were then conducted reverse transcription following the Smart-seq2 protocol (29). The obtained cDNAs were treated with exonuclease I (NEB) and amplified using HiFi HotStart Readymix (Kapa Biosystems) with primers used in the previous report (28). Amplified cDNA libraries were then purified with AMPure XP beads (Beckman Coulter), fragmented, and PCR amplified following the instruction of TruePrep DNA Library Prep

Kit V2 for Illumina (Vazyme), replacing the index 2 primers with the primers reported previously (28). PCR products were purified with AMPure XP beads (Beckman Coulter) and were subjected to sequencing on the Illumina HiSeq systems by Annoroad Gene Technology (Beijing, China).

The scRNA-Seq data processing pipeline was adapted from the previous study (28). Briefly, raw reads were filtered and extracted for barcode and unique molecular identifier (UMI) with *bbmap* (https://sourceforge.net/projects/bbmap/). Filtered reads were then mapped to the mm10 genome with *STAR* (V2.5.2a) (30) with default parameters. Unique alignments were then used for UMI counting to generate the digital gene expression (DGE) data matrix. Cells with less than 200 genes detected were excluded. *Seurat* (V3.2.1) (31) package was used for follow-up dimension reduction and clustering. After initial clustering using Principal Component Analysis (PCA) with the top 500 highly variable genes, the expression level of *Ins1, Ins2, Gcg, Sst, and Ppy* in the immune cell cluster (with H2-Ab1 as the major cluster marker) were considered as background signal to eliminate the possible contamination of highly-expressed hormone RNAs. Cells were then reclustered using PCA with the top 500 highly variable genes. Follow-up differential gene expression analysis was performed with *Seurat,* and *ggplot2* (V3.2.1) (32) was used for plotting results.

Microarray

Microarray data of islets from control and *db/db* mice were downloaded from the Gene Expression Omnibus (GEO) database with accession number GSE31953 (33). Data was

processed following a standard microarray analysis pipeline. Differential analysis was performed with *limma* (V3.11) (34) following developers' instructions.

Reference

1. Wang J, Xu S, Gao J, Zhang L, Zhang Z, Yang W, et al. SILAC-based quantitative proteomic analysis of the livers of spontaneous obese and diabetic rhesus monkeys. *Am J Physiol Endocrinol Metab.* 2018;315(2):E294-e306.
2. Meng ZX, Wang L, Chang L, Sun J, Bao J, Li Y, et al. A Diet-Sensitive BAF60a-Mediated Pathway Links Hepatic Bile Acid Metabolism to Cholesterol Absorption and Atherosclerosis. *Cell Rep.* 2015;13(8):1658-69.
3. Wicksteed B, Brissova M, Yan W, Opland DM, Plank JL, Reinert RB, et al. Conditional gene targeting in mouse pancreatic ß-Cells: analysis of ectopic Cre transgene expression in the brain. *Diabetes.* 2010;59(12):3090-8.
4. Shirakawa J, Fernandez M, Takatani T, El Ouaamari A, Jungtrakoon P, Okawa ER, et al. Insulin Signaling Regulates the FoxM1/PLK1/CENP-A Pathway to Promote Adaptive Pancreatic β Cell Proliferation. *Cell Metab.* 2017;25(4):868-82.e5.
5. Campbell JE, Ussher JR, Mulvihill EE, Kolic J, Baggio LL, Cao X, et al. TCF1 links GIPR signaling to the control of beta cell function and survival. *Nat Med.* 2016;22(1):84-90.
6. Meng ZX, Gong J, Chen Z, Sun J, Xiao Y, Wang L, et al. Glucose Sensing by Skeletal Myocytes Couples Nutrient Signaling to Systemic Homeostasis. *Mol Cell.* 2017;66(3):332-44 e4.

7. Xiao X, Guo P, Prasadan K, Shiota C, Peirish L, Fischbach S, et al. Pancreatic cell tracing, lineage tagging and targeted genetic manipulations in multiple cell types using pancreatic ductal infusion of adeno-associated viral vectors and/or cell-tagging dyes. *Nat Protoc.* 2014;9(12):2719-24.
8. Meng ZX, Li S, Wang L, Ko HJ, Lee Y, Jung DY, et al. Baf60c drives glycolytic metabolism in the muscle and improves systemic glucose homeostasis through Deptor-mediated Akt activation. *Nat Med.* 2013;19(5):640-5.
9. Guest PC, Rhodes CJ, and Hutton JC. Regulation of the biosynthesis of insulin-secretory-granule proteins. Co-ordinate translational control is exerted on some, but not all, granule matrix constituents. *Biochem J.* 1989;257(2):431-7.
10. Lord SJ, Velle KB, Mullins RD, and Fritz-Laylin LK. SuperPlots: Communicating reproducibility and variability in cell biology. *J Cell Biol.* 2020;219(6).
11. Roux KJ, Kim DI, Raida M, and Burke B. A promiscuous biotin ligase fusion protein identifies proximal and interacting proteins in mammalian cells. *J Cell Biol.* 2012;196(6):801-10.
12. Ho SN, Hunt HD, Horton RM, Pullen JK, and Pease LR. Site-directed mutagenesis by overlap extension using the polymerase chain reaction. *Gene.* 1989;77(1):51-9.
13. Li S, Liu C, Li N, Hao T, Han T, Hill DE, et al. Genome-wide coactivation analysis of PGC-1alpha identifies BAF60a as a regulator of hepatic lipid metabolism. *Cell Metab.* 2008;8(2):105-17.

14. Yan M, Qi H, Xia T, Zhao X, Wang W, Wang Z, et al. Metabolomics profiling of metformin-mediated metabolic reprogramming bypassing AMPKalpha. *Metabolism.* 2019;91:18-29.

15. Zeng J, Wang Z, Huang X, Eckstein SS, Lin X, Piao H, et al. Comprehensive Profiling by Non-targeted Stable Isotope Tracing Capillary Electrophoresis-Mass Spectrometry: A New Tool Complementing Metabolomic Analyses of Polar Metabolites. *Chemistry.* 2019;25(21):5427-32.

16. Love MI, Huber W, and Anders S. Moderated estimation of fold change and dispersion for RNA-seq data with DESeq2. *Genome Biology.* 2014;15(12):550.

17. Yu G, Wang LG, Han Y, and He QY. clusterProfiler: an R package for comparing biological themes among gene clusters. *OMICS.* 2012;16(5):284-7.

18. Buenrostro JD, Giresi PG, Zaba LC, Chang HY, and Greenleaf WJ. Transposition of native chromatin for fast and sensitive epigenomic profiling of open chromatin, DNA-binding proteins and nucleosome position. *Nat Methods.* 2013;10(12):1213-8.

19. Langmead B, and Salzberg SL. Fast gapped-read alignment with Bowtie 2. *Nature Methods.* 2012;9(4):357-9.

20. Liu T, Mi L, Xiong J, Orchard P, Yu Q, Yu L, et al. BAF60a deficiency uncouples chromatin accessibility and cold sensitivity from white fat browning. *Nature Communications.* 2020;11(1):2379.

21. Zhang Y, Liu T, Meyer CA, Eeckhoute J, Johnson DS, Bernstein BE, et al. Model-based analysis of ChIP-Seq (MACS). *Genome Biol.* 2008;9(9):R137.

22. Heinz S, Benner C, Spann N, Bertolino E, Lin YC, Laslo P, et al. Simple combinations of lineage-determining transcription factors prime cis-regulatory elements required for macrophage and B cell identities. *Mol Cell.* 2010;38(4):576-89.

23. Robinson JT, Thorvaldsdóttir H, Winckler W, Guttman M, Lander ES, Getz G, et al. Integrative genomics viewer. *Nature Biotechnology.* 2011;29(1):24-6.

24. Ramirez F, Ryan DP, Gruning B, Bhardwaj V, Kilpert F, Richter AS, et al. deepTools2: a next generation web server for deep-sequencing data analysis. *Nucleic Acids Res.* 2016;44(W1):W160-5.

25. Grant CE, Bailey TL, and Noble WS. FIMO: scanning for occurrences of a given motif. *Bioinformatics.* 2011;27(7):1017-8.

26. Pique-Regi R, Degner JF, Pai AA, Gaffney DJ, Gilad Y, and Pritchard JK. Accurate inference of transcription factor binding from DNA sequence and chromatin accessibility data. *Genome Res.* 2011;21(3):447-55.

27. Kaya-Okur HS, Wu SJ, Codomo CA, Pledger ES, Bryson TD, Henikoff JG, et al. CUT&Tag for efficient epigenomic profiling of small samples and single cells. *Nature Communications.* 2019;10(1):1930.

28. Han X, Wang R, Zhou Y, Fei L, Sun H, Lai S, et al. Mapping the Mouse Cell Atlas by Microwell-Seq. *Cell.* 2018;172(5):1091-107.e17.

29. Picelli S, Björklund ÅK, Faridani OR, Sagasser S, Winberg G, and Sandberg R. Smart-seq2 for sensitive full-length transcriptome profiling in single cells. *Nature Methods.* 2013;10(11):1096-8.

30. Dobin A, Davis CA, Schlesinger F, Drenkow J, Zaleski C, Jha S, et al. STAR: ultrafast universal RNA-seq aligner. *Bioinformatics.* 2012;29(1):15-21.

31. Stuart T, Butler A, Hoffman P, Hafemeister C, Papalexi E, Mauck WM, III, et al. Comprehensive Integration of Single-Cell Data. *Cell.* 2019;177(7):1888-902.e21.

32. Wickham H. *ggplot2: elegant graphics for data analysis.* Springer; 2016.

33. Wang IM, Zhang B, Yang X, Zhu J, Stepaniants S, Zhang C, et al. Systems analysis of eleven rodent disease models reveals an inflammatome signature and key drivers. *Mol Syst Biol.* 2012;8:594.

34. Ritchie ME, Phipson B, Wu D, Hu Y, Law CW, Shi W, et al. limma powers differential expression analyses for RNA-sequencing and microarray studies. *Nucleic acids research.* 2015;43(7):e47-e.

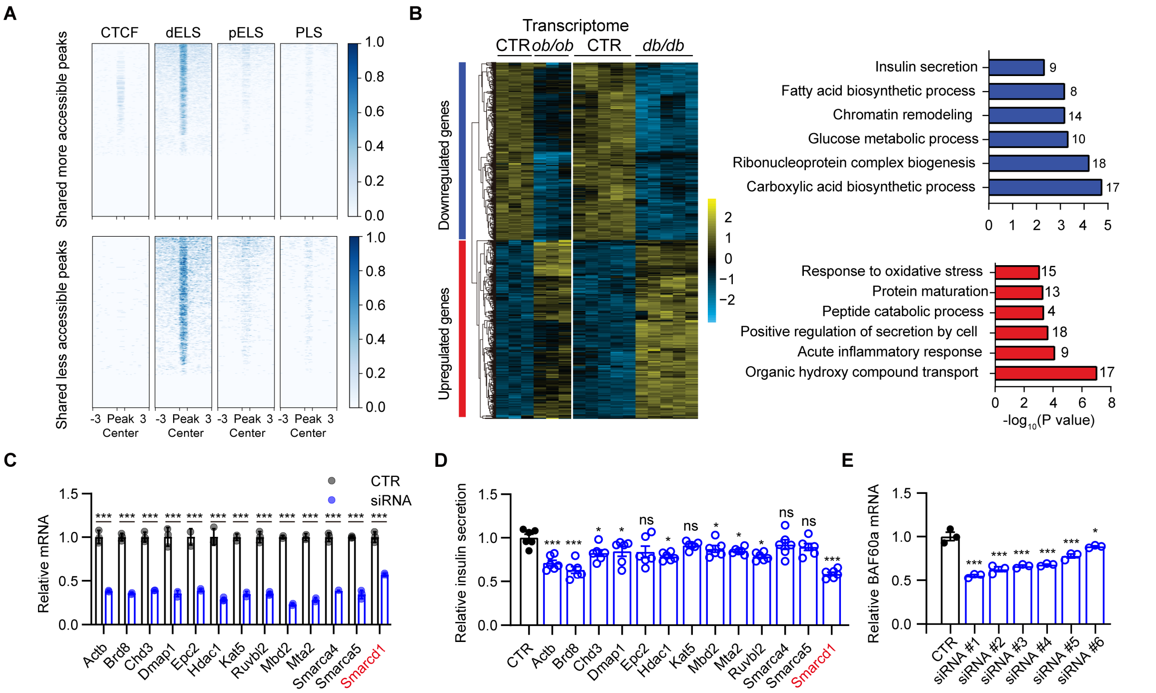

G

Patient characteristics	Age(year)	BMI(kg/m²)	FPG(mM)	Male(%)	Female(%)
CTR (n = 21)	49.76 ± 10.94	23.38 ± 2.54	5.65 ± 0.72	23.81	76.19
T2D (n = 21)	55.10 ± 10.12	23.05 ± 3.34	10.63 ± 3.07	28.57	71.43
P value	0.11	0.74	<0.0001		

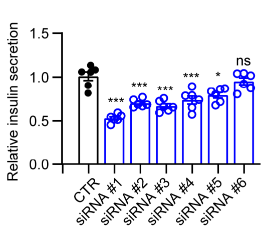

Supplemental Figure S1. Identification of BAF60a as a chromatin remodeling checkpoint factor for β-cell dysfunction in T2D. Related to Figure 1.

(A) Density distribution of shared ATAC differential accessible peaks in *db/db* and *ob/ob* mice around the ENCODE cis-regulatory elements. Signals on CTCF-Binding regions (CTCF), distal enhancer-like signals (dELS), proximal enhancer-like signals (pELS), and promoter-like signals (PLS) are shown. Peaks were scaled to 800 bp for optimal visualization.

(B) Heatmap (left) and GO analysis (right) of shared downregulated or upregulated genes in islets from *ob/ob* and *db/db* mice. (n = 3-5 mice per group, $|\log_2FC|>0.3$, False Discovery Rate (FDR) < 0.05).

(C) Relative mRNA expression of Min6 cells transfected with siRNAs targeting different chromatin modifiers. mRNA expression levels in cells transfected with control siRNA were used for normalization. Mean ± s.d. (n = 3 technical replicates); two-tailed

unpaired Student’s t-test, ***, $P < 0.001$.

(D) Relative GSIS of Min6 cells transfected with siRNAs targeting different chromatin modifiers. GSIS levels were normalized to cells transfected with control siRNA. Mean ± s.e.m. (n = 6 biological replicates); one-way ANOVA. ns, not statistically significant; *, $P < 0.05$; **, $P < 0.01$; ***, $P < 0.001$.

(E) Relative *BAF60a* expression of Min6 cells transfected with different siRNAs targeting *BAF60a*. *BAF60a* levels were normalized to cells transfected with control siRNA. Mean ± s.d. (n = 3 technical replicates); one-way ANOVA. ns, not statistically significant; *, $P < 0.05$; **, $P < 0.01$; ***, $P < 0.001$.

(F) Relative GSIS of Min6 cells transfected with different siRNAs targeting *BAF60a*. insulin levels were normalized to cells transfected with control siRNA. Mean ± s.e.m. (n = 6 biological replicates); one-way ANOVA. *, $P < 0.05$; **, $P < 0.01$; ***, $P < 0.001$.

(G) Donor information of pancreas sections from control and diabetic patients. Mean ± s.e.m. (n = 21 donors in each group).

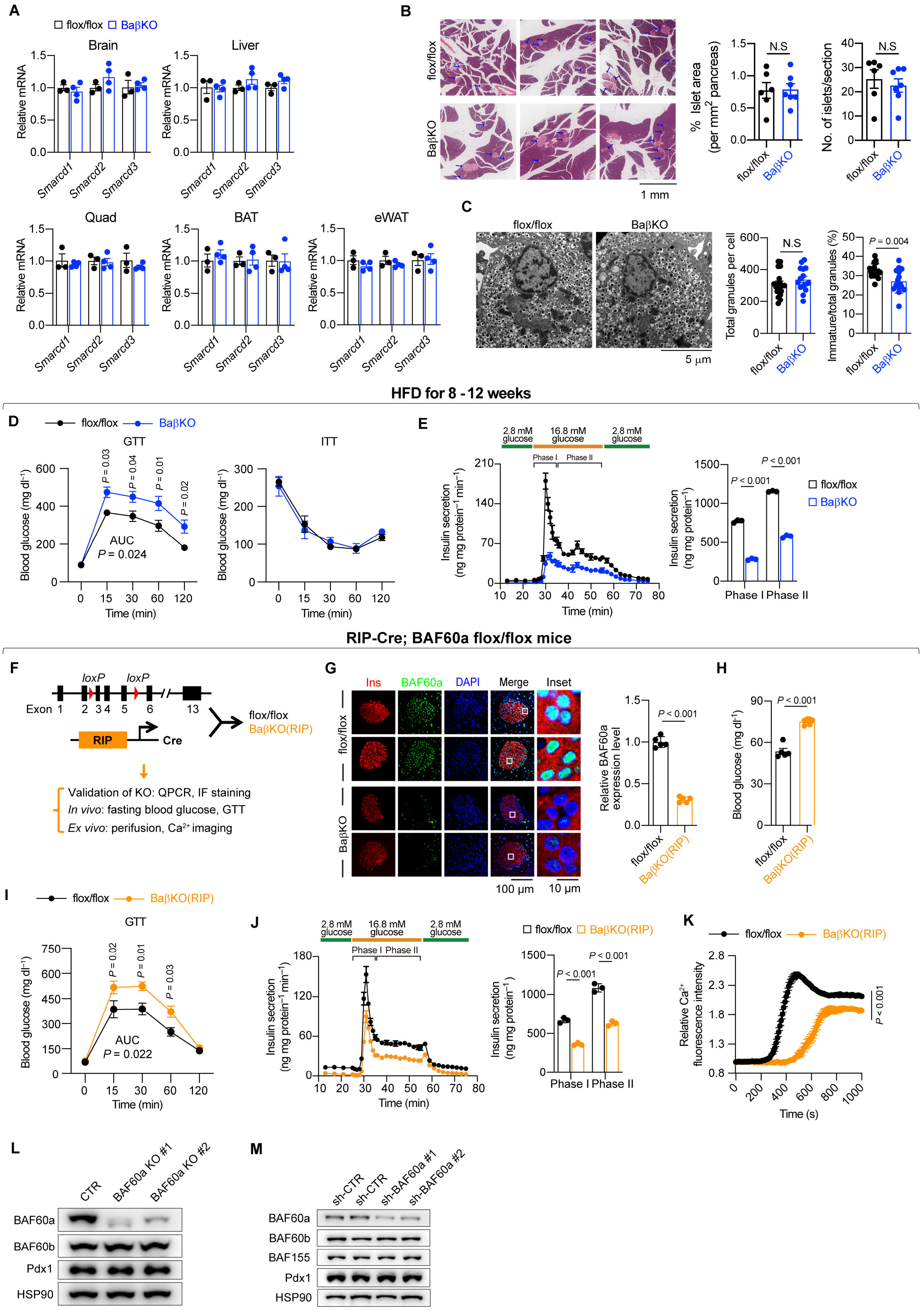
A
flox/flox
BaβKO
Brain
Liver
Quad
BAT
eWAT
Relative mRNA
Smarcd1
Smarcd2
Smarcd3
B
flox/flox
BaβKO
1 mm
% Islet area (per mm² pancreas)
No. of islets/section
N.S
C
5 μm
Total granules per cell
Immature/total granules (%)
P = 0.004
HFD for 8 - 12 weeks
D
GTT
ITT
Blood glucose (mg dl⁻¹)
Time (min)
AUC
P = 0.024
P = 0.03
P = 0.04
P = 0.01
P = 0.02
E
2.8 mM glucose
16.8 mM glucose
Phase I
Phase II
Insulin secretion (ng mg protein⁻¹ min⁻¹)
Insulin secretion (ng mg protein⁻¹)
P < 0.001
RIP-Cre; BAF60a flox/flox mice
F
loxP
Exon
RIP
Cre
BaβKO(RIP)
Validation of KO: QPCR, IF staining
In vivo: fasting blood glucose, GTT
Ex vivo: perifusion, Ca²⁺ imaging
G
Ins
BAF60a
DAPI
Merge
Inset
100 μm
10 μm
Relative BAF60a expression level
H
I
AUC
P = 0.022
J
K
Relative Ca²⁺ fluorescence intensity
Time (s)
L
CTR
BAF60a KO #1
BAF60a KO #2
BAF60a
BAF60b
Pdx1
HSP90
M
sh-CTR
sh-BAF60a #1
sh-BAF60a #2
BAF155

Supplemental Figure S2. Phenotypic studies of mouse models of β-cell-specific BAF60a deficiency. Related to Figure 2.

(A) Relative *BAF60a/b/c* mRNA expression in indicated tissues from flox/flox and BaβKO mice. Expression levels were normalized to their respective controls. Mean ± s.e.m. (n = 3-4 mice per group); two-tailed unpaired Student's t-test with multiple comparisons corrected by FDR.

(B) Representative H&E staining of pancreas sections from flox/flox and BaβKO mice (left), and size and number of islets per mouse (right). Scale bar, 1 mm. Mean ± s.e.m. (n = 6-7 mice per group); two-tailed unpaired Student's t-test.

(C) Representative electron micrographs of islets from flox/flox and BaβKO mice (left), total insulin granules per cell (middle), and the percentage of immature insulin granules (right) are shown. Mean ± s.d. (n = 15-18 islets per group); two-tailed unpaired Student's t-test. Scale bar, 5 μm.

(D) IPGTT (left) and ITT (right) of flox/flox and BaβKO mice in HFD condition. Mean ± s.e.m. (n = 5-6 mice per group); two-way ANOVA.

(E) Dynamic glucose-stimulated insulin secretion (left) and biphasic insulin release levels (right) of islets from flox/flox and BaβKO mice in HFD condition. The islet sample in each group was pooled from 3 mice. Mean ± s.d. (n = 3 technical replicates); two-tailed unpaired Student's t-test.

(F) Schematic representation of experimental method related to BaβKO (RIP-Cre) mouse model.

(G) Representative immunofluorescent images of pancreas sections from flox/flox and BaβKO (RIP-Cre) mice using indicated antibodies (left) and quantification of BAF60a expression levels (right). Mean ± s.e.m. (n = 5 mice per group); two-tailed unpaired Student's t-test. Scale bar, 100 μm and 10 μm.

(H) Overnight fasting blood glucose levels in flox/flox and BaβKO (RIP-Cre) mice. Mean ± s.e.m. (n = 5-6 mice per group); two-tailed unpaired Student's t-test.

(I) IPGTT of flox/flox and BaβKO (RIP-Cre) mice. Mean ± s.e.m. (n = 3-4 mice per group); two-way ANOVA.

(J) Dynamic glucose-stimulated insulin secretion (left) and biphasic insulin release levels (right) of islets from flox/flox and BaβKO (RIP-Cre) mice. The islet sample in

each group was pooled from 3 mice. Mean ± s.d. (n = 3 technical replicates); two-tailed unpaired Student's t-test.

(K) Intracellular Ca^{2+} levels in isolated islets from flox/flox and BaβKO (RIP-Cre) mice. The islet sample in each group was pooled from 3 mice. Mean ± s.e.m. (n = 41-63 islets per group); two-way ANOVA.

(L) Immunoblots of total cell lysates from control (CTR) and BAF60a knockout Min6 cells.

(M) Immunoblots of total cell lysates from control (sh-CTR) and BAF60a knockdown Min6 cells.

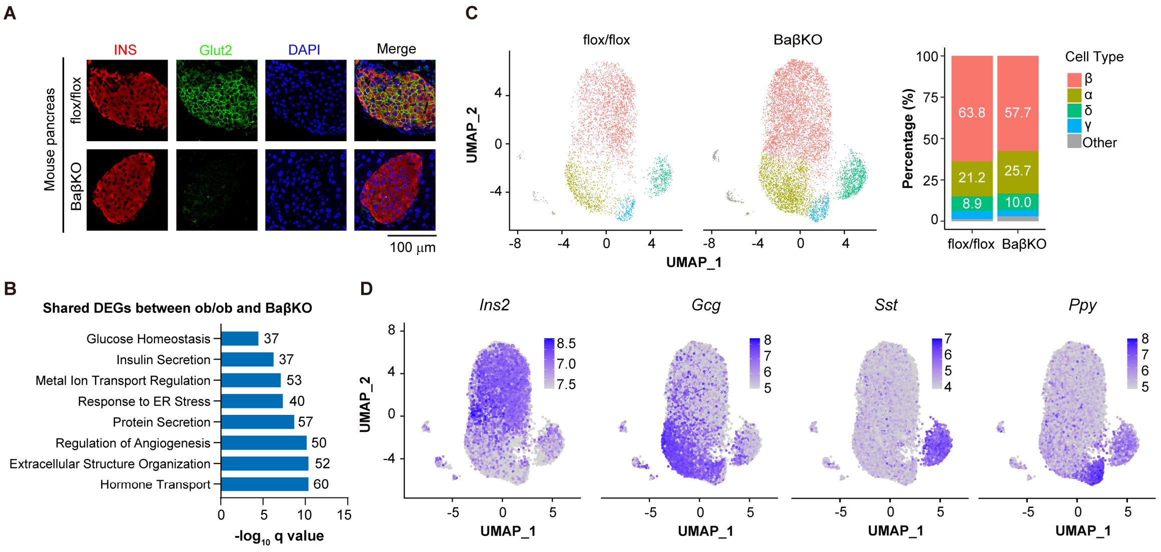

Supplemental Figure S3. Effects of BAF60a deficiency on islet cell composition and transcriptional profile. Related to Figure 3.

(A) Representative immunofluorescent images of islets from the indicated groups.

(B) GO analysis of shared DEGs between *ob/ob* and BaβKO mouse models.

(C) UMAP visualization of cell clustering and cell composition in flox/flox and BaβKO mouse islets as revealed by scRNA-Seq. Key endocrine cell types (β-, α-, δ-, and γ-cells) are color-coded.

(D) Feature plots demonstrating the cell-type marker in each cluster.

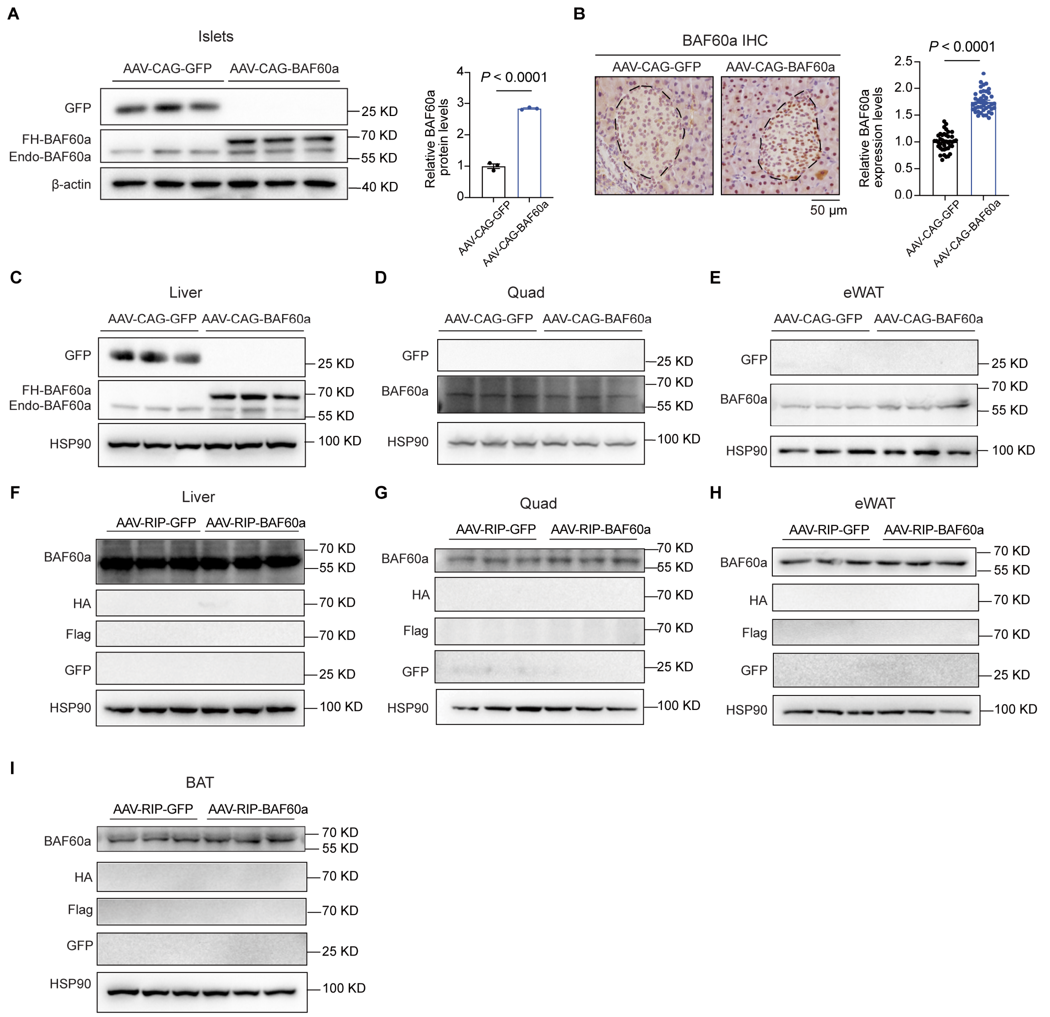

Supplemental Figure S4. Tissue distribution of exogenous BAF60a after pancreatic ductal infusion of AAVs. Related to Figure 4.

(A) Immunoblots of BAF60a expression in islets upon pancreatic ductal infusion of AAV-CAG-GFP or AAV-CAG-BAF60a. Mean ± s.e.m. (n = 3 biological replicates); two-tailed unpaired Student's t-test.

(B) IHC analysis of pancreas sections in islets upon pancreatic ductal infusion of AAV-CAG-GFP or AAV-CAG-BAF60a. Mean ± s.e.m. (n = 41 and 43 islets for AAV-CAG-GFP group and AAV-CAG-BAF60a group, respectively); two-tailed unpaired Student's t-test.

(C) Immunoblots of BAF60a expression in mouse liver as described in (A).

(D) Immunoblots of BAF60a expression in quadriceps (Quad) muscle as described in (A).

(E) Immunoblots of BAF60a expression in epididymal white adipose tissue (eWAT) as described in (A).

(F) Immunoblots of BAF60a expression in liver upon pancreatic ductal infusion of AAV-RIP-GFP or AAV-RIP-BAF60a. n = 3 biological replicates.

(G) Immunoblots of BAF60a expression in Quad muscle as described in (F).

(H) Immunoblots of BAF60a expression in eWAT as described in (F).

(I) Immunoblots of BAF60a expression in brown adipose tissue (BAT) as described in (F).

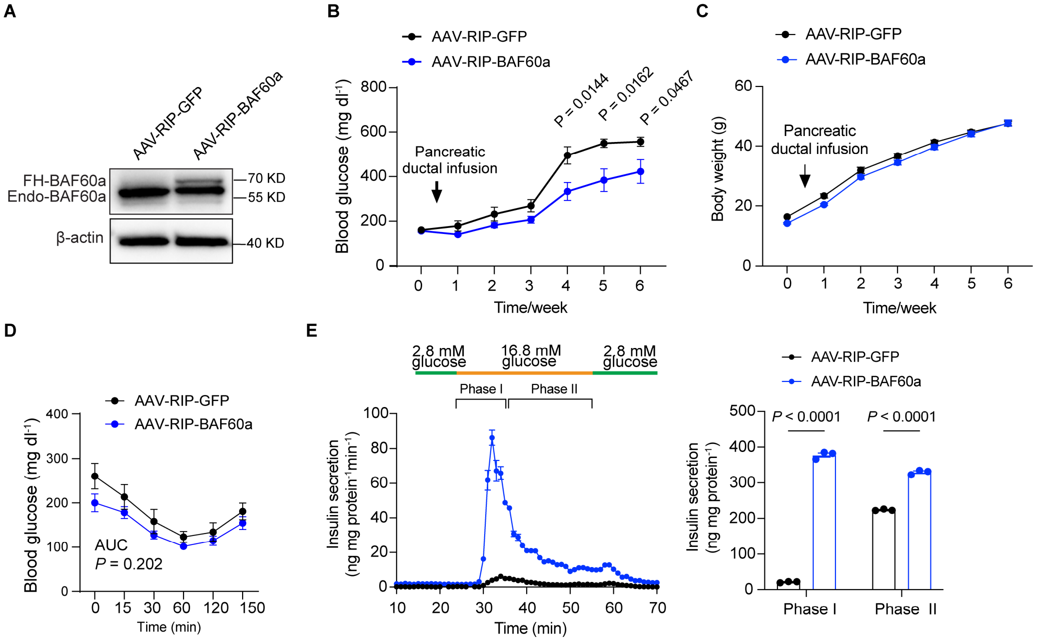

Supplemental Figure S5. AAV-mediated β-cell-specific restoration of BAF60a improves β-cell function and glycemic control. Related to Figure 4.

(A) Immunoblots of mouse islets following pancreatic ductal infusion of AAV-RIP-GFP or AAV-RIP-BAF60a for 5 days.

(B) Random blood glucose levels in BKS-*db/db* mice following pancreatic ductal infusion of AAV-RIP-GFP or AAV-RIP-BAF60a. Mean ± s.e.m. (n = 6-7 mice per group); two-way ANOVA.

(C) Body weight change of mice described in (B); two-way ANOVA.

(D) ITT of mice described in (B); two-way ANOVA.

(E) Dynamic glucose-stimulated insulin secretion (left) and biphasic insulin release levels (right) of mouse islets from the indicated groups. The islet sample in each group was pooled from 3 mice. Mean ± s.d. (n = 3 technical replicates); two-tailed unpaired Student’s t-test.

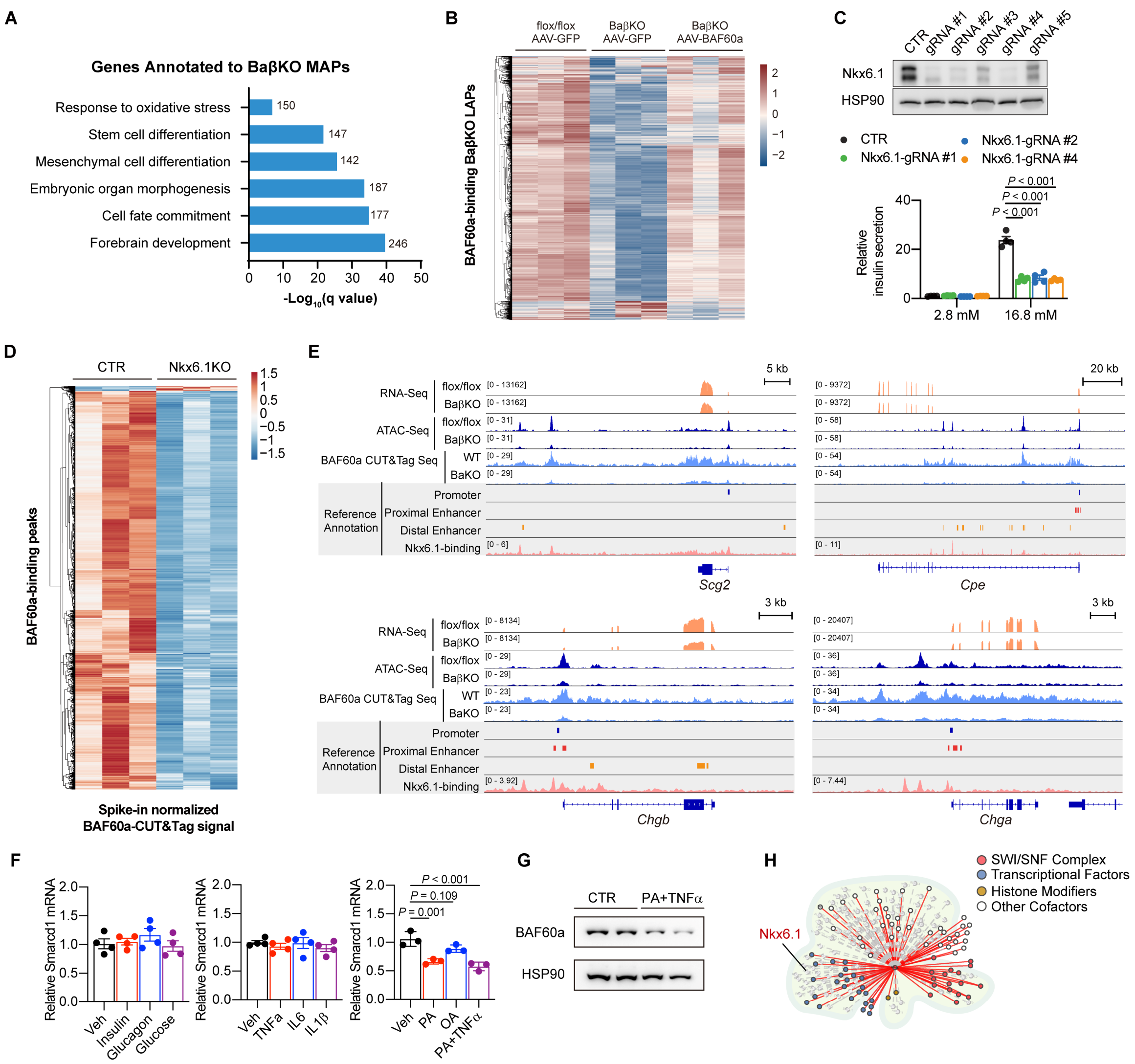

Supplemental Figure S6. Multi-omics analysis of control and BaβKO mouse islets. Related to Figure 5.

(A) GO enrichment analysis of the genes annotated to the more-accessible peaks (MAPs) in BaβKO primary islets.

(B) Chromatin accessibility heatmap of BAF60a-binding BaβKO less-accessible peaks (LAPs) in flox/flox or BaβKO mice underwent pancreatic ductal infusion of AAV-GFP or AAV-RIP-BAF60a.

(C) Immunoblots (up) and GSIS (low) of control (CTR) and Nkx6.1 knock-out Min6 cells. Mean ± s.e.m. (n = 4 biological replicates); two-tailed unpaired Student’s t-test.

(D) Spike-in normalized BAF60a CUT&Tag signals on BAF60a binding peaks in control

(CTR) or the Nkx6.1 KO (Nkx6.1KO) Min6 cells.

(E) Representative RNA-Seq, ATAC-Seq, and CUT&Tag-Seq browser tracks displaying BAF60a-regulated, islet β-cell specific gene loci, including *Scg2*, *Cpe*, *Chga*, and *Chgb*.

(F) Relative *BAF60a* mRNA expression in Min6 cells treated with indicated treatments for 12 h. Mean ± s.e.m. (n = 3-4 biological replicates); Expression levels were normalized to their respective controls; one-way ANOVA; Veh, Vehicle.

(G) Immunoblots of total protein lysates in isolated islets treated without (CTR) or with PA (0.5 mM) and TNFα (50 ng/mL) for 24 h.

(H) Integrated BAF60a interactomes in Min6 cells in the presence or absence of PA (0.5 mM) and TNFα (50 ng/mL) treatment. The complete BAF60a interactome in untreated cells is shown in a green cloud. BAF60a interactome in PA (0.5 mM) and TNFα (50 ng/mL) treated cells are shown in color with red lines connected. Lost interactions in response to PA (0.5 mM) and TNFα (50 ng/mL) treatment are shown in gray lines.

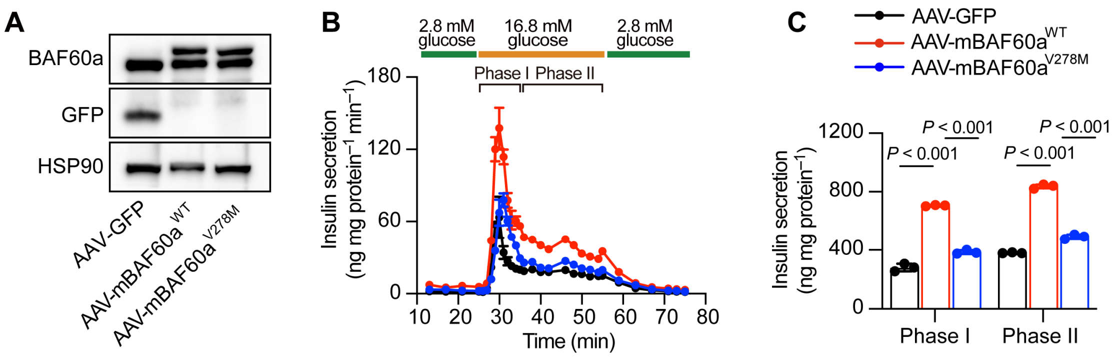

Supplemental Figure S7. Effects of BAF60a^{WT} and BAF60a^{V278M} overexpression on mouse islet insulin secretion. Related to Figure 7.

(A-C) Immunoblots (A), dynamic glucose-stimulated insulin secretion (B) and biphasic insulin release levels (C) of C57BL/6J mouse islets transduced with AAVs expressing BAF60a^{WT}, BAF60a^{V278M}, or GFP for 5 days. Mean ± s.d. (n = 3 technical replicates); one-way ANOVA.

Supplemental Table S1. siRNA target sequences for siRNA-screening of chromatin remodeling genes. Related to Figure 1.

Gene	Target Sequence
Actb	GCAAGAGAGGTATCCTGACCCTGAA
Brd8	GAAAGGCGATGAGAGGCCACTTTCA
Chd3	GACAGTCAAAGAGGCAGCTTCGAAA
Dmap1	CAGATGTACGAGACATTCTAGAACT
Epc2	TCAAGCACCTCTGACTGTATGTCTA
Hdac1	GAGGGTGCTCTATATTGACATTGAT
Kat5	AGCCTGGACGGAAGCGGAAATCTAA
Mbd2	CCCTGCTGTTTGGCTTAACACATCT
Mta2	CCGAAGACCCTATGCACCTATCAAT
Ruvbl2	GAGTACCAAGATGCCTTCCTCTTCA
Smarca4	ACCCAAACTCCGTGCAACGAACCAT
Smarca5	GGAGGATGAAGAGCTATTA
Smarcd1	GACCCTCAGGGTTTCATCAATGATT
Smarcd1-2	GTAGCCGAATGACACCTCATT
Smarcd1-3	GCTAGATATCCAGGAGGCCTTGAAA
Smarcd1-4	GCGAGAGTTCATGTTGAGCTT
Smarcd1-5	CAGACACGTCCAGTGATCATCCAAG
Smarcd1-6	GAGCGAGAGTTTGTTCTCTGTGACA
Smarcd1-7	CAGGGACCTCAAGACGATGACTGAT

Supplemental Table S2. qPCR primer sequences used in this study. Related to Figures 1 and 8; Supplemental Figures S1 and S2.

Gene	Primer Sequence
Mouse *Smarcd1*	Forward: TGGACCCAAATGACCAGAAAA<br>Reverse: TCTTGTTGTCTAGAGTGGCGATCT
Mouse *Slc2a2*	Forward: CCGAACTGGAAGGAACTCAG<br>Reverse: GGATTAAGCGGACAATTCCA
Mouse *Pdx1*	Forward: TAGGCGTCGCACAAGAAGAA<br>Reverse: TCTTGTTGTCTAGAGTGGCGATCT
Mouse *Glp1r*	Forward: CGGAGTGTGAAGAGTCTAAGCG<br>Reverse: ATGGCTGAAGCGATGACCAAGG
Mouse *Gipr*	Forward: GTATGCCTGCTGGAACTACACG<br>Reverse: CACTGAGTGTGGTCTCTCCAAG
Mouse *Actb*	Forward: AGGCCAACCGTGAAAAGATG<br>Reverse: AGAGCATAGCCCTCGTAGATGG
Mouse *Brd8*	Forward: GGAAGACTTGGATCTAGCGGAG<br>Reverse: CACATCCAGCACTTCAGGGTGA
Mouse *Chd3*	Forward: CCACCTTCTCAACTTCCTCACC<br>Reverse: ACATCCGCCTTGAGTCTCCGAA
Mouse *Dmap1*	Forward: AGAGACGCTGACCTTCAAGAGG<br>Reverse: TTCGCCTTCACTGTCCGATACC
Mouse *Epc2*	Forward: GCATTCCTCACAGCAGACACATC<br>Reverse: CTTGGAACAGGAGCACTGACCA
Mouse *Hdac1*	Forward: TGAAGCCTCACCGAATCCGCAT<br>Reverse: TGGTCATCTCCTCAGCATTGGC
Mouse *Kat5*	Forward: TGAGCGTGAAGGACATCAGTGG<br>Reverse: TTAAGTCCAGCCGCTCGTGAGT
Mouse *Mbd2*	Forward: TCTGGGAGAAGAGGCTACAAGG<br>Reverse: GGTCTCGTCATTGCTACCTGGA
Mouse *Mta2*	Forward: TAGCCAGACGAATGTGCCGAGA<br>Reverse: TTGGCAGCCTTAGGAAGTCGGA

Mouse *Ruvbl2*	Forward: GGATACCAAGCAGATTCTGCGC<br>Reverse: GTGATGAGCTGGATGGCATAGC
Mouse *Smarca4*	Forward: CCCTCTGTGGTGAAGGTTTCTTA<br>Reverse: CTGCGAAGCTGTGGGACA
Mouse *Smarca5*	Forward: GCTGCTCAGAAGACTCCAACCT<br>Reverse: TCCTCTTGCTCCGTTCTACGGT
Mouse *Smarcd2*	Forward: GAAGCTGGACCAGACCATCG<br>Reverse: CGCAGTTCCCGCATTATCT
Mouse *Smarcd3*	Forward: AGGCTTACATGGACCTCCTAG<br>Reverse: CATCAGAGTCTTCCGCATCAG
Human *GLP1R*	Forward: CTACGCACTCTCCTTCTCTGCT<br>Reverse: CGGACAATGCTCGCAGGATGAA
Human *GIPR*	Forward: TGGCATTCTCCTGTCCAAGCTG<br>Reverse: GTTCCTCTGTCACGGGAGCAAA
Human *SMARCD1*	Forward: CATACCCAGACTCGTCCAGTGATC<br>Reverse: GCTGCAGGTACTTGTCACAGATGA

Supplemental Table S3 Clinical information of human subjects with BAF60a^{WT} or BAF60a^{V278M}. Related to Figure 6.

Variable	BAF60a^{WT} (n = 13842)	BAF60a^{V278M} (n = 61)	P value
Sex (male%)	0.42	0.52	0.11
Age (year)	55.85 ± 7.33	53.80 ± 6.10	0.03
Body mass index (kg/m^2)	24.90 ± 3.27	24.56 ± 3.44	0.44
Fasting glucose (mmol/L)	5.76 (5.30,6.35)	5.69 (5.28,6.33)	0.49
30 minutes glucose (mmol/L)	10.00 (8.63,11.56)	9.76 (7.86,11.33)	0.22
2 hours glucose (mmol/L)	7.49 (6.00,9.65)	6.90 (5.75,8.77)	0.14
LDL-C (mmol/L)	3.03 (2.50,3.60)	2.84 (2.49,3.21)	0.05
HDL-C (mmol/L)	1.27 (1.07,1.50)	1.26 (1.10,1.50)	0.73
Total cholesterol	5.10 (4.51,5.77)	4.81 (4.44,5.35)	0.03
HOMA-IR	2.01 (1.38,3.00)	1.87 (1.42,2.47)	0.32
