# Supplementary material for: BAF60a-dependent chromatin remodeling preserves β cell function and contributes to the therapeutic benefits of GLP-1R agonists
Source: J Clin Invest. 2025 Oct 2;135(23):e177980. doi: 10.1172/JCI177980 (PMC12646663; doi:10.1172/JCI177980)

BAF60a-dependent chromatin remodeling preserves β-cell function and contributes to the therapeutic benefits of GLP-1R agonists

JCI unedited blot

Full unedited blot for Fig 1.J

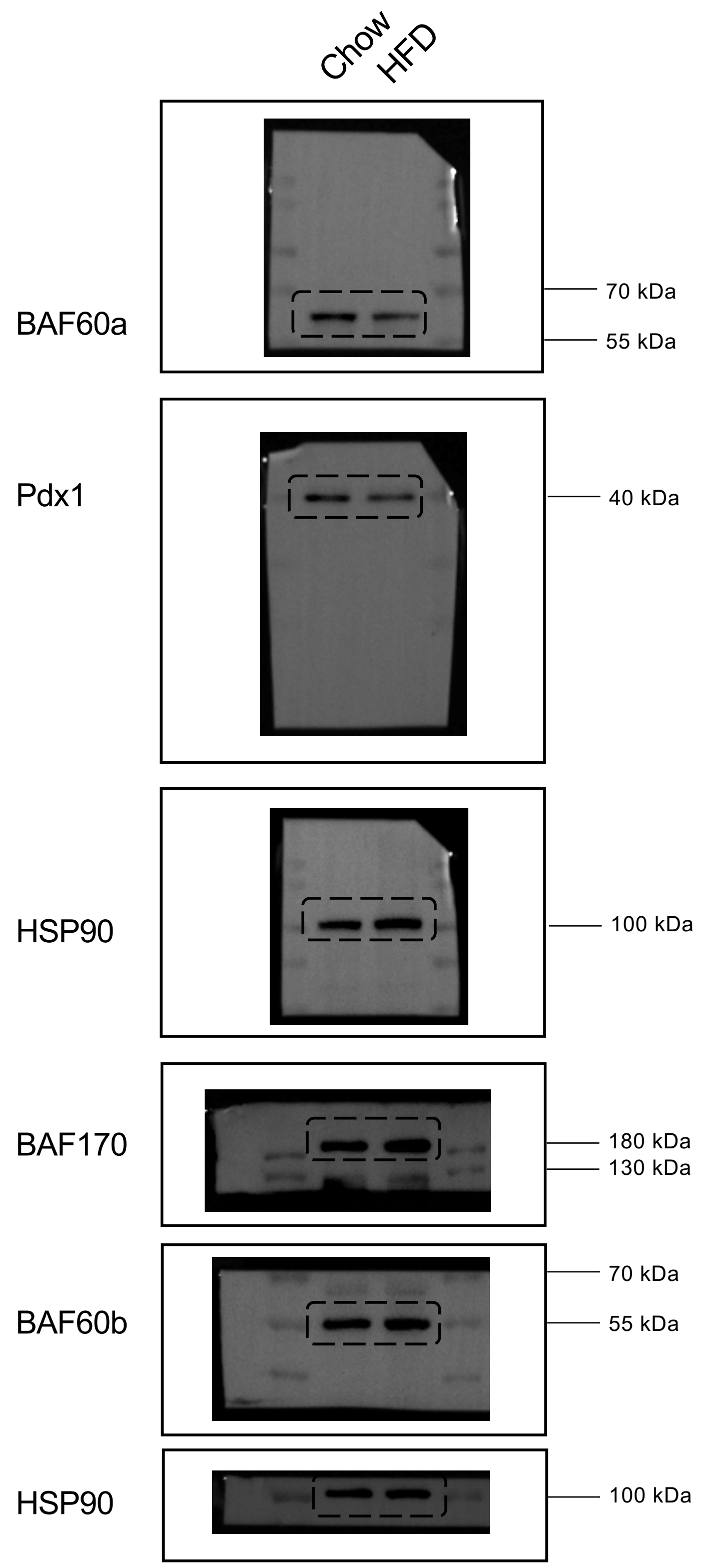

Full unedited blot for Fig 1.J

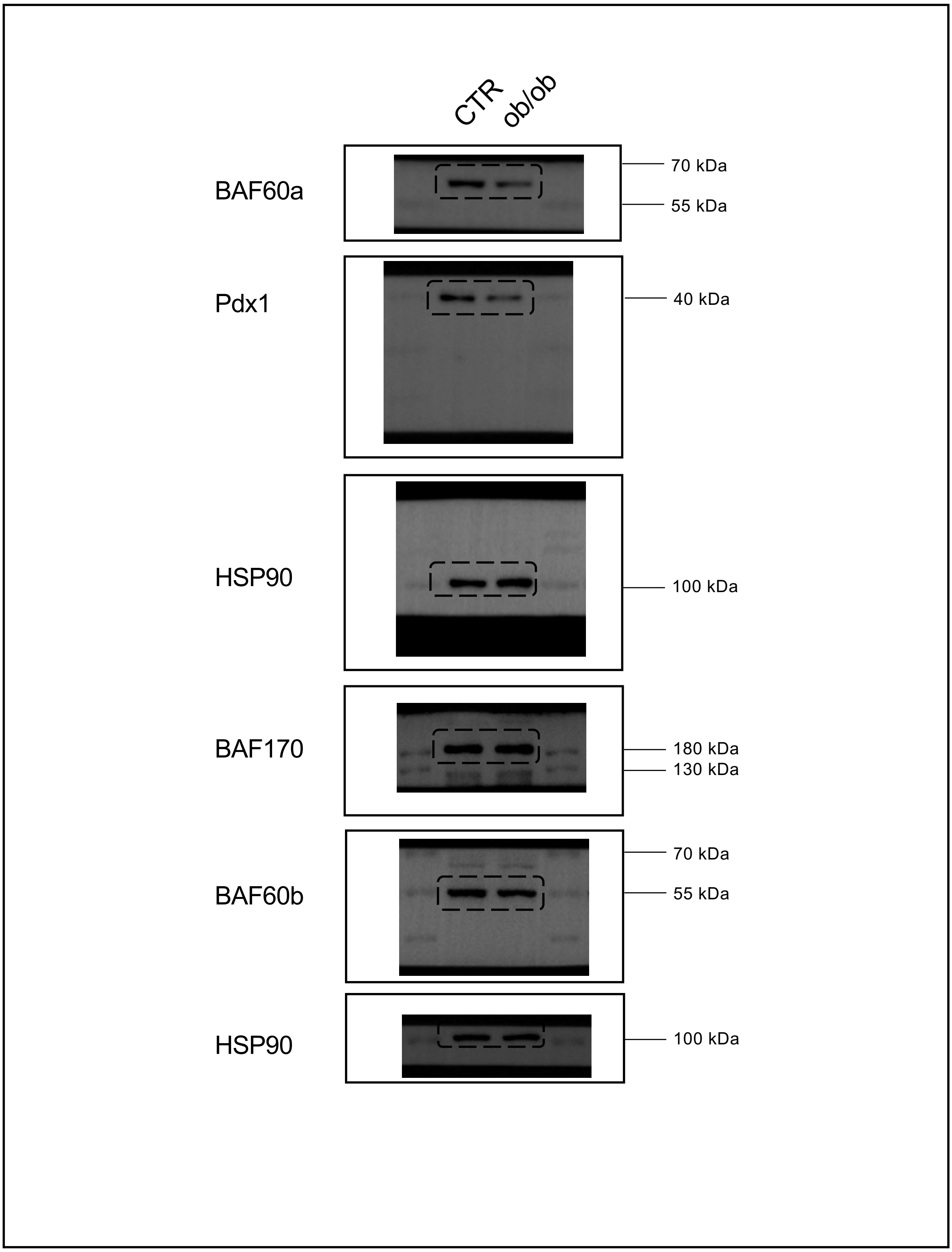

Full unedited blot for Fig 1.J

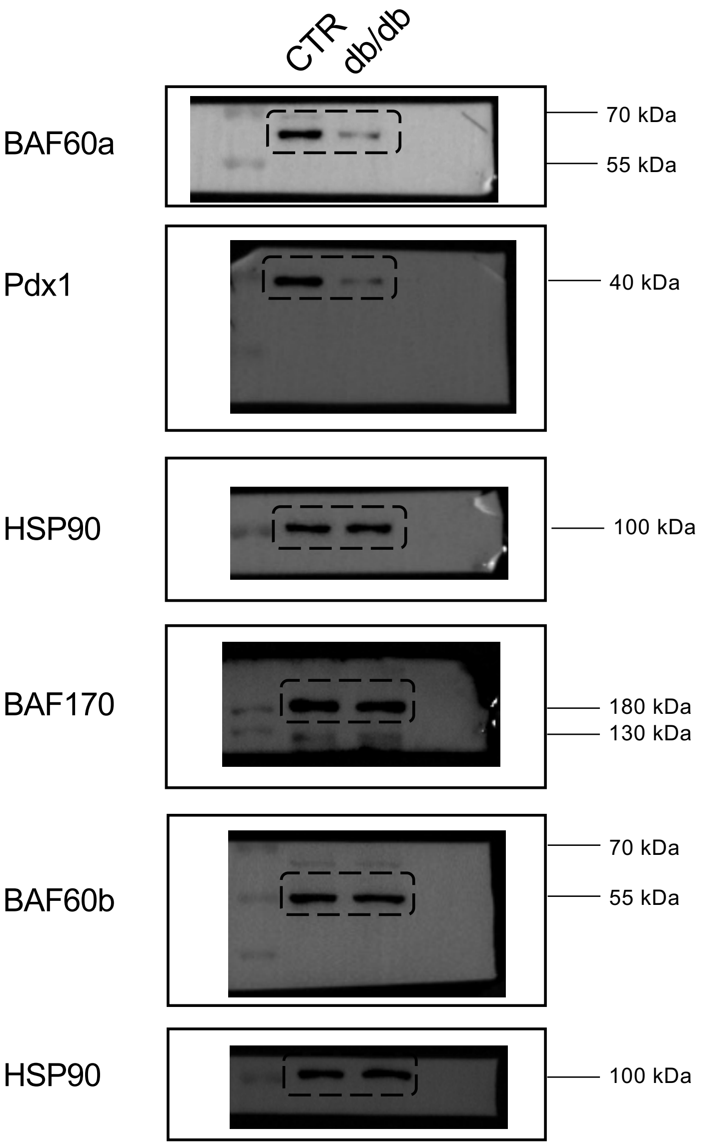

Full unedited blot for Fig 2.C

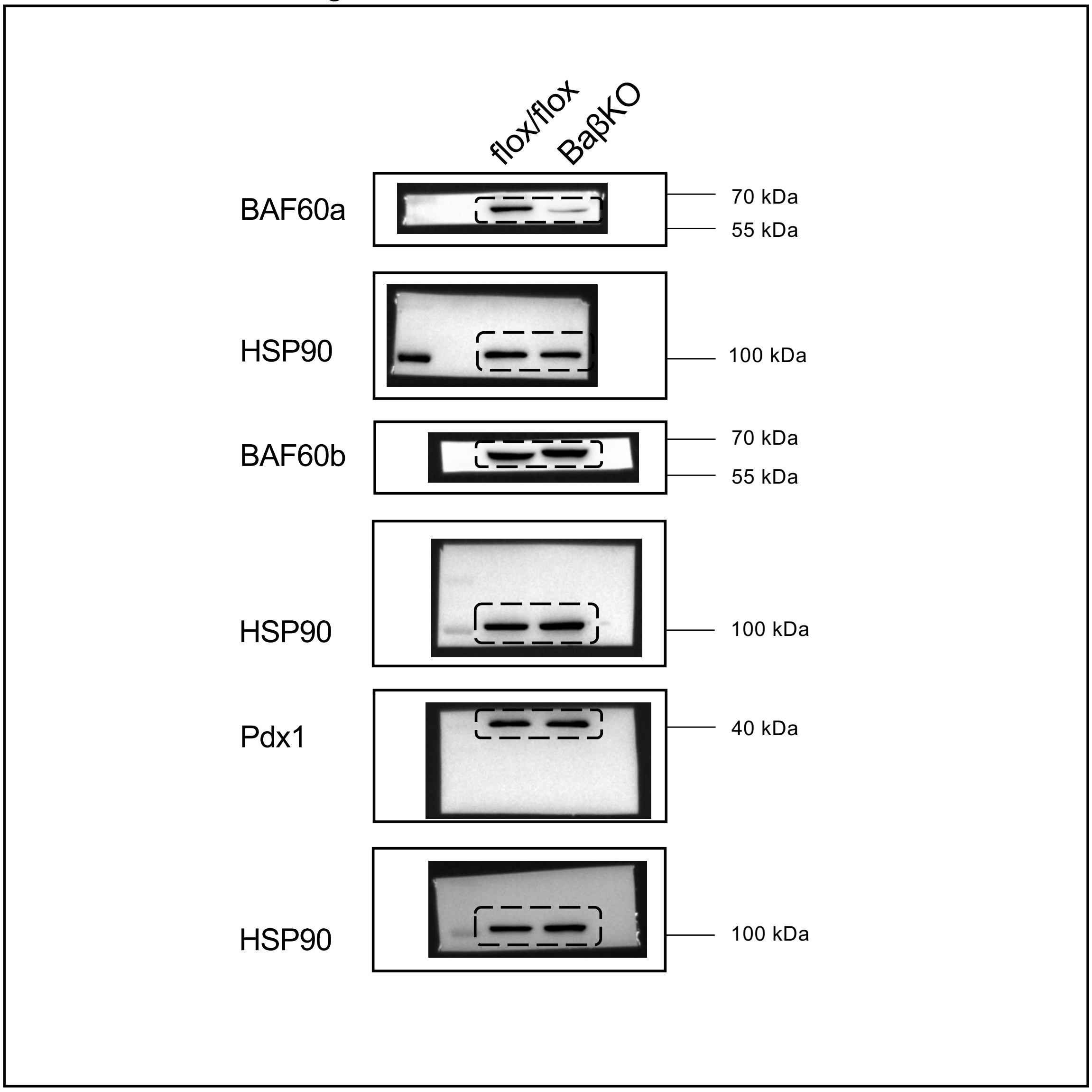

Full unedited blot for Fig 4.M

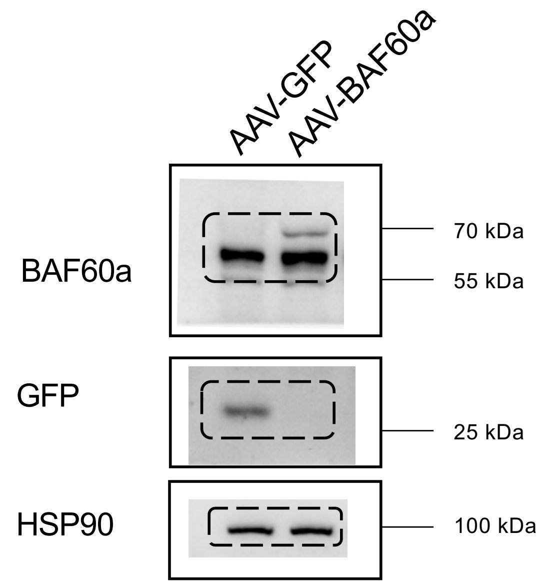

Full unedited blot for Fig 5.K

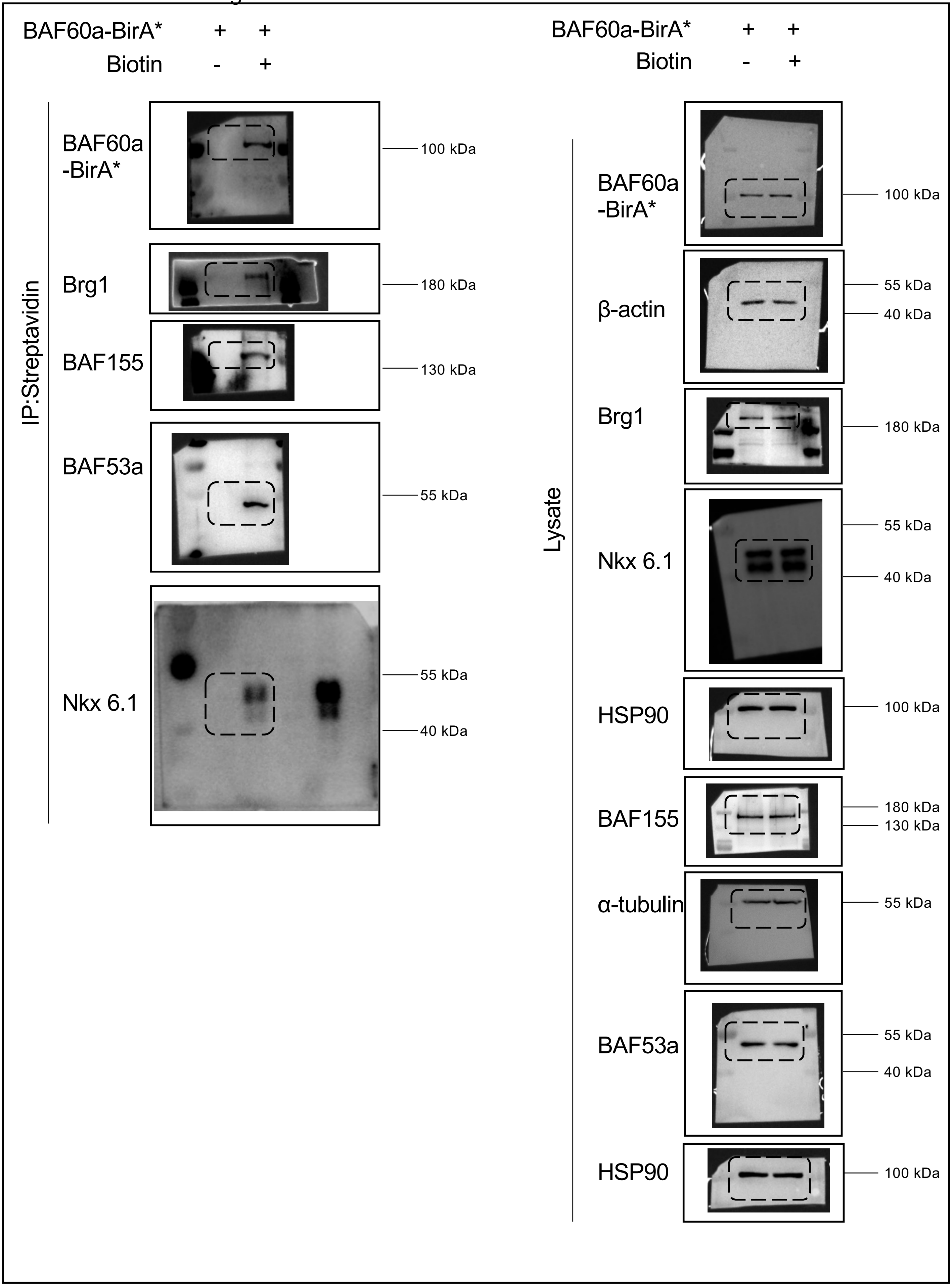

Full unedited blot for Fig 5.K

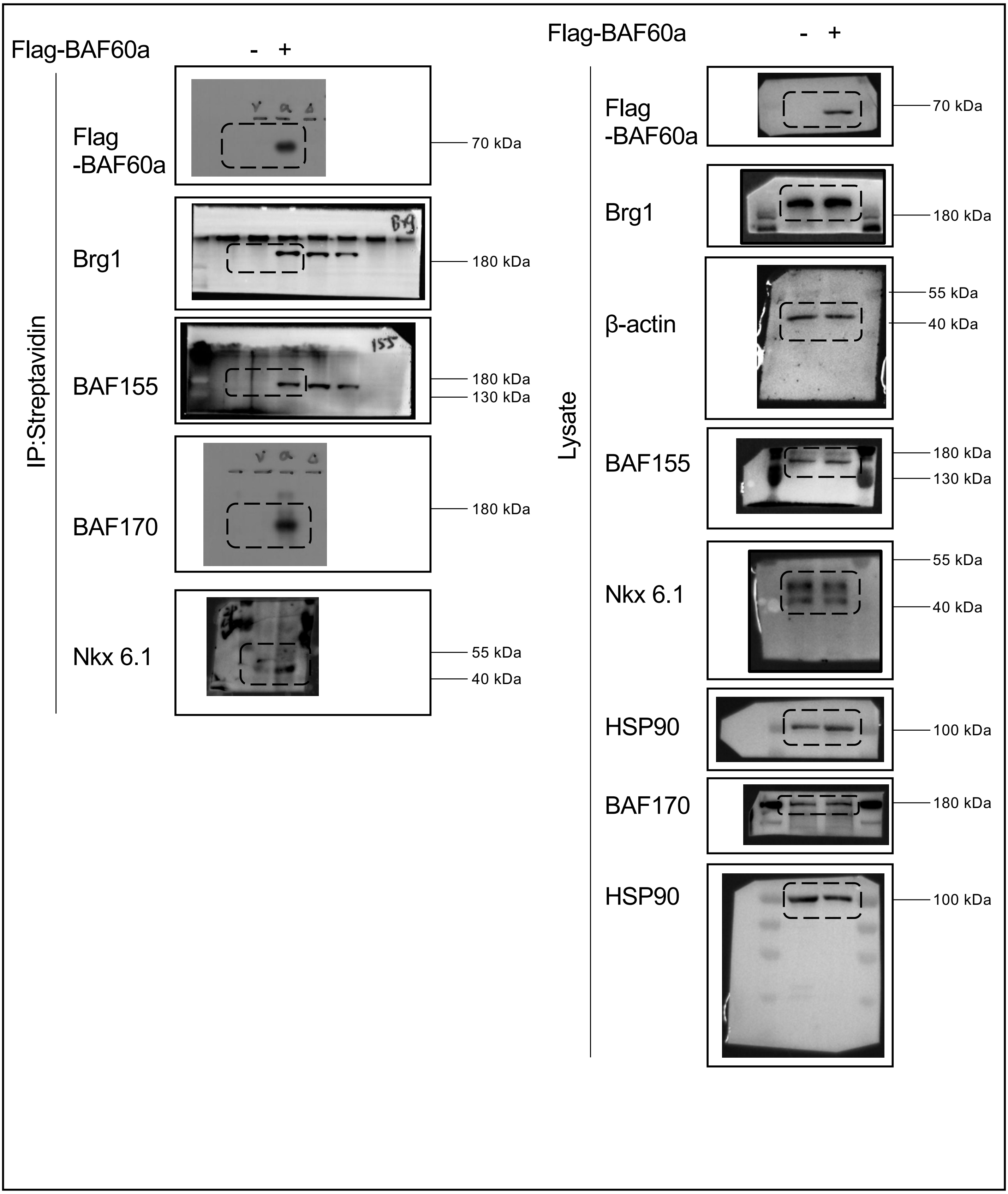

Full unedited blot for Fig 5.L

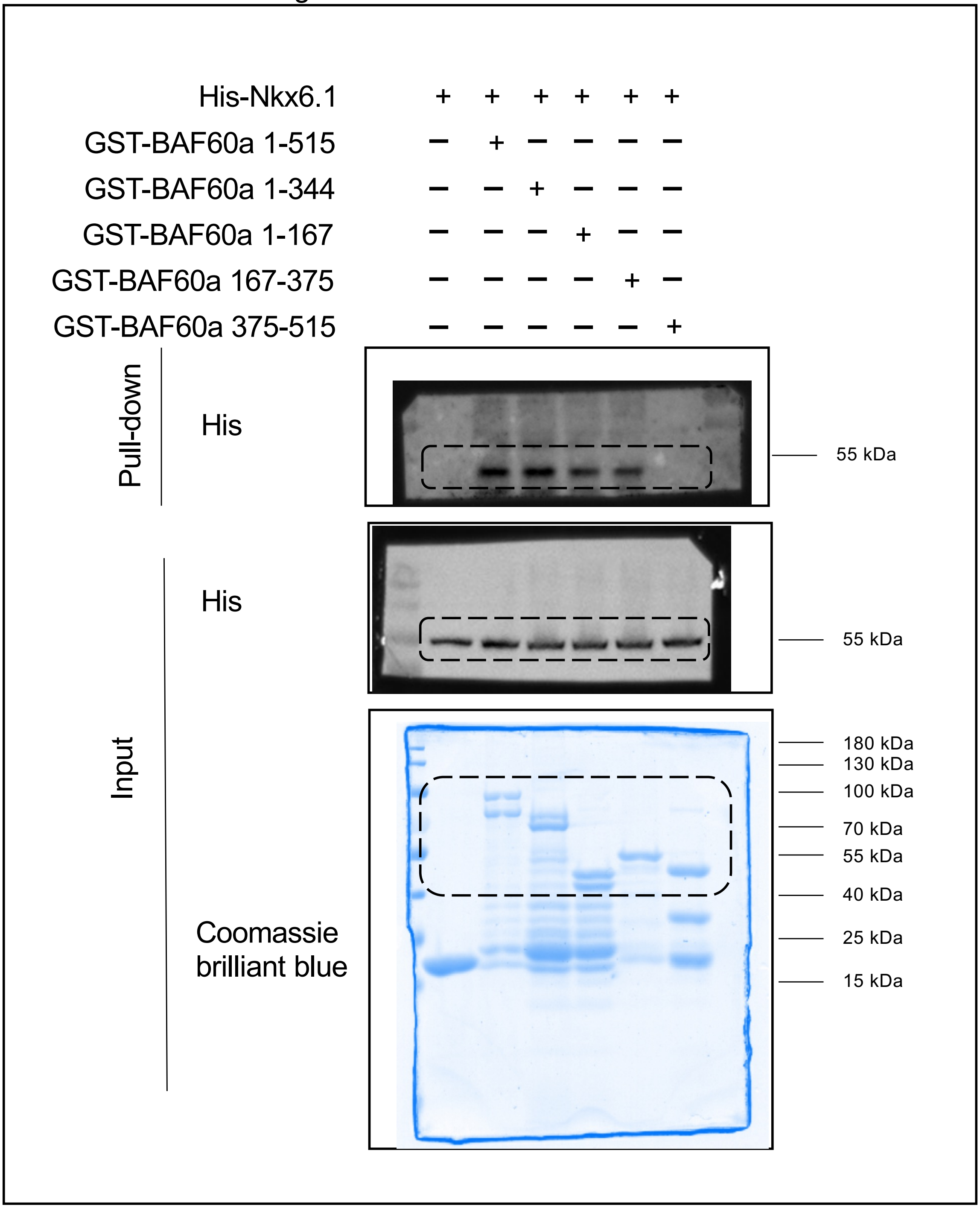

Full unedited blot for Fig 5.M

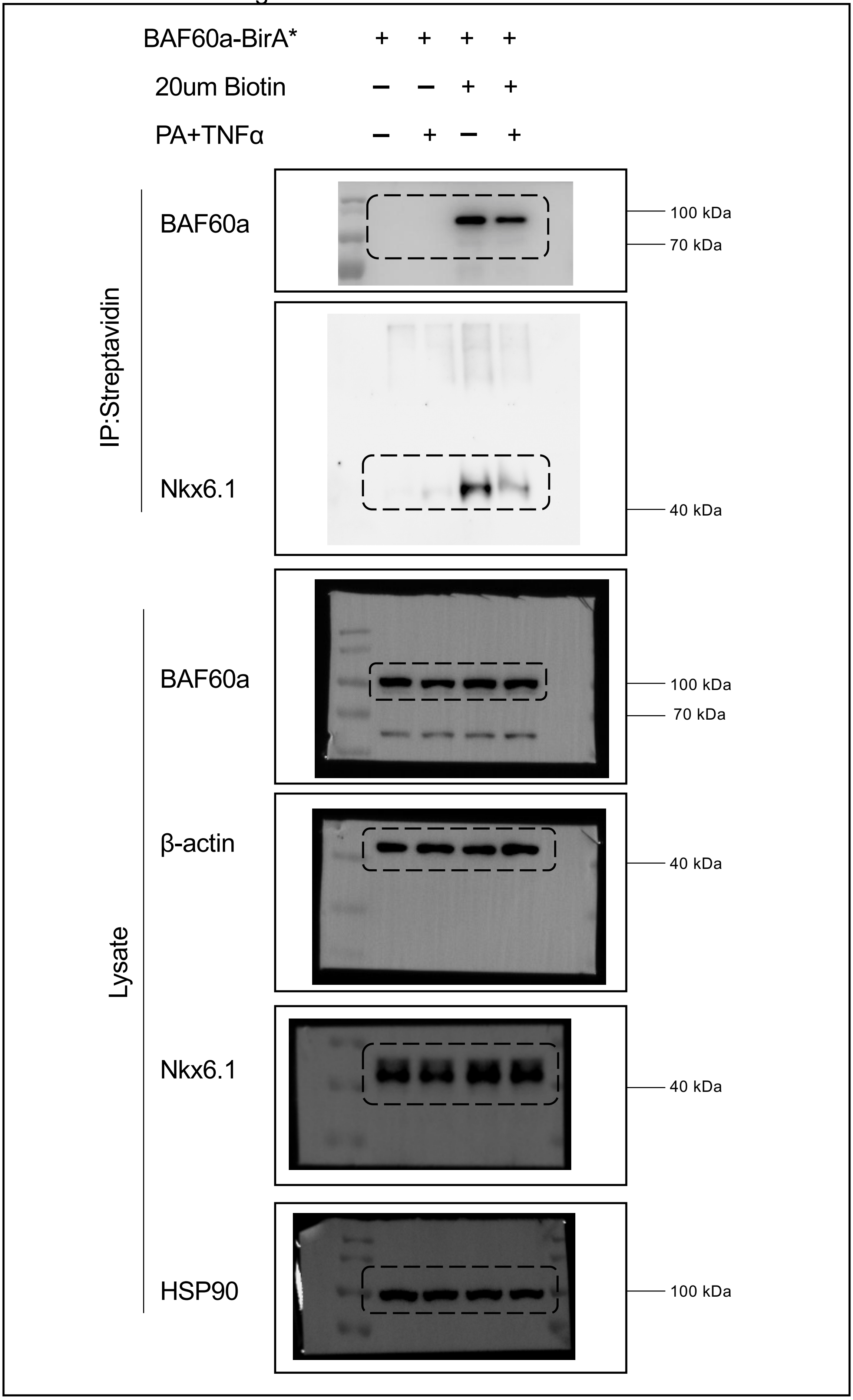

Full unedited blot for Fig 7.M

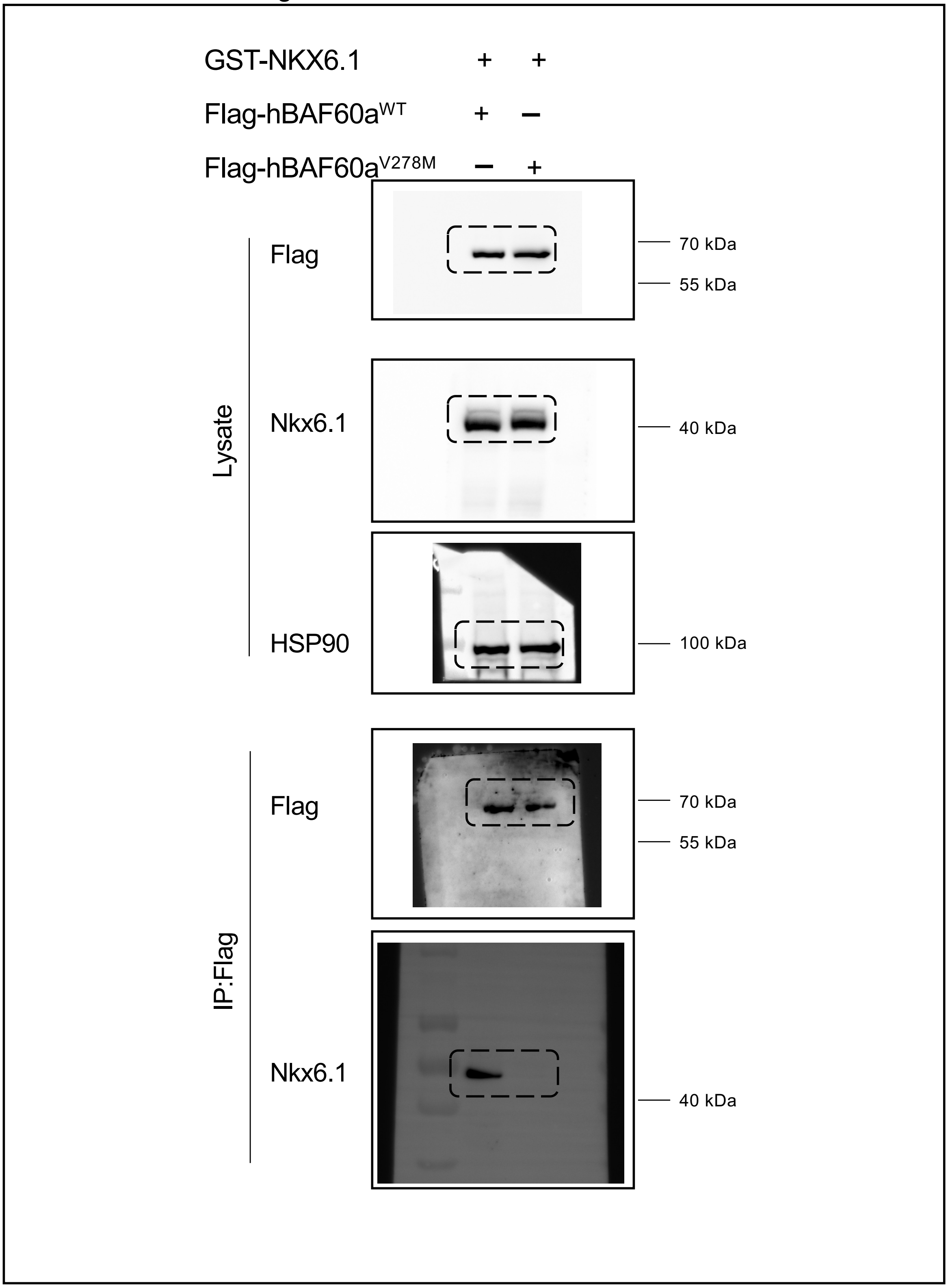

Full unedited blot for Fig 7.P

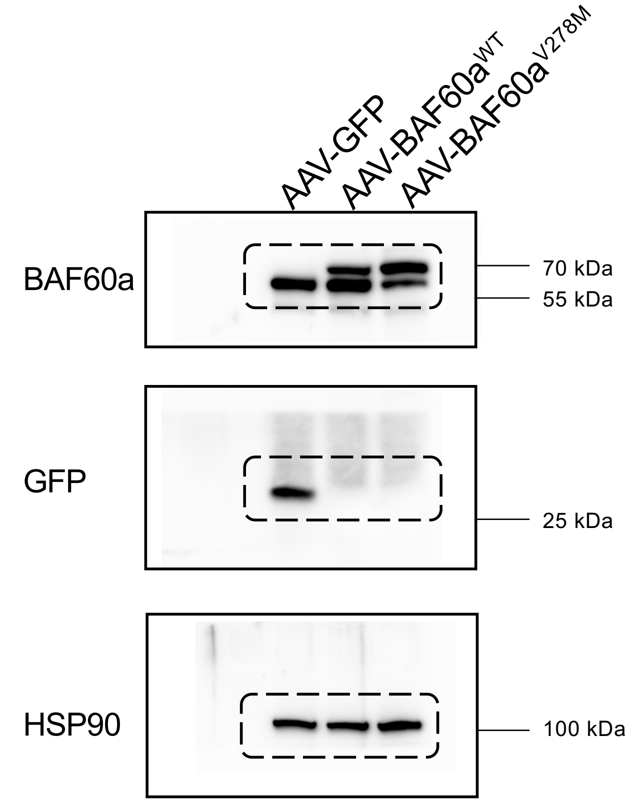

Full unedited blot for Fig 8.E

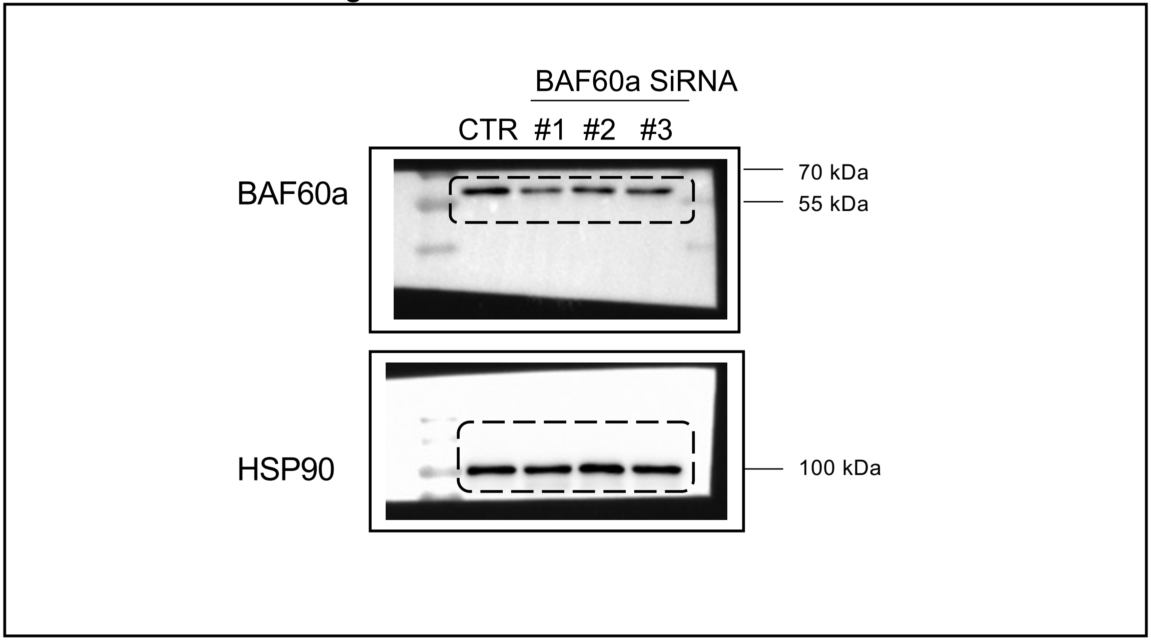

Full unedited blot for Fig S2.L

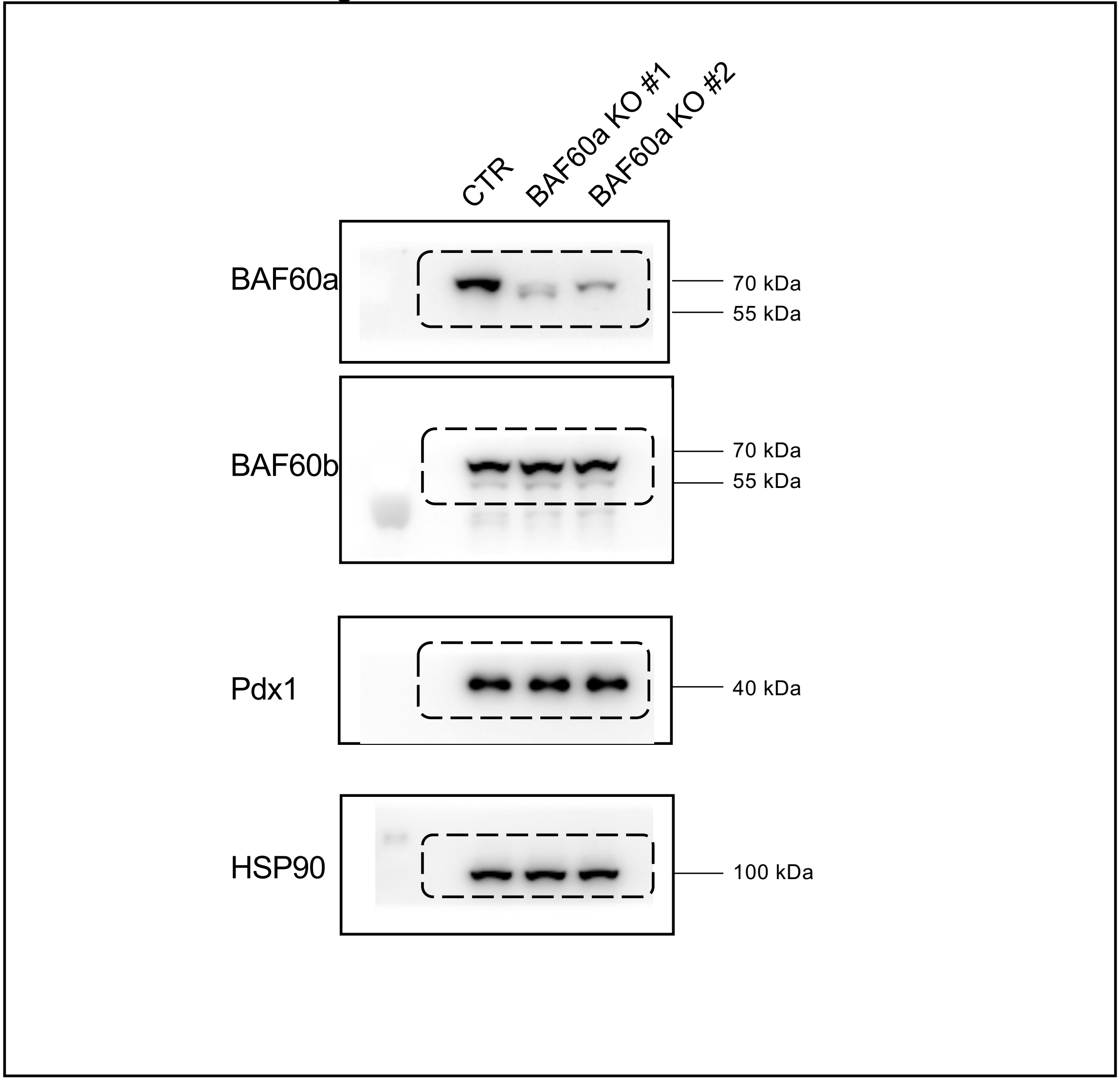

Full unedited blot for Fig S2.M

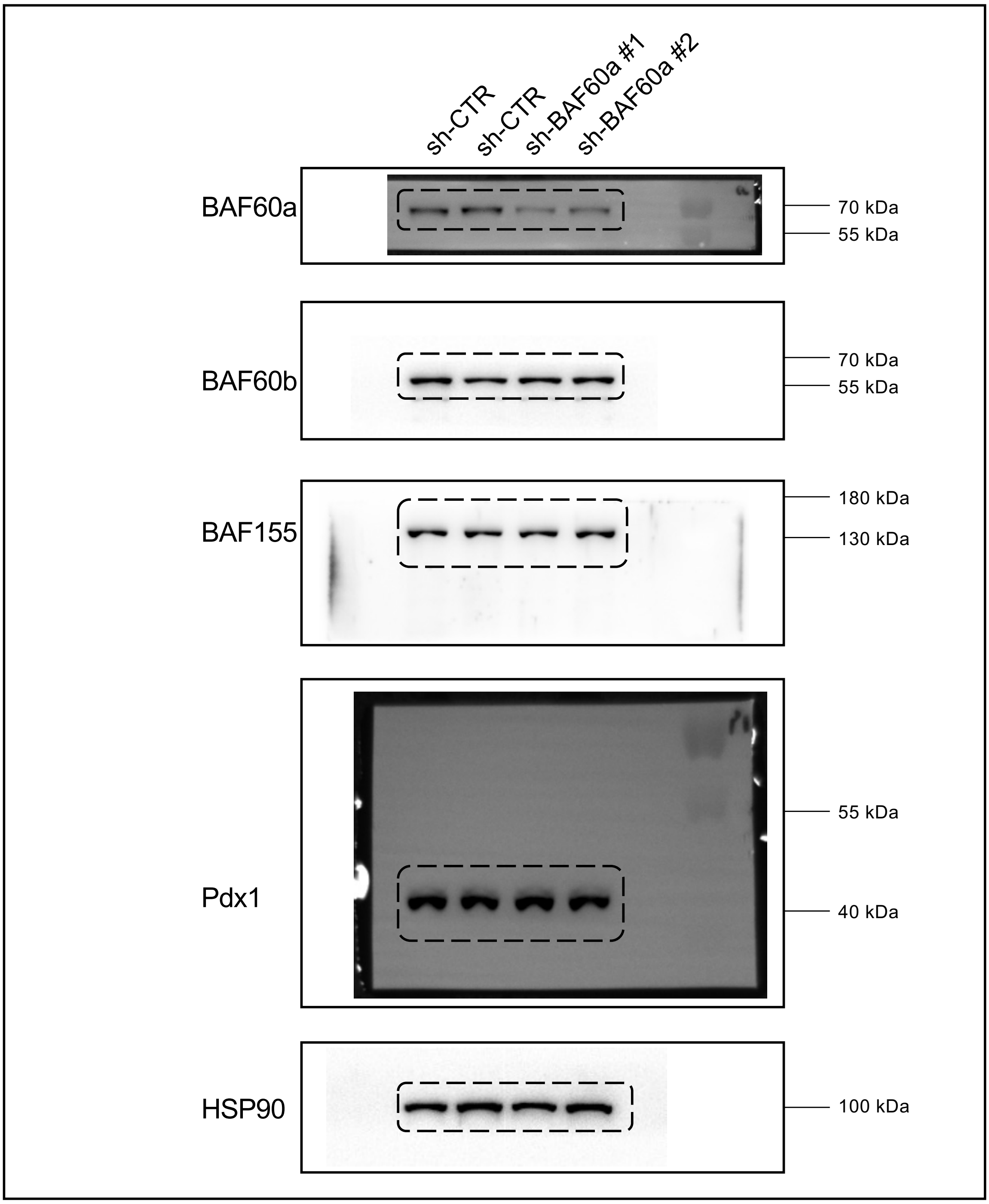

Full unedited blot for Fig S4.A

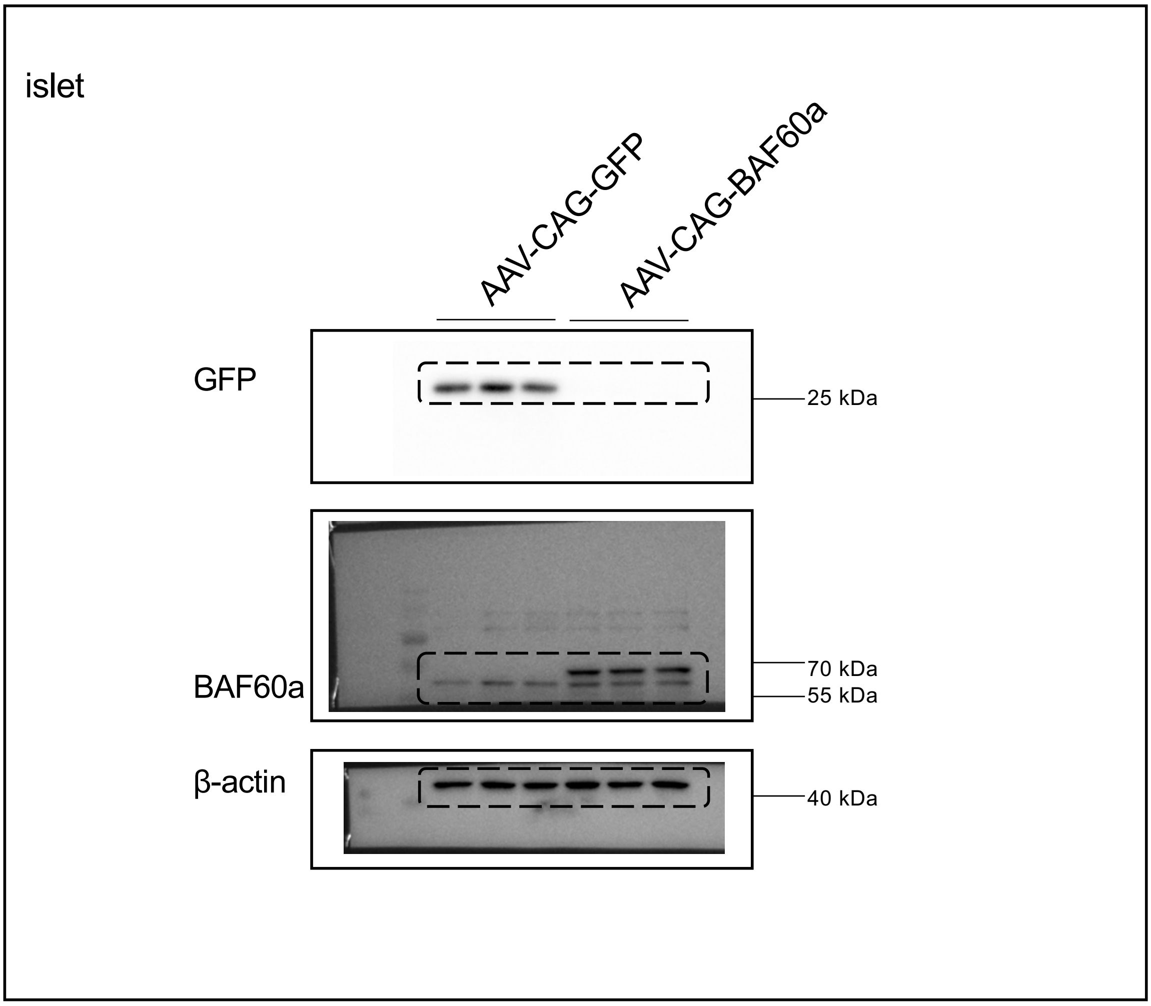

Full unedited blot for Fig S4.C

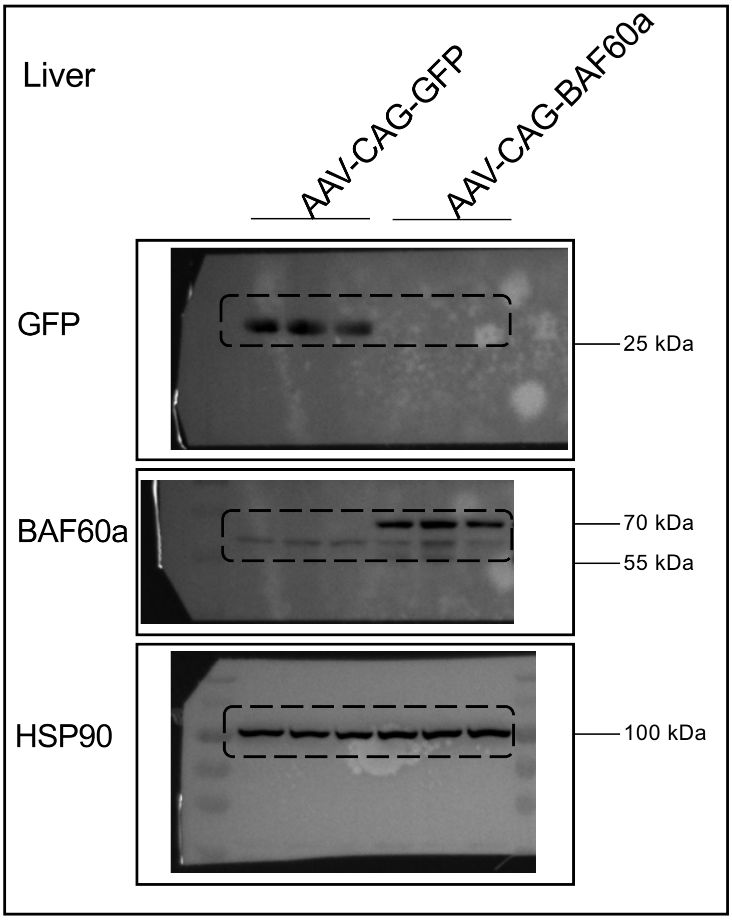

Full unedited blot for Fig S4.D

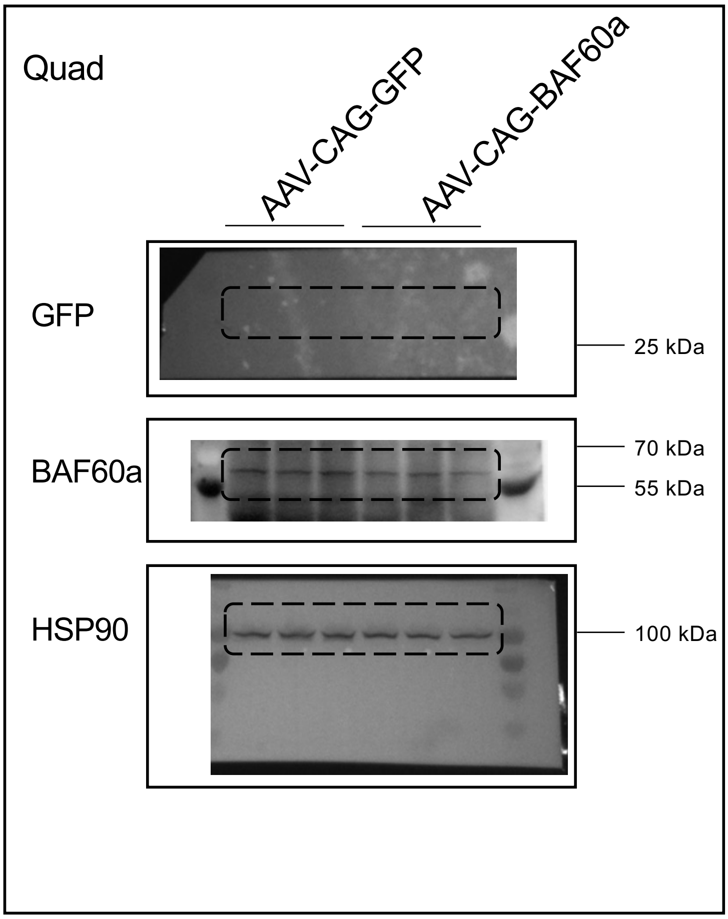

Full unedited blot for Fig S4.E

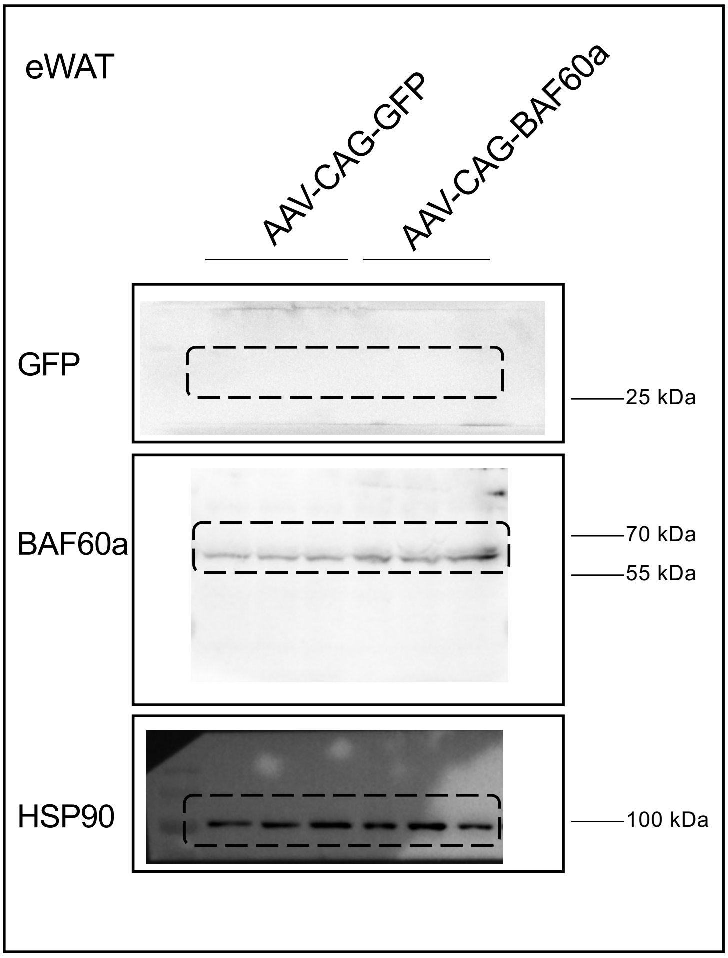

Full unedited blot for Fig S4.F

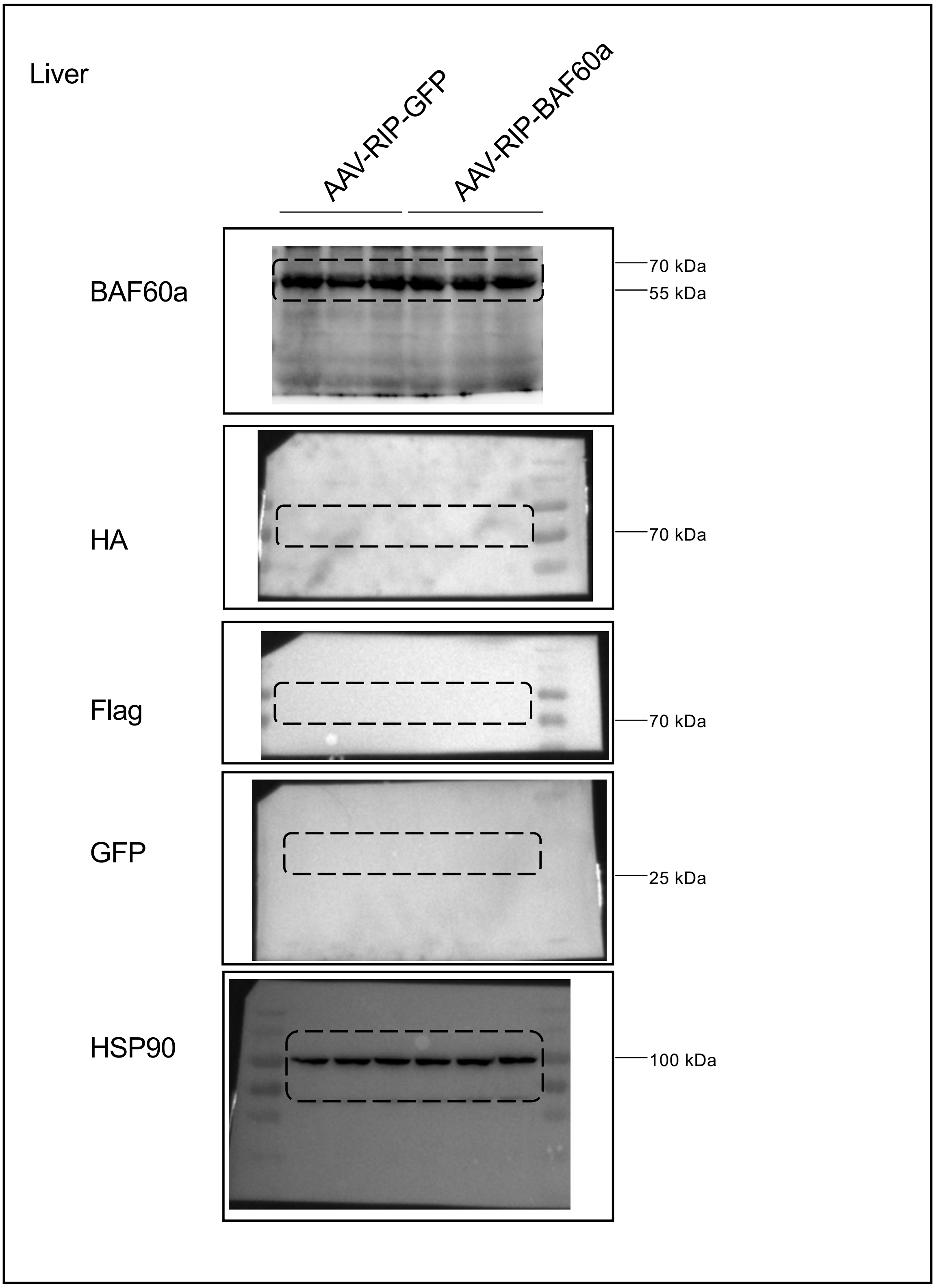

Full unedited blot for Fig S4.G

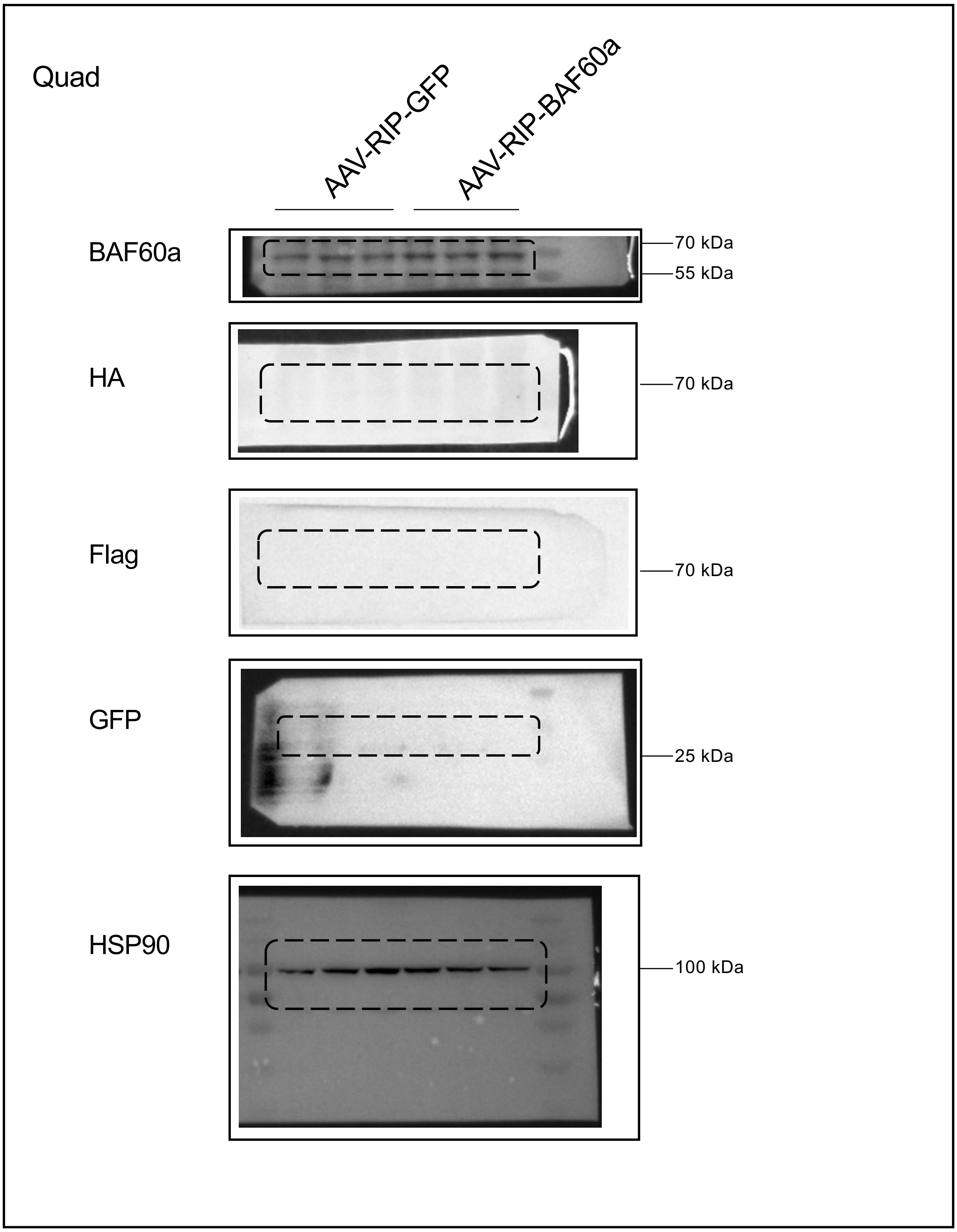

Full unedited blot for Fig S4.H

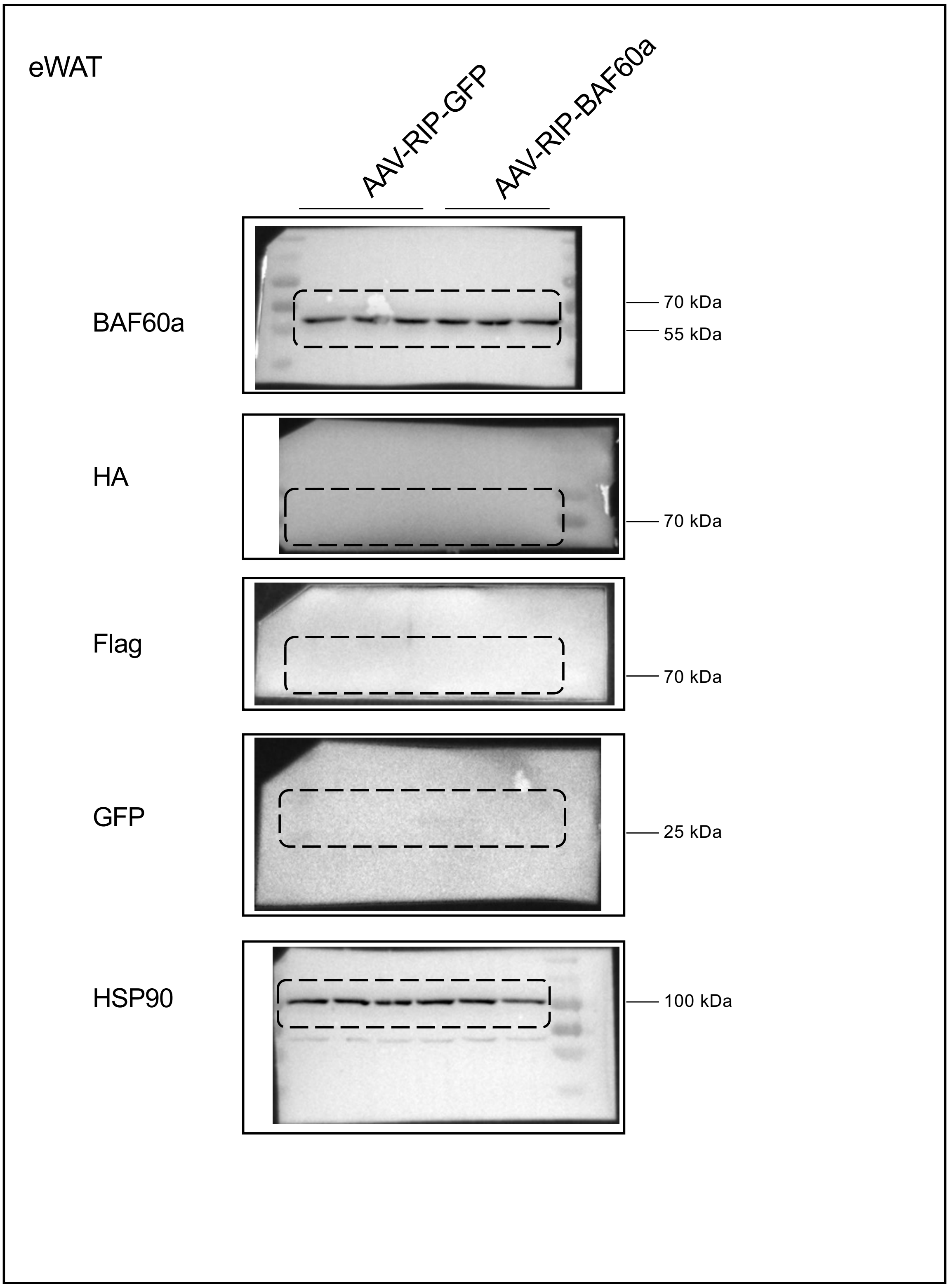

Full unedited blot for Fig S4.I

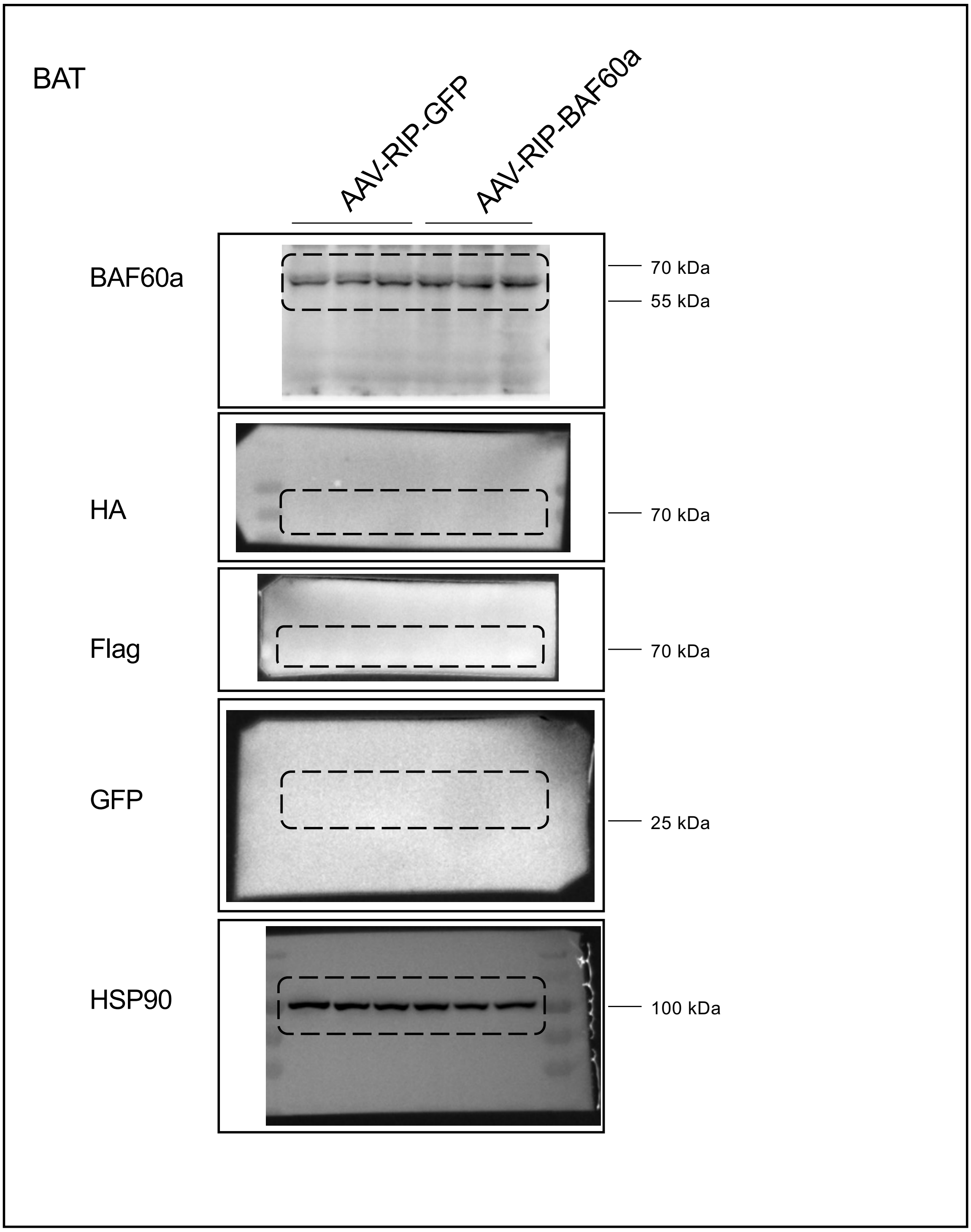

Full unedited blot for Fig S5.A

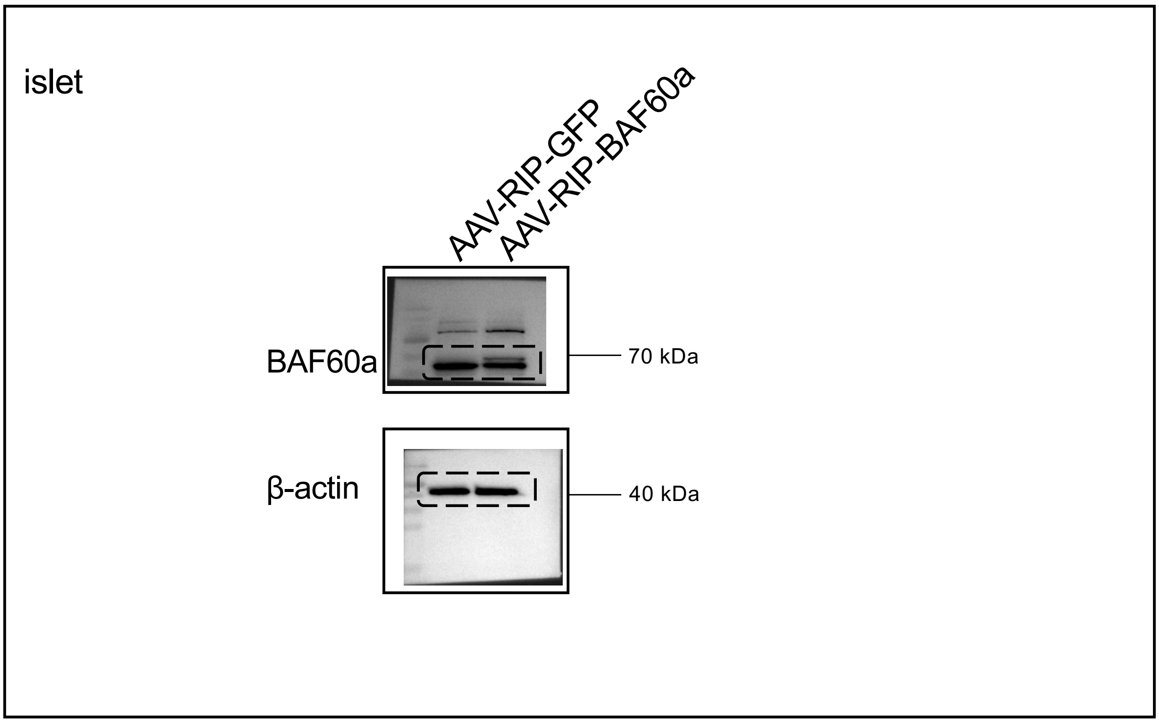

Full unedited blot for Fig S6.C

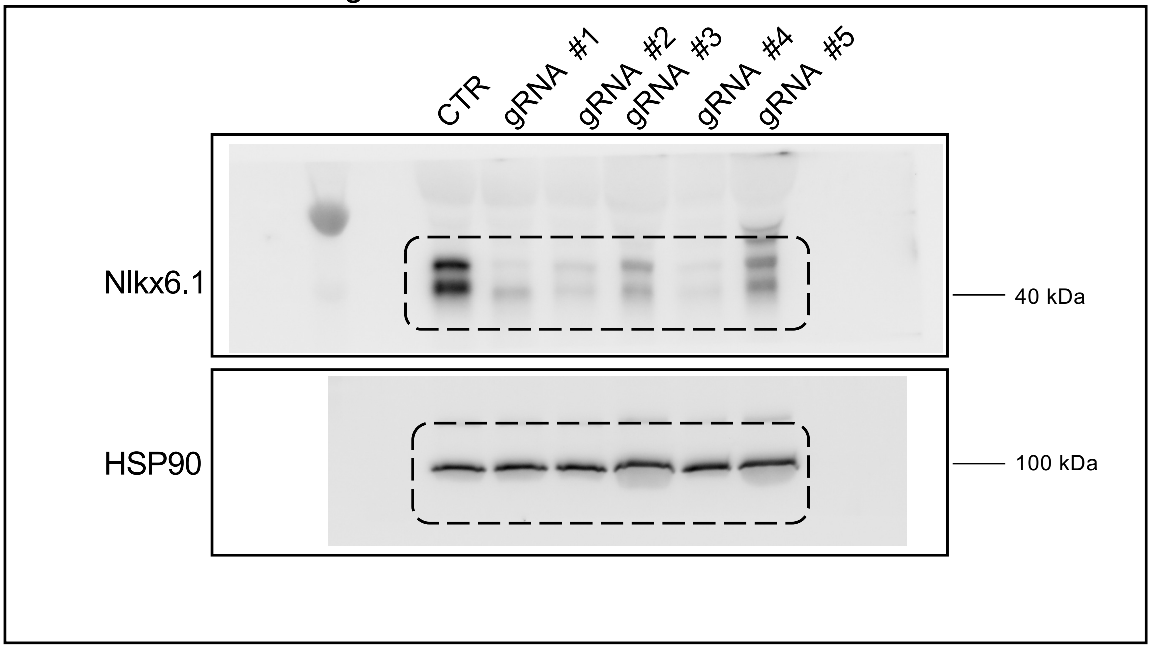

Full unedited blot for Fig S6.G

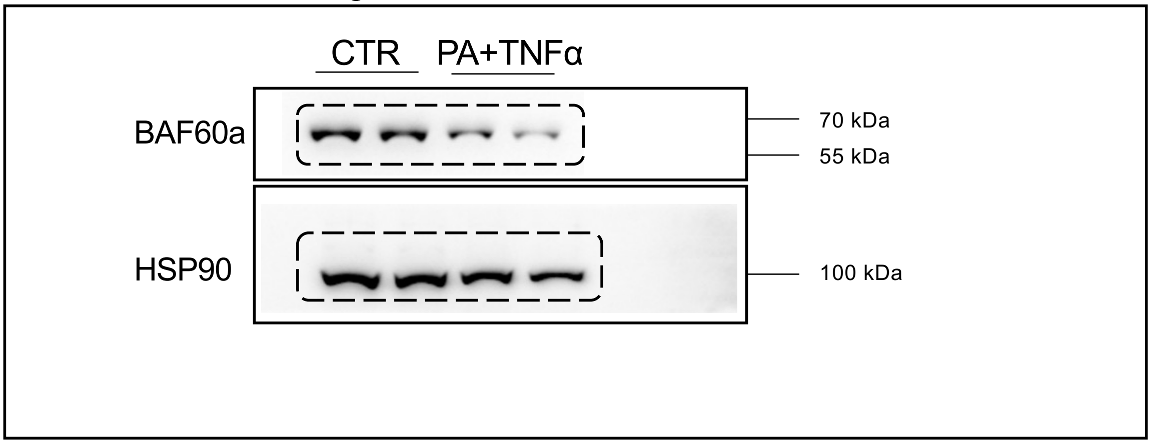

Full unedited blot for Fig S7.A

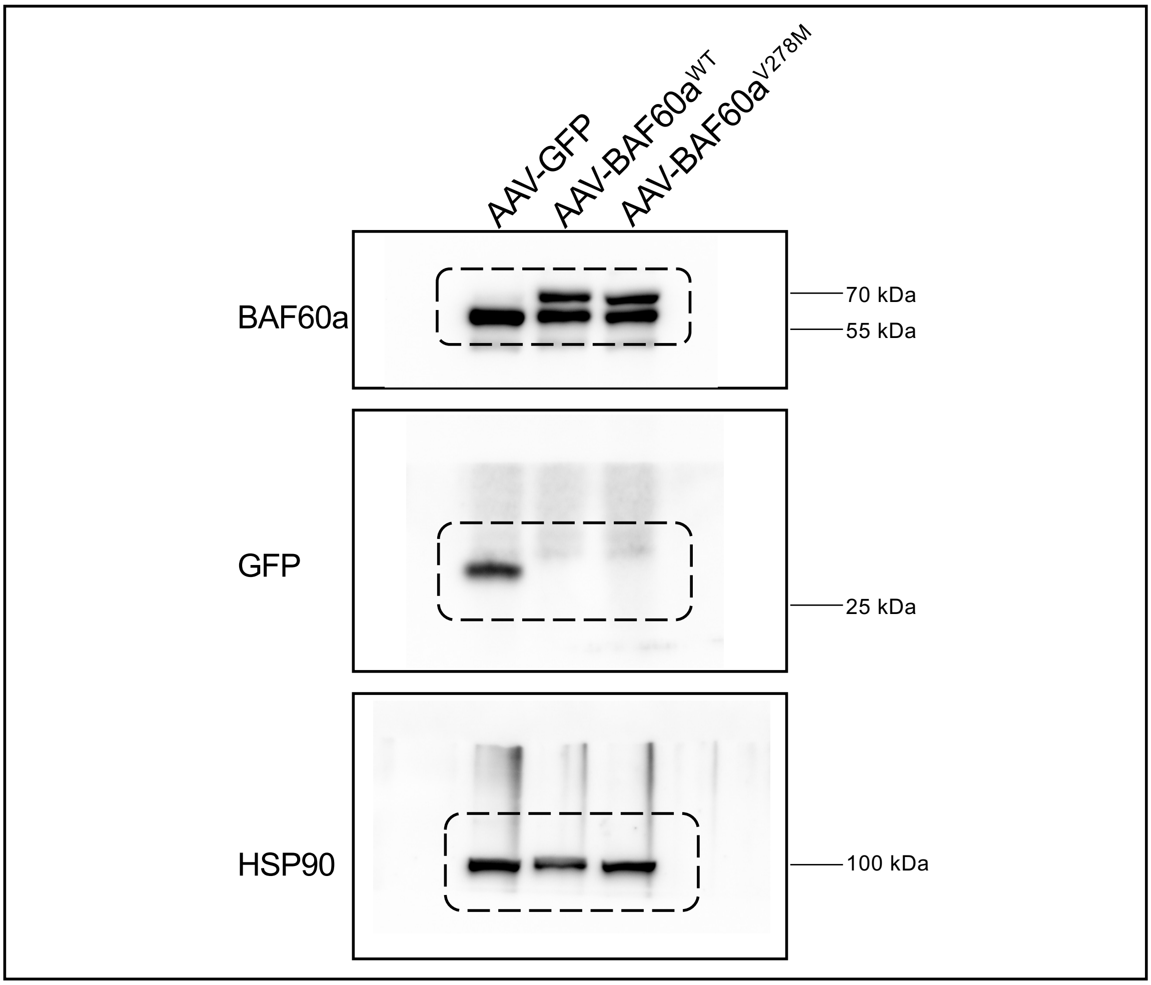

Supplement: Unedited blot and gel images [file jci-135-177980-s313.pdf]
